# Supplementary material for: An alternative splicing signature defines the basal-like phenotype and predicts worse clinical outcome in pancreatic cancer
Source: Cell Rep Med. 2024 Feb 6;5(2):101411. doi: 10.1016/j.xcrm.2024.101411 (PMC10897606; doi:10.1016/j.xcrm.2024.101411)
Supplement: Document S1. Figures S1–S7 [file mmc1.pdf]

Cell Reports Medicine, Volume *5*

Supplemental information

An alternative splicing signature defines the basal-like phenotype and predicts worse clinical outcome in pancreatic cancer

Veronica Ruta, Chiara Naro, Marco Pieraccioli, Adriana Leccese, Livia Archibugi, Eleonora Cesari, Valentina Panzeri, Chantal Allgöwer, Paolo Giorgio Arcidiacono, Massimo Falconi, Carmine Carbone, Giampaolo Tortora, Federica Borrelli, Fabia Attili, Cristiano Spada, Giuseppe Quero, Sergio Alfieri, Claudio Doglioni, Alexander Kleger, Gabriele Capurso, and Claudio Sette

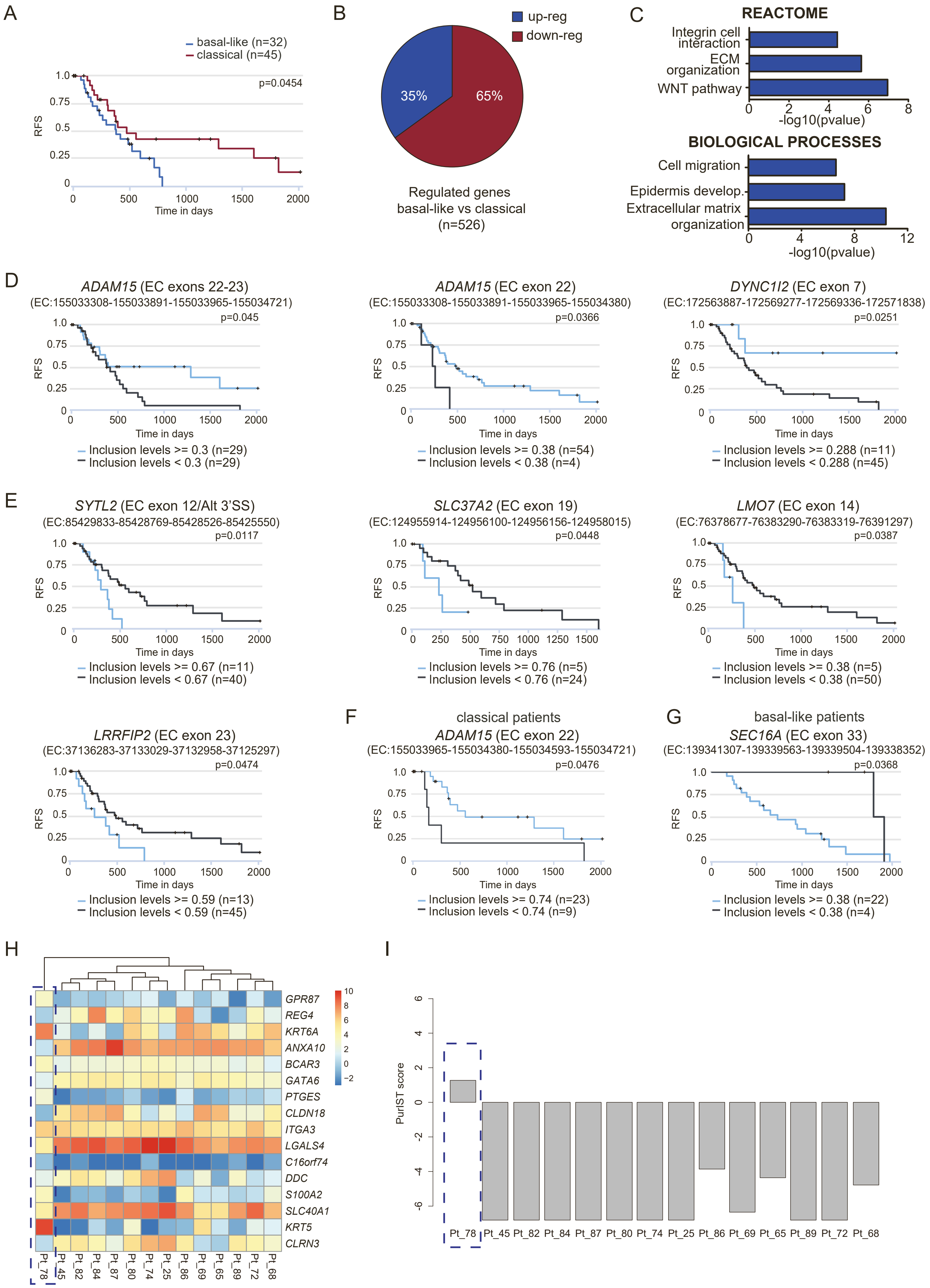
A
basal-like (n=32)
classical (n=45)
p=0.0454
RFS
Time in days
B
up-reg
down-reg
35%
65%
Regulated genes
basal-like vs classical
(n=526)
C
REACTOME
Integrin cell interaction
ECM organization
WNT pathway
-log10(pvalue)
BIOLOGICAL PROCESSES
Cell migration
Epidermis develop.
Extracellular matrix organization
-log10(pvalue)
D
ADAM15 (EC exons 22-23)
(EC:155033308-155033891-155033965-155034721)
p=0.045
Inclusion levels >= 0.3 (n=29)
Inclusion levels < 0.3 (n=29)
ADAM15 (EC exon 22)
(EC:155033308-155033891-155033965-155034380)
p=0.0366
Inclusion levels >= 0.38 (n=54)
Inclusion levels < 0.38 (n=4)
DYNC1I2 (EC exon 7)
(EC:172563887-172569277-172569336-172571838)
p=0.0251
Inclusion levels >= 0.288 (n=11)
Inclusion levels < 0.288 (n=45)
E
SYTL2 (EC exon 12/Alt 3'SS)
(EC:85429833-85428769-85428526-85425550)
p=0.0117
Inclusion levels >= 0.67 (n=11)
Inclusion levels < 0.67 (n=40)
SLC37A2 (EC exon 19)
(EC:124955914-124956100-124956156-124958015)
p=0.0448
Inclusion levels >= 0.76 (n=5)
Inclusion levels < 0.76 (n=24)
LMO7 (EC exon 14)
(EC:76378677-76383290-76383319-76391297)
p=0.0387
Inclusion levels >= 0.38 (n=5)
Inclusion levels < 0.38 (n=50)
LRRFIP2 (EC exon 23)
(EC:37136283-37133029-37132958-37125297)
p=0.0474
Inclusion levels >= 0.59 (n=13)
Inclusion levels < 0.59 (n=45)
F
classical patients
ADAM15 (EC exon 22)
(EC:155033965-155034380-155034593-155034721)
p=0.0476
Inclusion levels >= 0.74 (n=23)
Inclusion levels < 0.74 (n=9)
G
basal-like patients
SEC16A (EC exon 33)
(EC:139341307-139339563-139339504-139338352)
p=0.0368
Inclusion levels >= 0.38 (n=22)
Inclusion levels < 0.38 (n=4)
H
GPR87
REG4
KRT6A
ANXA10
BCAR3
GATA6
PTGES
CLDN18
ITGA3
LGALS4
C16orf74
DDC
S100A2
SLC40A1
KRT5
CLRN3
Pt_78 Pt_45 Pt_82 Pt_84 Pt_87 Pt_80 Pt_74 Pt_25 Pt_86 Pt_69 Pt_65 Pt_89 Pt_72 Pt_68
I
PurIST score
Pt_78 Pt_45 Pt_82 Pt_84 Pt_87 Pt_80 Pt_74 Pt_25 Pt_86 Pt_69 Pt_65 Pt_89 Pt_72 Pt_68

Figure S1: Subtype-specific genes and splicing events in PDAC patients. Related to Figures 1 and 2.
A) Kaplan-Meier curve displaying the RFS of PDAC patients from the whole TCGA cohort (n=77) segregated for classical and basal-like patients. **B)** Pie chart of the genes that are up-regulated (blue) and down-regulated (red) in basal-like vs classical PDAC. **C)** Pathways analysis by REACTOME and Gene Ontology analysis of Biological Processes of the genes that are up-regulated in basal-like PDAC. **D-E)** Kaplan-Meier curve displaying the RFS of PDAC patients from the whole TCGA cohort segregated for inclusion (blue line) or skipping (black line) of the indicated classical (**D**) and basal-like (**E**) exons. **F)** Kaplan-Meier curve displaying the RFS of classical PDAC patients from TCGA segregated for inclusion (blue line) or skipping (black line) of the *ADAM15* exon 22. **G)** Kaplan-Meier curve displaying the RFS of basal-like PDAC patients from TCGA segregated for inclusion (blue line) or skipping (black line) of the *SEC16A* exon 33. **H)** Heatmap showing subtype analysis in our cohort of EUS-TA PDAC patients (n=15) based on Moffitt's classification. **I)** The histogram represents the Purist score in each samples. **H,I)** The blue dashed boxes highlight the basal-like patient #78.

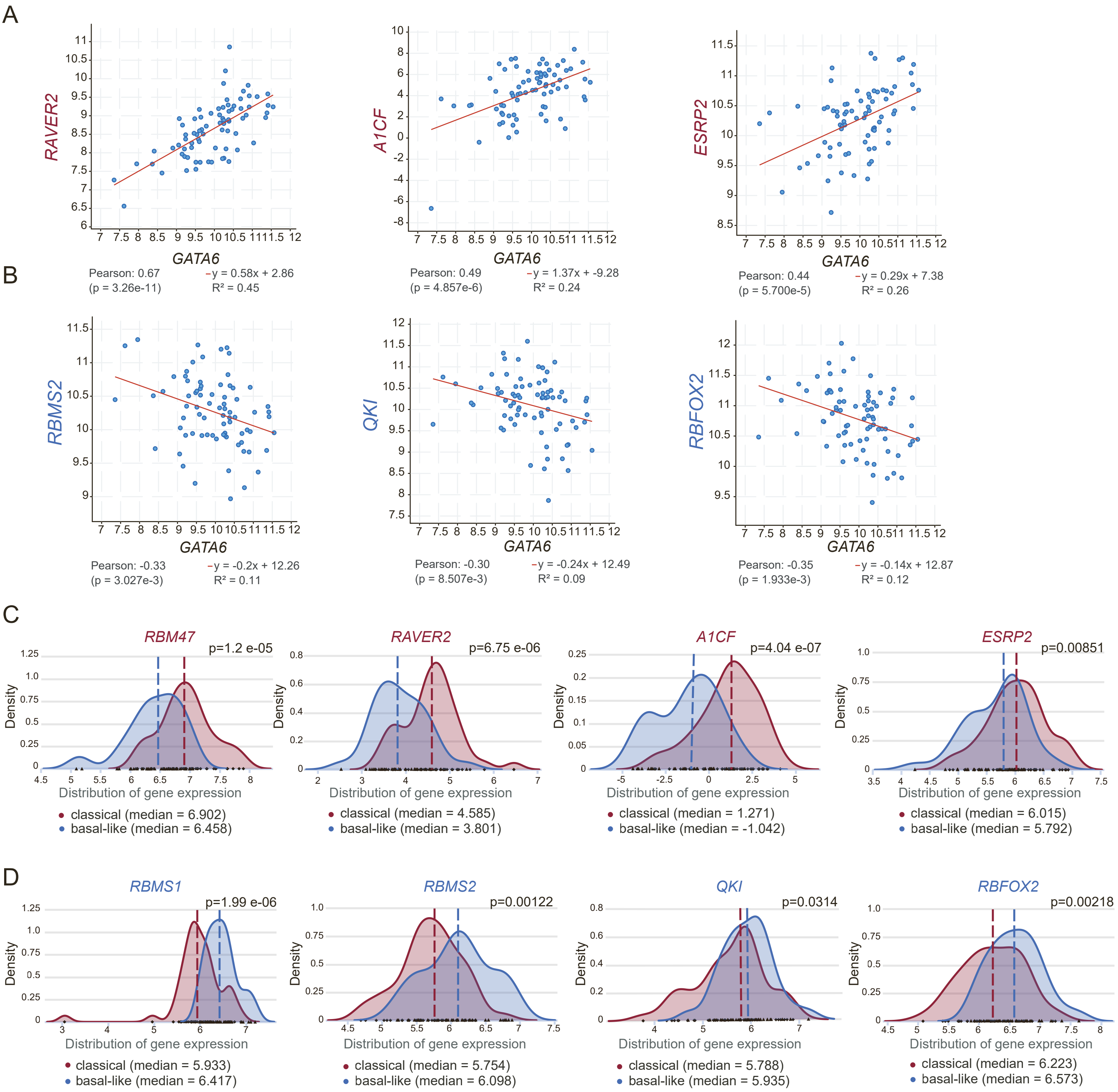

Figure S2: RBPs expression in PDAC patients and PDAC cell lines. Related to Figure 3.
A-B) Correlation analysis between the expression of classical (**A**) and basal-like (**B**) RBPs and that of GATA6. Statistical analysis was performed by 2-sided t-test. **C-D)** Density plot of the expression of classical (**C**) and basal-like (**D**) RBPs in classical (red) and basal-like (blue) PDAC patients from TCGA. Statistical analysis was performed by the Kruskal-Wallis rank sum test.

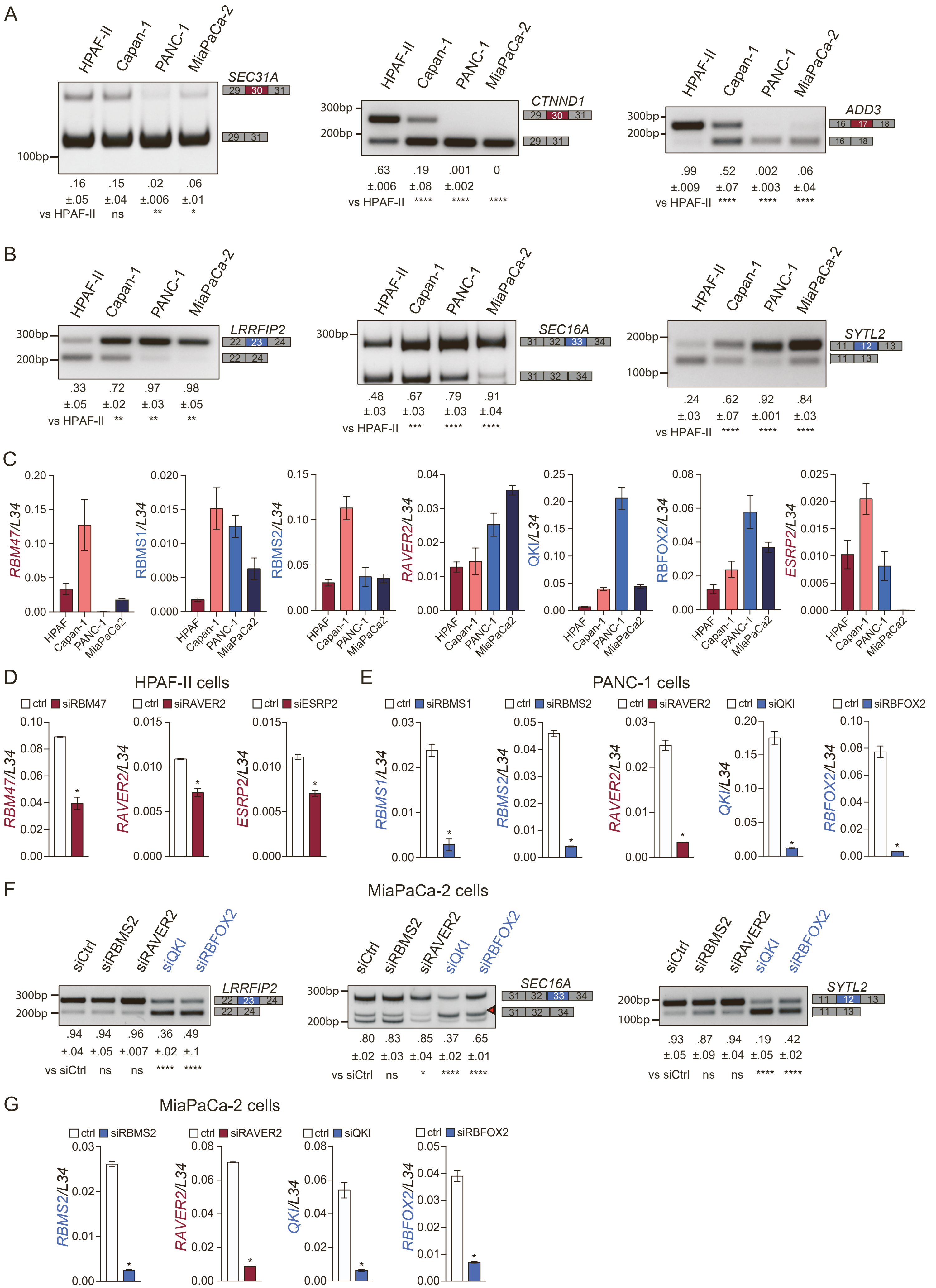
A
HPAF-II
Capan-1
PANC-1
MiaPaCa-2
SEC31A
29 30 31
29 31
100bp
.16 .15 .02 .06
±.05 ±.04 ±.006 ±.01
vs HPAF-II ns ** *
CTNND1
300bp
200bp
29 30 31
29 31
.63 .19 .001 0
±.006 ±.08 ±.002
vs HPAF-II **** **** ****
ADD3
16 17 18
16 18
.99 .52 .002 .06
±.009 ±.07 ±.003 ±.04
vs HPAF-II **** **** ****
B
LRRFIP2
22 23 24
22 24
.33 .72 .97 .98
±.05 ±.02 ±.03 ±.05
vs HPAF-II ** ** **
SEC16A
31 32 33 34
31 32 34
.48 .67 .79 .91
±.03 ±.03 ±.03 ±.04
vs HPAF-II *** **** ****
SYTL2
11 12 13
11 13
.24 .62 .92 .84
±.03 ±.07 ±.001 ±.03
vs HPAF-II **** **** ****
C
RBM47/L34
RBMS1/L34
RBMS2/L34
RAVER2/L34
QKI/L34
RBFOX2/L34
ESRP2/L34
HPAF
Capan-1
PANC-1
MiaPaCa2
D
HPAF-II cells
ctrl siRBM47
ctrl siRAVER2
ctrl siESRP2
E
PANC-1 cells
ctrl siRBMS1
ctrl siRBMS2
ctrl siRAVER2
ctrl siQKI
ctrl siRBFOX2
F
MiaPaCa-2 cells
siCtrl
siRBMS2
siRAVER2
siQKI
siRBFOX2
LRRFIP2
.94 .94 .96 .36 .49
±.04 ±.05 ±.007 ±.02 ±.1
vs siCtrl ns ns **** ****
SEC16A
.80 .83 .85 .37 .65
±.02 ±.03 ±.04 ±.02 ±.01
vs siCtrl ns * **** ****
SYTL2
.93 .87 .94 .19 .42
±.05 ±.09 ±.04 ±.05 ±.02
vs siCtrl ns ns **** ****
G
MiaPaCa-2 cells
ctrl siRBMS2
ctrl siRAVER2
ctrl siQKI
ctrl siRBFOX2

Figure S3: Subtype-specific splicing events are differentially regulated in PDAC cell lines. Related to Figure 3.
A-B) Splicing assay of the indicated representative classical (A) and basal-like (B) ECs in classical (HPAF-II and Capan-1) and basal-like (PANC-1 and MiaPaCa-2) PDAC cell lines. Data represent the mean ± standard deviation (SD) of three independent experiments. Statistical analysis was performed by Student's t-test; *p<0.05, **p<0.01, ***p<0.001, ****p<0.0001. **C)** Expression analysis of the indicated RBPs in the HPAF-II, Capan-1, PANC-1 and MiaPaCa-2 cell lines. Data represent the mean ± S.D. of three independent experiments. **D,E)** qRT-PCR analysis of the expression level of *RBM47*, *RAVER2* and *ESPR2* in HPAF-II cells silenced or not for the indicated RBP (**D**) and of *RBMS1*, *RBMS2*, *RAVER2*, *QKI* and *RBFOX2* in PANC-1 cells silenced or not for the indicated RBP (**E**). **F)** Splicing assay of the indicated basal-like ECs in MiaPaCa-2 cells silenced for the indicated RBPs. **G)** qRT-PCR analysis of the expression level of *RBMS2*, *RAVER2*, *QKI* and *RBFOX2* in MiaPaCa-2 cells silenced or not for the indicated RBP, normalized with *L34* expression.

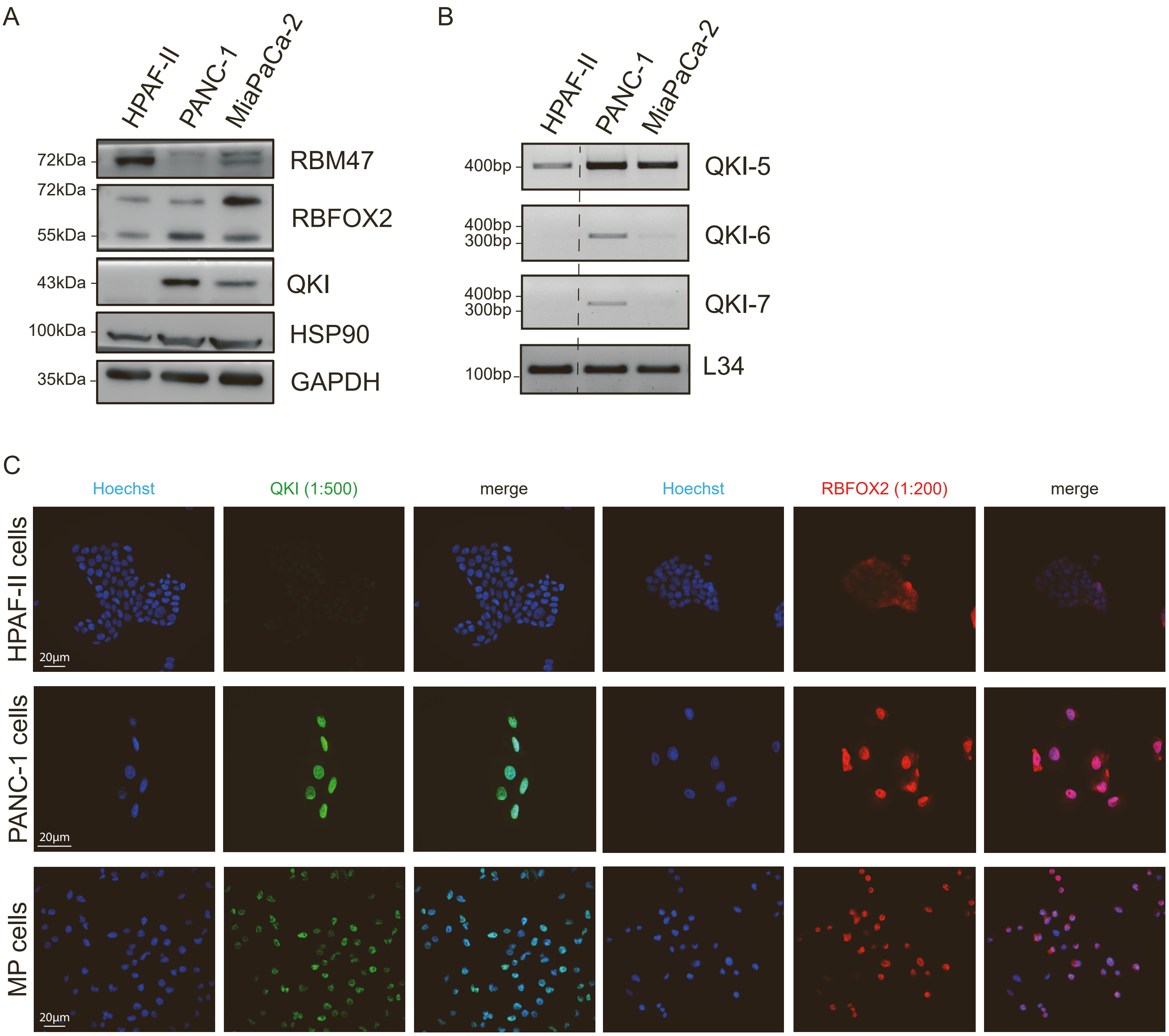

Figure S4: Expression of subtype specific RNA binding proteins in PDAC cell lines. Related to Figure 3 and 4.
A) Representative western blot analysis of the expression of RBM47, QKI and RBFOX2 in HPAF-II, PANC-1 and MiaPaCa-2 cells. HSP90 and GAPDH was used as loading controls. **B)** Representative RT-PCR analysis of the three QKI variants (QKI-5, QKI-6 and QKI-7) in HPAF-II, PANC-1 and MiaPaCa-2 cells. Dashed line indicates the border between images that have been computationally put in contact. **C)** Immunofluorescence analysis of the subcellular localization of QKI and RBFOX2 in HPAF-II, PANC-1 cells and MiaPaCa-2 cells. Scale bar=20µm.

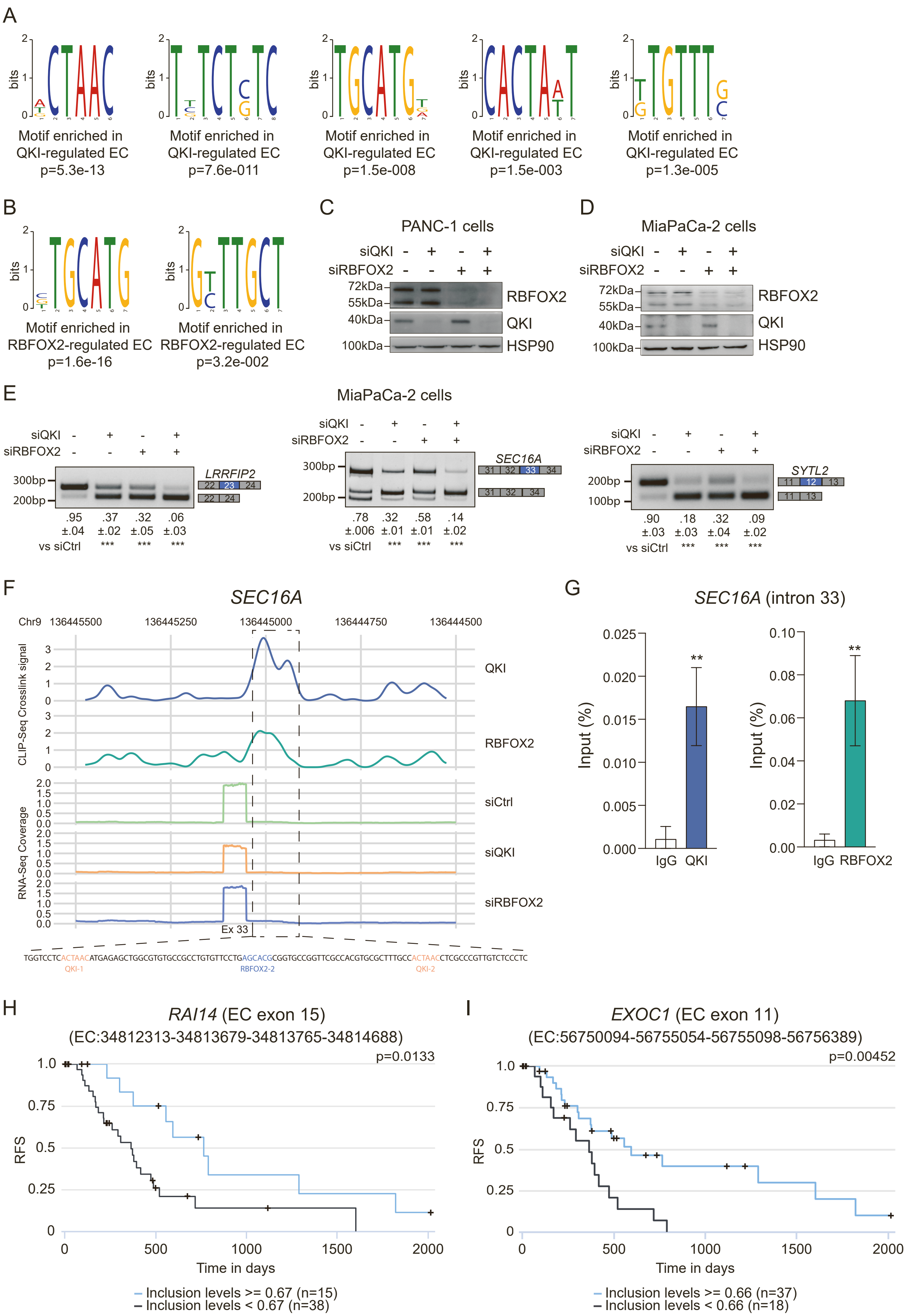
A
Motif enriched in QKI-regulated EC p=5.3e-13
Motif enriched in QKI-regulated EC p=7.6e-011
Motif enriched in QKI-regulated EC p=1.5e-008
Motif enriched in QKI-regulated EC p=1.5e-003
Motif enriched in QKI-regulated EC p=1.3e-005
B
Motif enriched in RBFOX2-regulated EC p=1.6e-16
Motif enriched in RBFOX2-regulated EC p=3.2e-002
C
PANC-1 cells
siQKI - + - +
siRBFOX2 - - + +
72kDa 55kDa RBFOX2
40kDa QKI
100kDa HSP90
D
MiaPaCa-2 cells
siQKI - + - +
siRBFOX2 - - + +
72kDa 55kDa RBFOX2
40kDa QKI
100kDa HSP90
E
MiaPaCa-2 cells
LRRFIP2
300bp 200bp
.95 ±.04 .37 ±.02 .32 ±.05 .06 ±.03
vs siCtrl *** *** ***
SEC16A
300bp 200bp
.78 ±.006 .32 ±.01 .58 ±.01 .14 ±.02
vs siCtrl *** *** ***
SYTL2
200bp 100bp
.90 ±.03 .18 ±.03 .32 ±.04 .09 ±.02
vs siCtrl *** *** ***
F
SEC16A
Chr9 136445500 136445250 136445000 136444750 136444500
CLIP-Seq Crosslink signal
QKI
RBFOX2
RNA-Seq Coverage
siCtrl
siQKI
siRBFOX2
Ex 33
TGGTCCTCACTAACATGAGAGCTGGCGTGTGCCGCCTGTGTTCCTGAGCACGCGGTGCCGGTTCGCCACGTGCGCTTTGCCACTAACCTCGCCCGTTGTCTCCCTC
QKI-1 RBFOX2-2 QKI-2
G
SEC16A (intron 33)
Input (%)
IgG QKI
IgG RBFOX2
H
RAI14 (EC exon 15)
(EC:34812313-34813679-34813765-34814688)
p=0.0133
RFS
Time in days
Inclusion levels >= 0.67 (n=15)
Inclusion levels < 0.67 (n=38)
I
EXOC1 (EC exon 11)
(EC:56750094-56755054-56755098-56756389)
p=0.00452
RFS
Time in days
Inclusion levels >= 0.66 (n=37)
Inclusion levels < 0.66 (n=18)

Figure S5: QKI and RBFOX2 bind intron 33 of SEC16A. Related to Figures 4, 5 and 6.
A-B) Logos representing the motifs significantly enriched in the regions flanking the ECs regulated by QKI (**A**) and RBFOX2 (**B**). **C-D)** Representative western blot analysis of the expression of RBFOX2 and QKI in PANC-1 and MiaPaCa-2 cells silenced or not for the indicated RBP. HSP90 was used as loading controls. **E)** Representative splicing assay (n=3) of the indicated basal-like ECs in MiaPaCa-2 cells silenced for QKI, RBFOX2 or both RBPs. Percentage of splicing inclusion ± SD (n=3) of the indicated exons was evaluated by densitometric analysis. Statistical analysis was performed by Student's t-test; ***p<0.001. **F)** Line plot showing mean normalized read coverage in two replicates of QKI and RBFOX2 eCLIP-seq using ENCODE project (upper panel) and mean normalized read coverage in three replicates of siCtrl, siQKI and siRBFOX2 RNA-seq (bottom panel) in a region encompassing the exon 33 region of the *SEC16A* gene. QKI and RBFOX2 binding region is highlighted with the dashed lines, their binding sites are highlighted in orange (QKI) and blue (RBFOX2). **G)** CLIP assay of QKI and RBFOX2 performed in PANC-1 cells. Immunoprecipitation with control IgGs was performed as negative control. RNA associated with *SEC16A* was quantified by qPCR using primers located at the *SEC16A* exon 33-intron 33 boundary. Results are represented as percentage (%) of input (mean ± SD of three biological replicates). Statistical analysis was performed by Student's t-test; **p<0.01. **H-I)** Kaplan-Meier curve displaying the RFS of PDAC patients from the TCGA cohort segregated for inclusion (blue line) or skipping (black line) of the *RAI14* (**H**) and *EXOC1* (**I**) ECs.

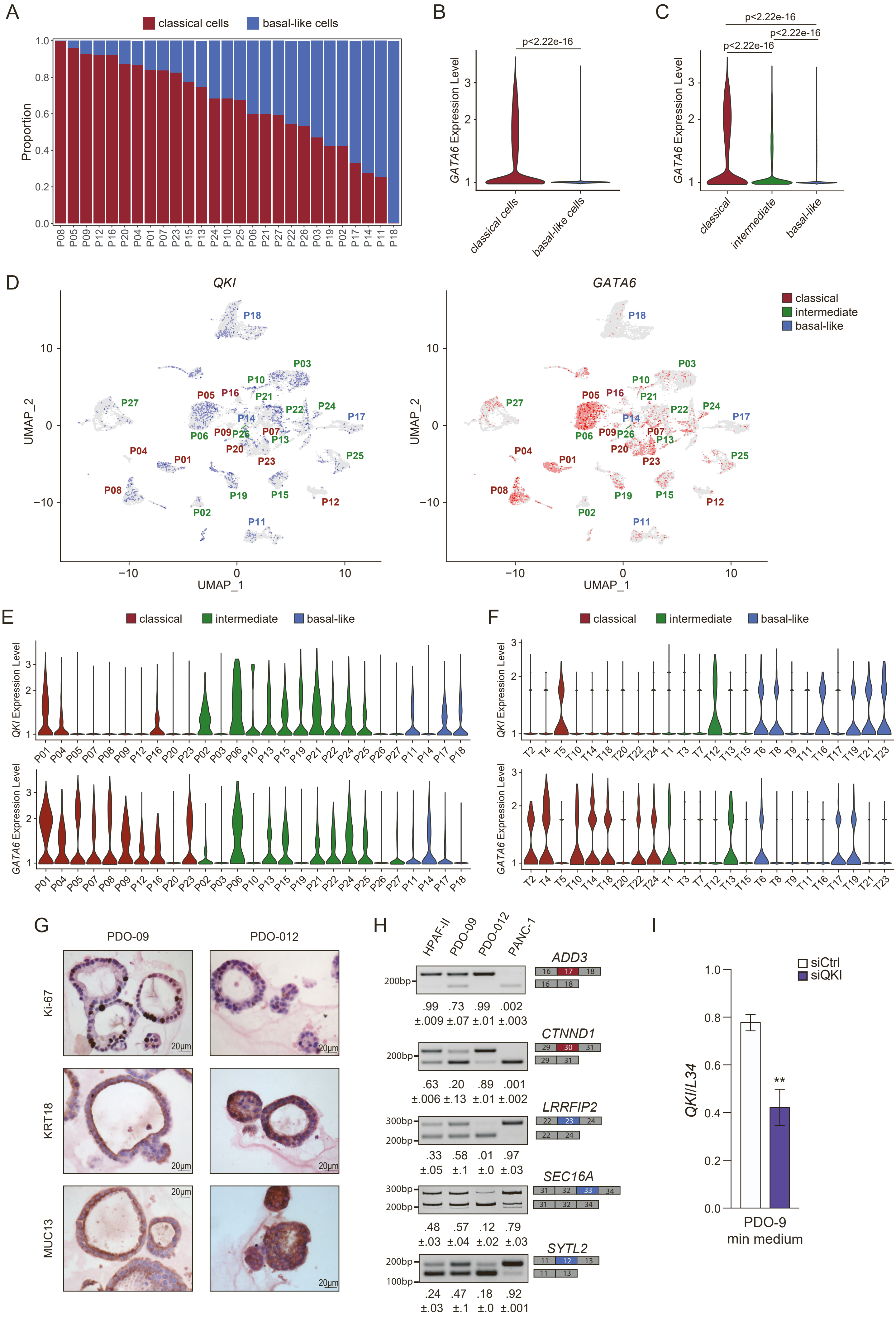
A
classical cells
basal-like cells
Proportion
B
p<2.22e-16
GATA6 Expression Level
classical cells
basal-like cells
C
classical
intermediate
basal-like
D
QKI
GATA6
UMAP_1
UMAP_2
E
QKI Expression Level
GATA6 Expression Level
F
G
PDO-09
PDO-012
Ki-67
KRT18
MUC13
20µm
H
HPAF-II
PDO-09
PDO-012
PANC-1
ADD3
.99 .73 .99 .002
±.009 ±.07 ±.01 ±.003
CTNND1
.63 .20 .89 .001
±.006 ±.13 ±.01 ±.002
LRRFIP2
.33 .58 .01 .97
±.05 ±.1 ±.0 ±.03
SEC16A
.48 .57 .12 .79
±.03 ±.04 ±.02 ±.03
SYTL2
.24 .47 .18 .92
±.03 ±.1 ±.0 ±.001
I
siCtrl
siQKI
QKI/L34
PDO-9
min medium

Figure S6: Single cell analysis identifies QKI as a marker of the basal-like identity in PDAC and subtype-specific events are correlated with QKI expression in PDAC patient-derived organoids. Related to Figure 6.

A) Proportion of classical and basal-like cells identified according to the Moffitt signature in PDAC samples from the GSE205013 dataset. **B)** Violin plot of the gene expression levels of GATA6 in classical and basal-like cell population as determined by single cell transcriptomic analysis (GSE205013). **C)** Violin plot showing of the gene expression levels of GATA6 in tumors that were classified as classical, intermediate and basal-like as determined by single cell transcriptomic analysis (GSE205013). **D)** UMAP overview of the QKI (left) and GATA6 expression (right) in PDAC samples that were classified as classical, intermediate and basal-like on the basis of single cell transcriptome analysis using the Moffit signature (GSE205013). **E-F)** Violin plot of the gene expression levels of QKI and GATA6 in classical, intermediate and basal-like PDAC samples patients on single cell transcriptome analysis of the GSE205013 (**E**) and CRA001160 (**F**) datasets. **G)** IHC of Ki-67, KRT18 and MUC13 on PDAC PDO-9 and PDO-12. Scale bar=20µm. **H)** Splicing assays of *ADD3* (exon 17), *CTNND1* (exon 30), *LRRFIP2* (exon 23), *SYTL2* (exon 12) in two patient-derived organoids (PDO). For comparison the classical HPAF-II and the PANC-1 cell lines were also analyzed. **I)** qRT-PCR analysis of the expression level of QKI in PDO silenced or not for QKI.

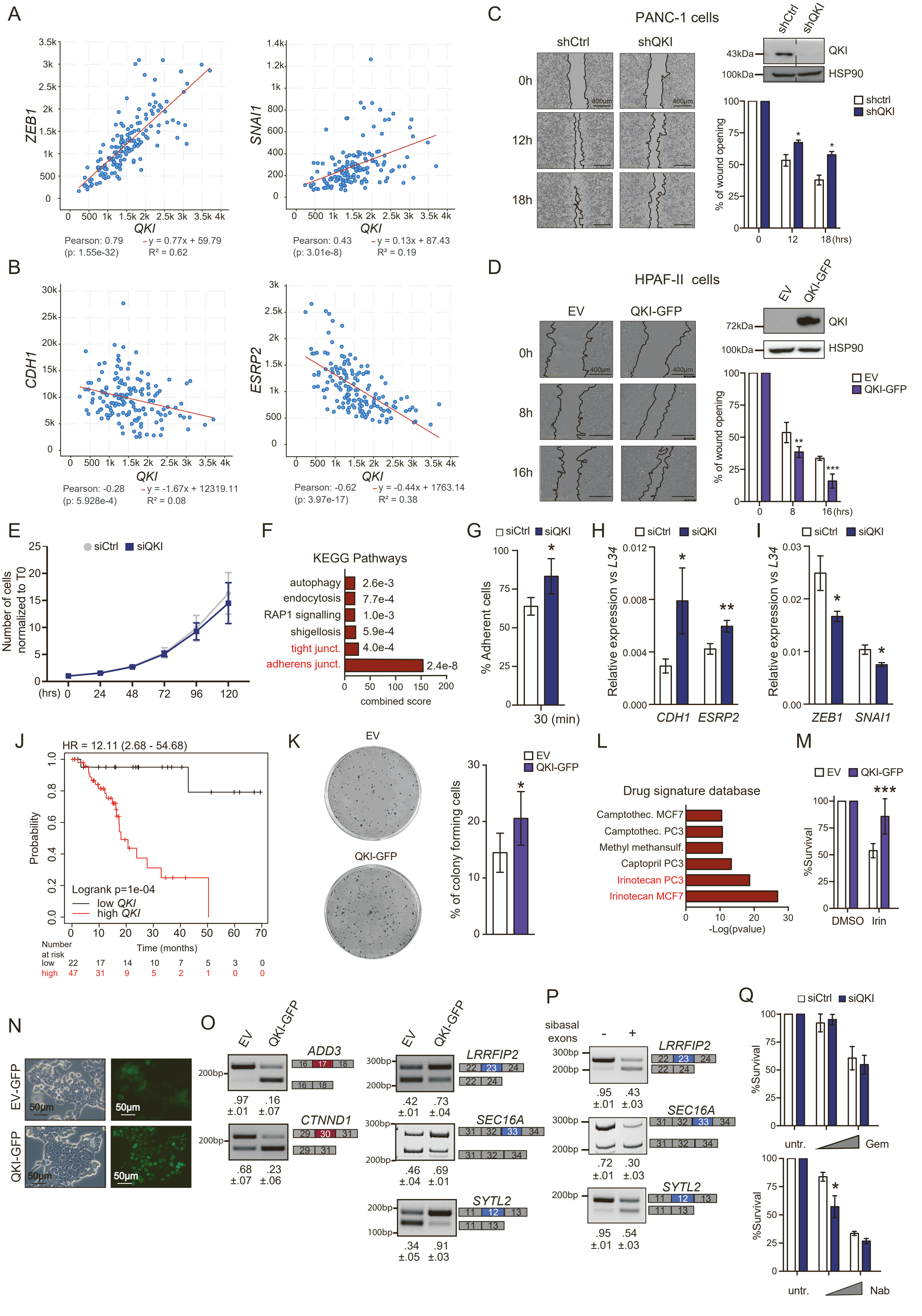
A
ZEB1
QKI
Pearson: 0.79
(p: 1.55e-32)
y = 0.77x + 59.79
R² = 0.62
SNAI1
QKI
Pearson: 0.43
(p: 3.01e-8)
y = 0.13x + 87.43
R² = 0.19
B
CDH1
QKI
Pearson: -0.28
(p: 5.928e-4)
y = -1.67x + 12319.11
R² = 0.08
ESRP2
QKI
Pearson: -0.62
(p: 3.97e-17)
y = -0.44x + 1763.14
R² = 0.38
C
PANC-1 cells
shCtrl
shQKI
0h
12h
18h
400µm
43kDa
QKI
100kDa
HSP90
shctrl
shQKI
% of wound opening
18 (hrs)
D
HPAF-II cells
EV
QKI-GFP
0h
8h
16h
72kDa
QKI
100kDa
HSP90
% of wound opening
16 (hrs)
E
siCtrl
siQKI
Number of cells normalized to T0
(hrs)
F
KEGG Pathways
autophagy 2.6e-3
endocytosis 7.7e-4
RAP1 signalling 1.0e-3
shigellosis 5.9e-4
tight junct. 4.0e-4
adherens junct. 2.4e-8
combined score
G
siCtrl
siQKI
% Adherent cells
30 (min)
H
siCtrl
siQKI
Relative expression vs L34
CDH1
ESRP2
I
siCtrl
siQKI
Relative expression vs L34
ZEB1
SNAI1
J
HR = 12.11 (2.68 - 54.68)
Probability
Logrank p=1e-04
low QKI
high QKI
Time (months)
Number at risk
low 22 17 14 10 7 5 3 0
high 47 31 9 5 2 1 0 0
K
EV
QKI-GFP
% of colony forming cells
L
Drug signature database
Camptothec. MCF7
Camptothec. PC3
Methyl methansulf.
Captopril PC3
Irinotecan PC3
Irinotecan MCF7
-Log(pvalue)
M
EV
QKI-GFP
%Survival
DMSO
Irin
N
EV-GFP
QKI-GFP
50µm
O
EV
QKI-GFP
ADD3
200bp
16 17 18
16 18
.97 ±.01
.16 ±.07
CTNND1
29 30 31
29 31
.68 ±.07
.23 ±.06
LRRFIP2
300bp
200bp
22 23 24
22 24
.42 ±.01
.73 ±.04
SEC16A
31 32 33 34
31 32 34
.46 ±.04
.69 ±.01
SYTL2
200bp
100bp
11 12 13
11 13
.34 ±.05
.91 ±.03
P
sibasal exons
-
+
LRRFIP2
300bp
200bp
22 23 24
22 24
.95 ±.01
.43 ±.03
SEC16A
31 32 33 34
31 32 34
.72 ±.01
.30 ±.03
SYTL2
11 12 13
11 13
.95 ±.01
.54 ±.03
Q
siCtrl
siQKI
%Survival
untr.
Gem
%Survival
untr.
Nab

Figure S7: QKI promotes the mesenchymal phenotype in PDAC cells. Related to Figure 7.

A-B) Correlation analysis between the expression of *QKI* and that of pro-mesenchymal transcription factors *ZEB1* and *SNAI1* (**A**) and the epithelial markers *CDH1* and *ESRP2* (**B**) in PDAC samples from TCGA patients. **C)** Representative images of wound-healing assays in PANC-1 cells stably silenced for QKI. The histograms (right) report the quantification of the wound area (mean ± SD, n=3). Statistical analysis was performed by Two-way Anova test; *p<0.05. Western blot analysis to assess QKI silencing is shown above the histogram. Scale bar=400µm. **D)** Representative images of wound-healing assays in HPAF-II cells stably overexpressed QKI. The histograms (right) report the quantification of the wound area (mean ± SD, n=3). Statistical analysis was performed by Two-way Anova test; **p<0.01; ***p<0.001. Western blot analysis to assess QKI overexpression is shown above the histogram. Scale bar=400µm. **E)** Line graph showing growth of PANC-1 cells silenced or not for QKI. **F)** KEGG pathways analysis of the genes regulated by QKI at splicing level. **G)** Histogram reporting the percentage of adherent cells in PANC-1 silenced or not for QKI expression. Data are reported as the mean ± S.D. of three independent experiments. Statistical analysis was performed by the Student's t-test; *p<0.05. **H-I)** qRT-PCR showing the expression of the epithelial markers *CDH1*, *ESRP2* (**H**) and EMT-inducers *ZEB1*, *SNAI1* (**I**) in PANC-1 cells silenced or not for QKI (mean ± SD, n=3). Statistical analysis was performed by using the Student's t-test (*p<0.05; **p<0.01). **J)** Kaplan-Meier curve displaying overall survival of TCGA PDAC patients exhibiting high (upper quartiles; red line) or low (lower quartiles; black line) expression of QKI. **K)** Representative image of the colonies formed by HPAF-II cells expressing (QKI-GFP) or not (empty vector, EV) QKI. Images were taken after 10 days from seeding. The histogram reports the percentage of seeded cells that formed colonies. Statistical analysis was performed by using the Student's t-test (*p<0.05; **p<0.01). **L)** Enrichment score in the Drug signature database for the genes regulated by QKI at splicing level. **M)** Histograms displaying the analysis of cell sensitivity performed by crystal violet in HPAF-II cells expressing QKI-GFP treated or not with 2µM Irinotecan for 6 days. ***p<0.001 (Student's t-test) **N)** Images of HPAF-II cells transfected with GFP only (EV) or QKI-GFP. Scale bar=50µm. **O)** RT-PCR analysis of the QKI-regulated subtypespecific *ADD3*, *CTNND1*, *LRRFIP2*, *SYTL2* and *SEC16A* splice variants in HPAF-II cells transfected with QKI-GFP. **P)** RT-PCR analysis of the *LRRFIP2*, *SYTL2* and *SEC16A* splice variants in PANC-1 cells silenced or not for the corresponding basal like events. **Q)** Histograms showing the analysis of cell sensitivity performed by crystal violet in PANC-1 cells silenced for QKI. Treatments were carried out for 6 days with increasing concentration of gemcitabine (10nM, 15nM) or nab-paclitaxel (30nM, 50nM), or untreated (untr.). Results represent the mean ± SD of three experiments. Statistical analysis was performed by the Two-way ANOVA test (*p<0.05).
