# Supplementary material for: An alternative splicing signature defines the basal-like phenotype and predicts worse clinical outcome in pancreatic cancer
Source: Cell Rep Med. 2024 Feb 6;5(2):101411. doi: 10.1016/j.xcrm.2024.101411 (PMC10897606; doi:10.1016/j.xcrm.2024.101411)
Supplement: Document S2. Article plus supplemental information [file mmc9.pdf]

Cell Reports Medicine

Article

An alternative splicing signature defines the basal-like phenotype and predicts worse clinical outcome in pancreatic cancer

Graphical abstract

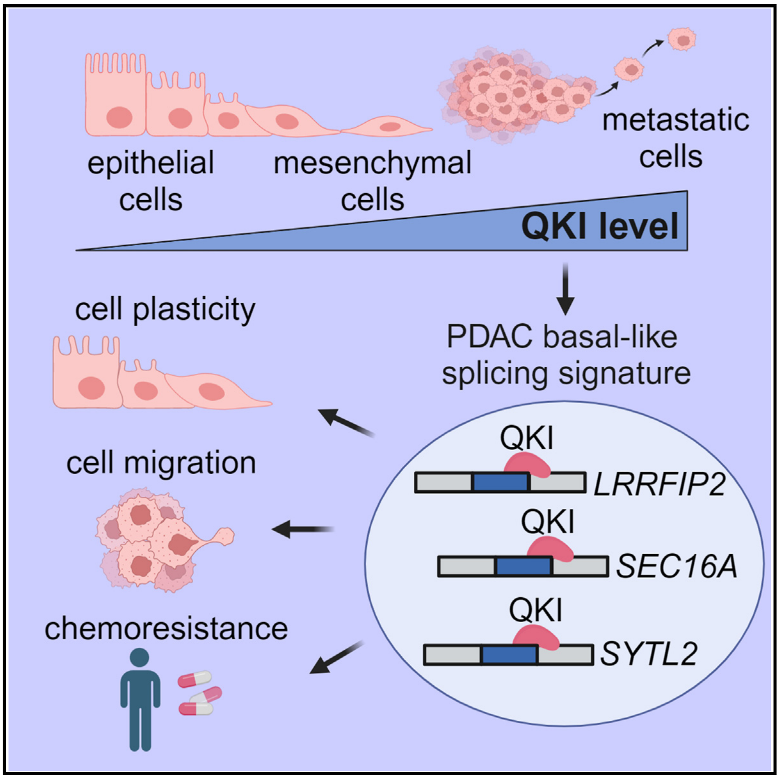

Highlights

- A splicing signature distinguishes the classical and basal-like PDAC subtypes
- The splicing factor QKI establishes the basal-like splicing signature
- QKI promotes a quasi-mesenchymal, plastic phenotype in PDAC cells
- The QKI-dependent splicing signature promotes resistance to mFOLFIRINOX in PDAC cells

Authors

Veronica Ruta, Chiara Naro, Marco Pieraccioli, ..., Alexander Kleger, Gabriele Capurso, Claudio Sette

Correspondence

claudio.sette@unicatt.it

In brief

Ruta et al. uncover a splicing signature that identifies molecularly and clinically distinct PDAC phenotypes, highlighting the splicing factor QKI as a marker of the basal-like subtype. They further show that QKI establishes a quasi-mesenchymal splicing pattern contributing to chemoresistance and adaptability to environmental changes in PDAC cells and organoids.

Ruta et al., 2024, Cell Reports Medicine *5*, 101411
February 20, 2024 © 2024 The Author(s).
https://doi.org/10.1016/j.xcrm.2024.101411

CellPress

Cell Reports Medicine

CellPress
OPEN ACCESS

Article

An alternative splicing signature defines the basal-like phenotype and predicts worse clinical outcome in pancreatic cancer

Veronica Ruta,[1] Chiara Naro,[1,2] Marco Pieraccioli,[1,2] Adriana Leccese,[1] Livia Archibugi,[3] Eleonora Cesari,[2] Valentina Panzeri,[1] Chantal Allgöwer,[4] Paolo Giorgio Arcidiacono,[3,5] Massimo Falconi,[5,6] Carmine Carbone,[2] Giampaolo Tortora,[2,7] Federica Borrelli,[2] Fabia Attili,[2] Cristiano Spada,[2] Giuseppe Quero,[2,8] Sergio Alfieri,[2,8] Claudio Doglioni,[5,9] Alexander Kleger,[4,10] Gabriele Capurso,[3,5] and Claudio Sette[1,2,11,*]

[1]Department of Neuroscience, Section of Human Anatomy, Catholic University of the Sacred Heart, 00168 Rome, Italy
[2]Fondazione Policlinico A. Gemelli IRCCS, 00168 Rome, Italy
[3]Pancreato-Biliary Endoscopy and Endosonography Division, Pancreas Translational and Clinical Research Center, San Raffaele Scientific Institute IRCCS, 20132 Milan, Italy
[4]Institute for Molecular Oncology and Stem Cell Biology, Ulm University Hospital, 89081 Ulm, Germany
[5]Vita-Salute San Raffaele University, 20132 Milan, Italy
[6]Pancreas and Transplantation Surgical Division, Pancreas Translational and Clinical Research Center, San Raffaele Scientific Institute IRCCS, 20132 Milan, Italy
[7]Medical Oncology, Catholic University of the Sacred Heart, 00168 Rome, Italy
[8]Gemelli Pancreatic Advanced Research Center (CRMPG), Catholic University of the Sacred Heart, 00168 Rome, Italy
[9]Division of Pathology, Pancreas Translational and Clinical Research Center, San Raffaele Scientific Institute IRCCS, 20132 Milan, Italy
[10]Division of Interdisciplinary Pancreatology, Department of Internal Medicine I, Ulm University Hospital, 89081 Ulm, Germany
[11]Lead contact
*Correspondence: claudio.sette@unicatt.it
https://doi.org/10.1016/j.xcrm.2024.101411

SUMMARY

Pancreatic ductal adenocarcinoma (PDAC) is characterized by extremely poor prognosis. PDAC presents with molecularly distinct subtypes, with the basal-like one being associated with enhanced chemoresistance. Splicing dysregulation contributes to PDAC; however, its involvement in subtype specification remains elusive. Herein, we uncover a subtype-specific splicing signature associated with prognosis in PDAC and the splicing factor *Quaking* (QKI) as a determinant of the basal-like signature. Single-cell sequencing analyses highlight QKI as a marker of the basal-like phenotype. QKI represses splicing events associated with the classical subtype while promoting basal-like events associated with shorter survival. QKI favors a plastic, quasi-mesenchymal phenotype that supports migration and chemoresistance in PDAC organoids and cell lines, and its expression is elevated in high-grade primary tumors and metastatic lesions. These studies identify a splicing signature that defines PDAC subtypes and indicate that QKI promotes an undifferentiated, plastic phenotype, which renders PDAC cells chemoresistant and adaptable to environmental changes.

INTRODUCTION

Pancreatic ductal adenocarcinoma (PDAC) is a lethal cancer, with 5-year survival of $\simeq$11%. Surgery represents the most efficacious therapy for PDAC. However, only a minority of patients are eligible for it.[1,2] Notably, unresectable PDACs with similar radiologic stage and histopathological features can display either complete unresponsiveness or partial response to chemotherapy, which might allow subsequent surgical intervention.[3] Thus, identification of molecular features that distinguish tumors with different clinical course is of paramount importance to improve management of patients with PDAC and particularly of unresectable cases that face different clinical options.

An important advancement in the field was the identification of two main molecular PDAC subtypes named classical and basal-like.[4,5] Classical PDACs express transcription factors involved in pancreas development, such as GATA6, and show improved response to mFOLFIRINOX (modified 5′-fluorouracil, leucovorin, irinotecan, oxaliplatin [mFOL]) treatment and a relatively better prognosis. By contrast, basal-like PDACs express markers of epithelial-to-mesenchymal transition (EMT) and are associated with worse chemotherapy response and prognosis.[4,5] More recently, single-cell transcriptomic analyses have indicated that most PDACs comprise cells of both molecular phenotypes, as well as intermediate cells that concomitantly express classical and basal-like features.[6–8] Clinical trials are currently assessing whether the knowledge achieved from transcriptome analyses of subtypes can effectively improve management of patients and constructively provide chemotherapeutic options.[5] Nevertheless, these high-throughput analyses are time consuming

This is an open access article under the CC BY license (http://creativecommons.org/licenses/by/4.0/).

and costly, limiting the possibility of integrating them into clinical practice. Since the percentage of cells with a basal-like signature is correlated with the severity of prognosis,[9] identification of reliable markers of this subtype represents a priority for PDAC management. Indeed, while GATA6 level was reported as a valuable prognostic marker to identify patients with classical features who respond to mFOL,[10] similar markers for the basal-like subtype are lacking.

Splicing dysregulation is a key feature of human cancers and contributes to tumorigenesis.[11,12] Alternative exon splicing in pre-mRNA allows most human genes to encode for multiple proteins that can exhibit different functions.[13,14] Tumors often express unique and oncogenic splice variants,[15] which can be exploited as therapeutic targets[11,12] or might generate valuable neoepitopes for immunotherapy.[16,17] Furthermore, splicing signatures efficiently discriminate tumor subtypes and can help stratify patients with stronger prognostic and diagnostic power than canonical gene expression signatures.[18] Importantly, the mechanisms underlying splicing dysregulation represent a vulnerability that can be exploited by targeting the splicing machinery.[11,12] In this regard, PDAC-associated mutations were reported to synergize in tumorigenesis by globally altering the splicing program of the cell.[19] Moreover, splicing factors were recently shown to either promote the early events in pancreatic tumorigenesis[20] and resistance to chemotherapy[21] or to limit the metastatic potential of PDAC cells.[22] At the same time, pharmacologic inhibition of splicing negatively impacted PDAC progression.[19,23] Nevertheless, it is unknown whether PDAC subtypes express different splicing signatures and if splicing regulators or splice variants feature a powerful diagnostic and/or prognostic potential.

This study identifies a splicing signature that distinguishes PDAC subtypes. Subtype-specific splice variants are accurate survival predictors when considered in the total population of patients with PDAC, as well as within subtype homogeneous cohorts, indicating their power as biomarkers. Furthermore, we uncover the role of the splicing factor *Quaking* (QKI) as a determinant of the basal-like splicing signature. QKI controls a pro-mesenchymal splicing program associated with migration and mFOL resistance in basal-like cells. Importantly, high QKI expression correlates with metastatic stage and poor outcome in PDAC. These findings reveal that QKI is a reliable basal-like marker and defines a subtype-specific splicing signature with prognostic potential in PDAC, which may allow more precise stratification of patients for the selection of the available clinical options.

RESULTS

A splicing signature distinguishing PDAC subtypes

To identify splicing signatures associated with PDAC molecular subtypes, we analyzed the transcriptome from TCGA samples selected for homogeneous representation of tumor cells.[24] Samples were classified as classical (n = 45) or basal-like (n = 32) according to their gene expression signatures.[25,26] Basal-like tumors were associated with worse prognosis with respect to classical tumors (Figure S1A). By using the *psichomics* tool,[27] we found 184 genes with up-regulated expression in basal-like tumors and 342 genes with predominant expression in classical samples (fold change $\geq$ 2; $p < 0.05$; Figure S1B; Table S1). In line with their worse prognosis, genes up-regulated in the basal-like samples were enriched in functional categories related to PDAC stemness, metastatic and aggressive behaviors,[28] like the WNT pathway, extracellular matrix organization, and migration (Figure S1C). Next, splice junction analysis highlighted 35 splicing events differentially regulated between classical and basal-like samples (Table S1), with exon cassette (EC) being the most represented pattern (71.4%; Figures 1A and 1B). Annotation of the splicing-regulated genes indicated a relationship with cancer-relevant processes,[29] such as cell adhesion, signal transduction, and metabolism (Figures 1C and 1D), suggesting the potential impact of these splicing switches on the PDAC phenotype.

Subtype-specific splicing events segregate patients with PDAC that display different clinical courses

Given the prominent representation of EC events (Figure 1A), we interrogated this pattern in further analyses. Visual representation of the "percent of spliced in" (PSI) of six representative subtype-specific ECs illustrates the increased inclusion of "classical" exons (in *SEC31A*, *CTNND1*, and *ADD3* genes) in classical tumors and of "basal-like" exons (in *LRRFIP2*, *SEC16A*, and *SYTL2* genes) in basal-like tumors (Figures 1E and 1F). Inclusion of 13 subtype-specific ECs displayed a prognostic value upon examination in the entire TCGA cohort (n = 77). For instance, higher inclusion of individual classical ECs was sufficient to significantly segregate patients with longer relapse-free survival (Figures 1G and S1D), whereas inclusion of basal-like ECs was associated with worse prognosis (Figures 1H and S1E). Noteworthy, inclusion of the *ADAM15* and *SEC16A* EC events predicted better or worse prognosis, respectively, even within homogeneous classical or basal-like patients (Figures S1F and S1G). These results suggest the high prognostic potential of the subtype-specific splicing signature.

TCGA transcriptomic data refer to resected tumor samples. However, only a minority (<30%) of patients with PDAC are eligible for upfront surgery, due to the presence of advanced disease at diagnosis.[1] Thus, we sought to validate the prognostic value of subtype-specific splicing events in an independent cohort of patients that were not selected for disease stage. Samples were collected from 22 patients undergoing diagnostic biopsy by endoscopic ultrasound tissue acquisition (EUS-TA).[30] For these patients, the available follow-up data allowed direct comparison of molecular and clinical features (Table S2). Based on quantity and quality of the RNA, RNA sequencing (RNA-seq) analysis could be performed on 14 samples. By applying the PURIST algorithm,[26] only patient 78 was classified as basal-like, while the others were all classical tumors (Figures S1H and S1I). Interestingly, RT-PCR analysis of the *ADAM15* and *SEC16A* ECs confirmed their prognostic value also in this smaller, unselected cohort. As predicted from TCGA data analysis, inclusion of *ADAM15* exon 22 was correlated with survival and better prognosis, regardless of the subtype (Figures 2A–2C), whereas inclusion of the basal-like EC in *SEC16A* significantly correlated with worse overall survival (Figures 2D–2F). Notably, the basal-like patient (#78) displayed the lowest PSI for *ADAM15* exon 22 (Figure 2B) and one of the highest for *SEC16A* exon 33 (Figure 2E). These observations indicate that

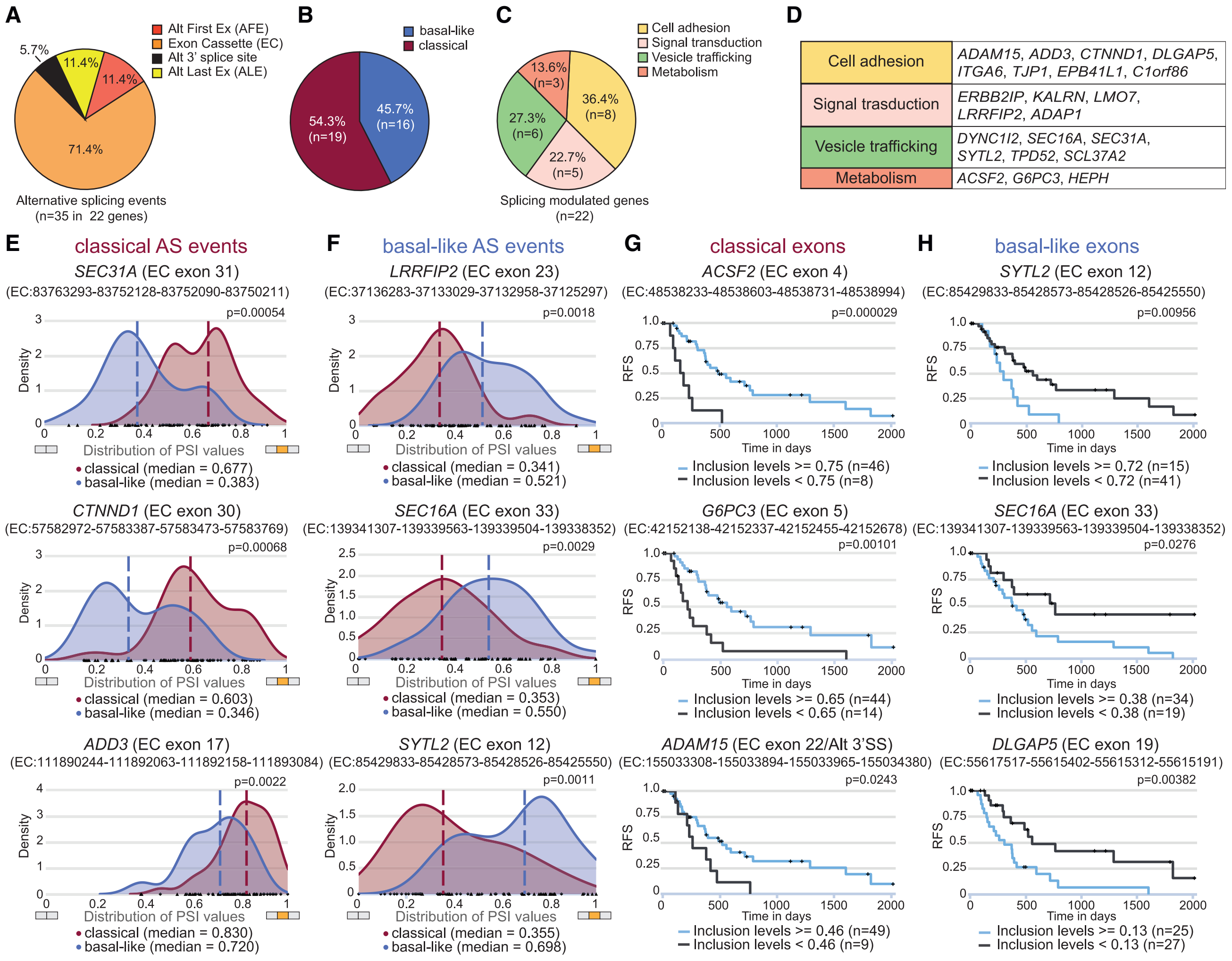

Figure 1. Identification of the PDAC subtype-specific splicing signature

(A–C) Pie charts of splicing patterns differentially regulated in classical vs. basal-like PDAC subtypes (A), splicing events up-regulated in classical or basal-like tumors (B), and biological processes of splicing-regulated genes (C).

(D) List of the splicing-regulated genes.

(E and F) PSI distribution of "classical" exons in the *SEC31A*, *CTNND1*, and *ADD3* genes and of "basal-like" exons in the *LRRFIP2*, *SEC16A*, and *SYTL2* genes in TCGA PDAC samples. The regulated EC is shown in orange in the x axis. Statistical analyses of splicing regulation (|Δ median PSI| ≥ 0.1 and false discovery rate [FDR] ≤ 0.01) was performed by the Wilcoxon rank-sum test with Benjamini-Hochberg (FDR) adjustment.

(G and H) Kaplan-Meier curves of relapse-free survival (RFS) of patients with PDAC segregated for inclusion (blue line) or skipping (black line) of classical (G) and basal-like (H) exons. Type of event, regulated exon(s), and genomic coordinates of the regulated exon(s) and of the flanking splice sites are indicated.

See also Figure S1 and Table S1.

the splicing signature allows for reliable identification of molecularly and clinically distinct PDAC subtypes.

Select RNA-binding proteins determine the PDAC subtype-specific splicing signature

Alternative splicing is modulated by sequence-specific RNA-binding proteins (RBPs) that act in a cell- and tissue-specific fashion.[13,31,32] To identify RBPs that establish the subtype-specific splicing signature, we interrogated the relation between their expression and the inclusion of differentially regulated exons in TCGA PDAC samples. Eight RBPs significantly correlated with ≥30% of the subtype-specific splicing events (Figure 3A; Table S3). RBM47, RAVER2, A1CF, and ESRP2 positively correlated with GATA6 and were expressed at significantly higher level in classical tumors (Figures 3B, S2A, and S2C), whereas RBMS1/2, QKI, and RBFOX2 were inversely correlated with GATA6 and were expressed at higher levels in basal-like tumors (Figures 3B, S2B, and S2D).

To assess whether these RBPs are involved in the regulation of the identified splicing signature, we employed PDAC cell lines that are representative of classical and basal-like tumors.[33,34] Coherently with our computational analysis in PDAC samples, classical cells (HPAF-II, Capan-1) showed higher inclusion of classical exons, whereas basal-like cells (PANC-1, MiaPaCa-2)

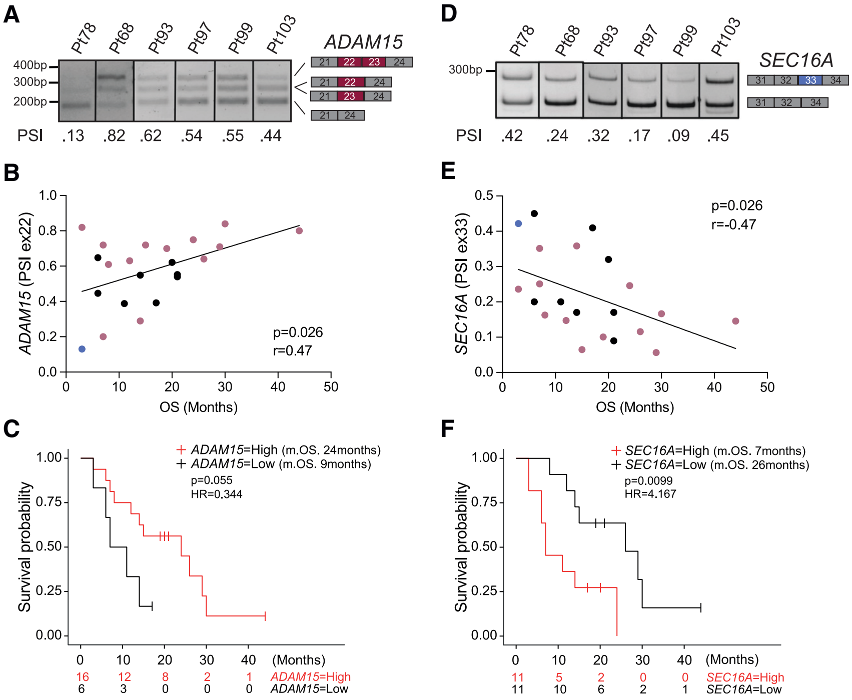

Figure 2. Inclusion of the ADAM15 and SEC16A ECs is associated with prognosis in unselected patients with PDAC

(A) Representative splicing assays of the *ADAM15* EC in PDAC samples.

(B) Pearson's correlation analysis between the *ADAM15* exon 22 PSI and overall survival (OS) in unselected patients with PDAC (n = 22). The blue and pink dots correspond to, respectively, basal-like and classical samples identified by RNA-seq (Figure S2). Black dots correspond to non-sequenced samples.

(C) Kaplan-Meier curve displaying the OS of patients with PDAC exhibiting high (red) or low (black) inclusion levels of *ADAM15* exon 22 (m.s., median survival).

(D) Representative splicing assays of the *SEC16A* EC in PDAC samples.

(E) Pearson's correlation analysis between the PSI of *SEC16A* exon 33 and OS in unselected patients with PDAC (n = 22).

(F) Kaplan-Meier curve displaying OS of patients with PDAC exhibiting high (red) or low (black) inclusion levels of *SEC16A* exon 33.

See also Figure S1 and Table S2.

displayed increased inclusion of basal-like exons (Figures S3A and S3B). Quantitative real-time PCR (real-time qPCR) analyses indicated that Capan-1 cells express high levels of most of these RBPs, whereas the other cell lines were more representative of what was observed in patients with PDAC (Figure S3C). RBM47 was significantly more expressed in HPAF-II cells than in basal-like cells, while ESRP2 was similarly expressed in HPAF-II and PANC-1 cells but was not expressed in MiaPaCa-2 cells (Figure 3C). By contrast, RAVER2, which associates with the classical subtype in patients, was more expressed in basal-like cells (Figure 3C). A1CF expression was not detected. On the other hand, with the exception of RBMS2, all basal-like RBPs were expressed at higher level in PANC-1 and MiaPaCa-2 cells with respect to HPAF-II cells (Figure 3C).

Knockdown of RBM47 in HPAF-II cells was sufficient to repress the inclusion of classical ECs, whereas depletion of either RAVER2 or ESRP2 was ineffective (Figures 3D and S3D). Depletion of QKI and, to a lesser extent, RBFOX2 caused significant skipping of the basal-like ECs in both basal-like cell lines (Figures 3E and S3E–S3G). Moreover, we confirmed a higher expression of QKI protein in basal-like cells and of RBM47 in HPAF-II cells. Two RBFOX2 protein isoforms (p55 and p70) were detected in all cell lines, with expression of p55 being slightly up-regulated in PANC-1 and that of p70 in MiaPaCa-2 cells (Figure S4A). These results indicate that RBM47, QKI, and, possibly, RBFOX2 contribute to the establishment of the PDAC subtype-specific splicing signature.

QKI and RBFOX2 orchestrate a widespread splicing program in basal-like PDAC cells

The basal-like subtype features sturdy malignancy, resistance to chemotherapy, and poor clinical outcome.[9,10,26] Thus, we sought to address the potential role of QKI and RBFOX2 as determinants of the basal-like phenotype. These RBPs play a role in both nuclear and cytoplasmic RNA processing events.[35,36] However, basal-like cells prevalently express the nuclear QKI-5 isoform, which is involved in splicing regulation, with respect to the cytoplasmic isoforms QKI-6/7[35] (Figures S4B and S4C). RBFOX2 was also prevalently nuclear in basal-like cells (Figure S4C). Since PANC-1 cells displayed higher expression levels of QKI and RBFOX2 with respect to MiaPaCa-2 cells (Figure 3C), we employed this cell line for further studies. RNA-seq analyses showed that QKI knockdown affected splicing of 4% of the genes expressed in PANC-1 cells (665 events in 499 genes), while RBFOX2 silencing was associated with 413 splicing events in 348 genes (3% of expressed genes; Figures 4A–4C; Tables S4). Knockdown of both RBPs mainly impacted ECs, which represented ~50% of all regulated events (Figure 4C). We found a large (n = 98) and highly significant ($p = 2.8e{-}40$) overlap between the splicing events regulated by QKI and RBFOX2 (Figure 4D). Moreover, all common events were regulated in the same direction upon silencing of either RBP (Figure 4E), as was also observed for the basal-like ECs (Figure 3E).

RBPs regulate splicing by directly binding to target exons or their flanking introns.[13,31] An unbiased search for the most enriched sequence elements identified the (A/T/G)CTAAC motif in proximity to the QKI-regulated exons (exon ± 250 bp) with respect to constitutive exons (Figure S5A) and non-regulated (reference) ECs (Figure 4F). Computational analyses confirmed that this motif is highly homologous to the QKI binding site (Figure 4F).[37] Likewise, the RBFOX motif TGCATG[38] was significantly enriched in RBFOX2-regulated ECs with respect to both constitutive exons (Figure S5B) and reference ECs (Figure 4F). The proportion of these motifs was increased in proximity to exons regulated by these RBPs (Figures 4G and 4H). In line with their positional effect on splicing regulation,[39,40] both motifs

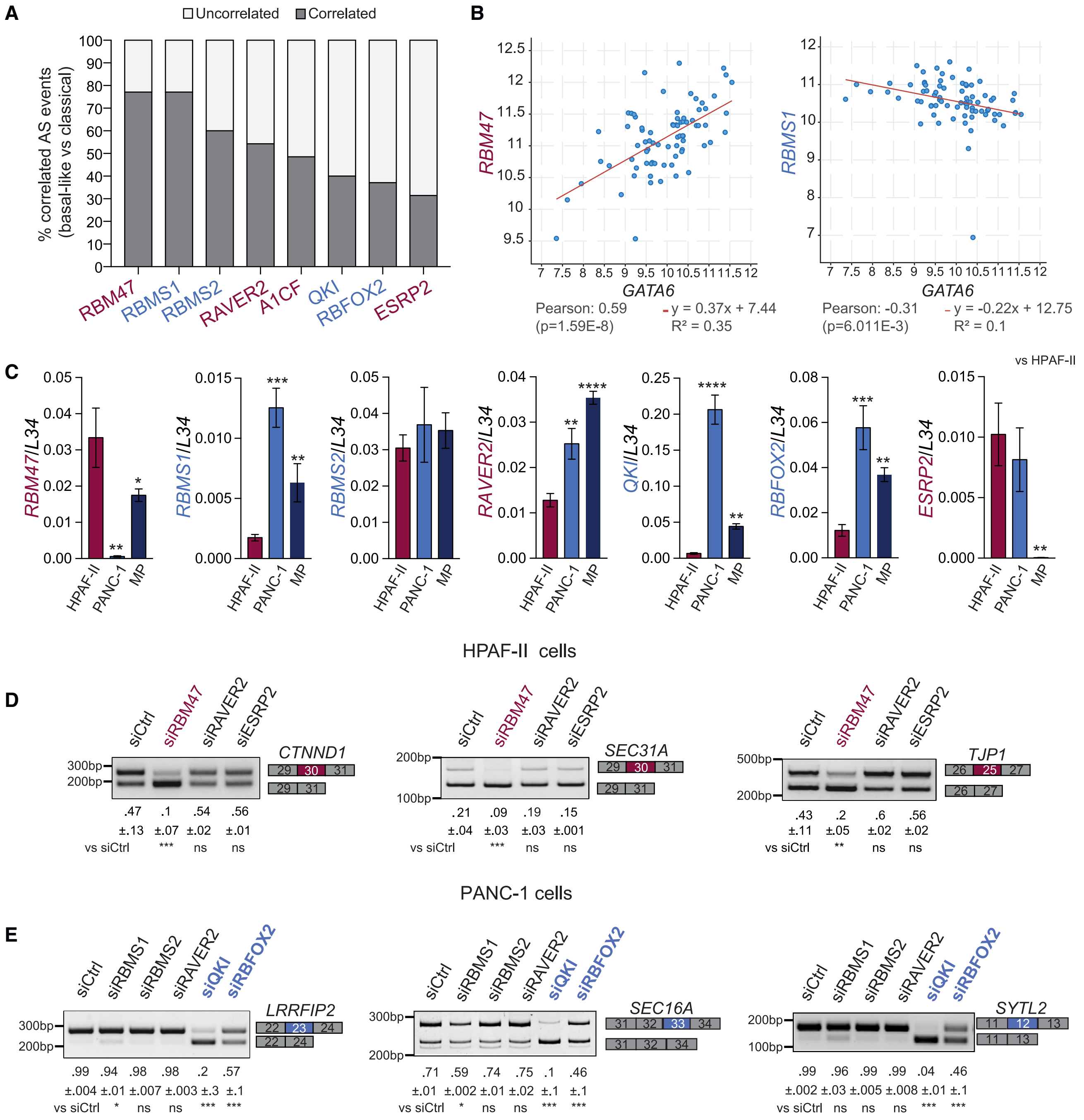

Figure 3. Identification of RBPs involved in the establishment of the subtype-specific splicing signature

(A) Percentage of the subtype-specific splicing events that correlate to the indicated RBPs. RBPs expressed at higher levels in classical tumors are listed in red; those expressed at higher levels in basal-like tumors are listed in blue.
(B) Correlation analysis between the expression of GATA6 and that of RBM47 or RBMS1.
(C) Quantitative real-time PCR (real-time qPCR) analysis of RBP expression in PDAC cell lines.
(D and E) Representative splicing assay (n = 3) after silencing RBPs in HPAF-II (D) or PANC-1 (E) cells.
(C–E) Mean ± standard deviation (SD) of three independent experiments. Student's t test; ***p = 0.001.
See also Figures S2–S4 and Table S3.

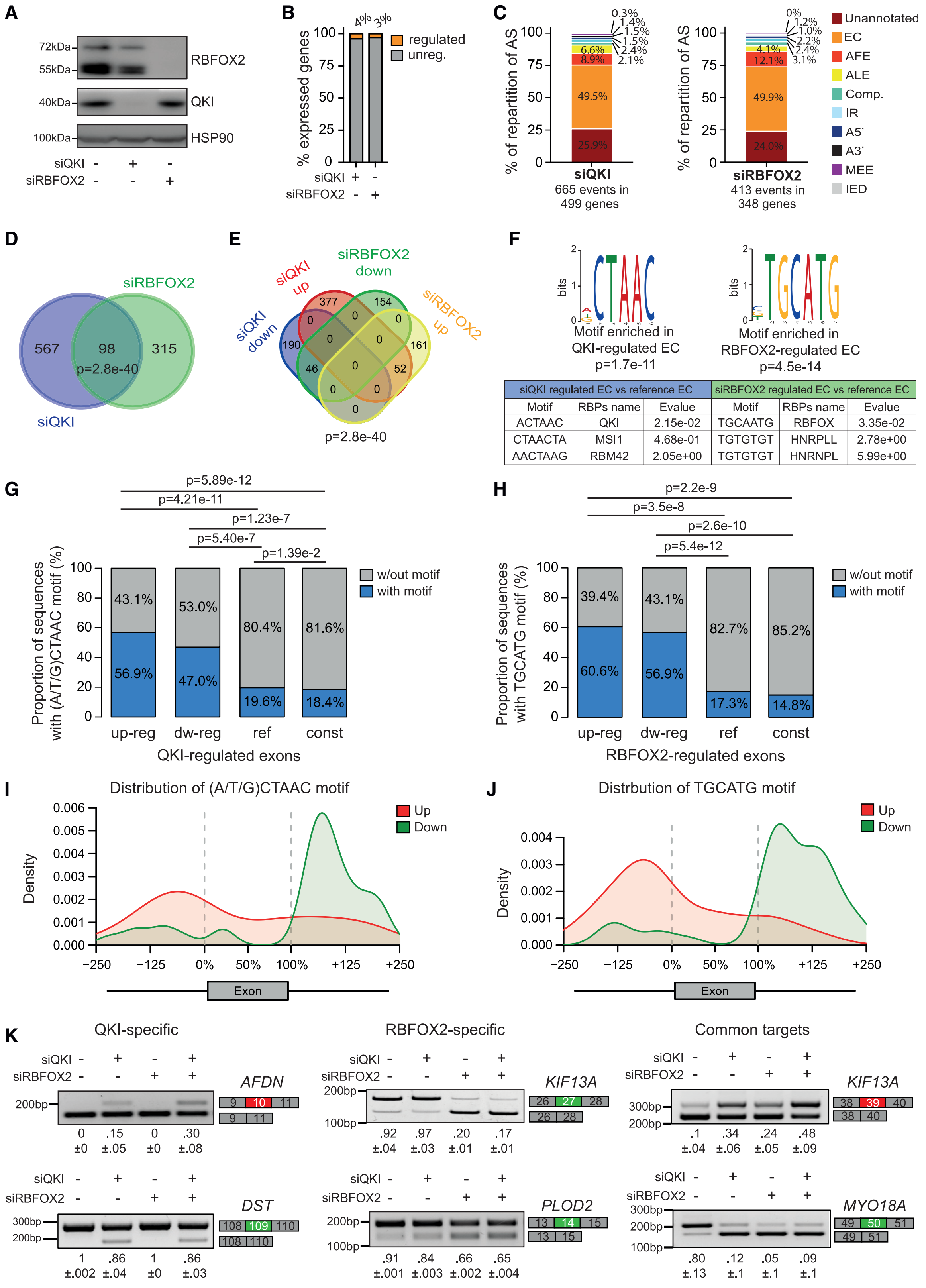

siQKI regulated EC vs reference EC			siRBFOX2 regulated EC vs reference EC		
Motif	RBPs name	Evalue	Motif	RBPs name	Evalue
ACTAAC	QKI	2.15e-02	TGCAATG	RBFOX	3.35e-02
CTAACTA	MSI1	4.68e-01	TGTGTGT	HNRPLL	2.78e+00
AACTAAG	RBM42	2.05e+00	TGTGTGT	HNRNPL	5.99e+00

(legend on next page)

were enriched upstream or within the repressed exons (i.e., up-regulated upon RBP knockdown) and downstream of exons whose splicing was induced by them (i.e., down-regulated upon RBP knockdown) (Figures 4I and 4J). RT-PCR analysis of selected genes validated the reliability of the bioinformatics analyses, confirming the specificity of QKI- (*AFDN* exon 10, *DST* exon 109) and RBFOX2-regulated (*KIF13* exon 27, *PLOD2* exon 14) events (Figure 4K), as well as the effect of both RBPs on events that were predicted to be common targets (Figure 4K).

QKI is the main regulator of the basal-like splicing signature

QKI depletion was sufficient to modulate 32% of the subtype-specific splicing events that occur in PANC-1 cells, whereas RBFOX2 knockdown exerted a more limited impact (17.8% of events). Moreover, all the RBFOX2-regulated exons were also influenced by QKI (Figure 5A; Table S4). In line with this latter observation, the RBFOX binding motif was significantly enriched among the QKI-regulated ECs (Figure S5A). Furthermore, as exemplified by the basal-like *LRRFIP2*, *SEC16A*, and *SYTL2* ECs, depletion of QKI exerted a stronger effect than RBFOX2, while their combined knockdown only mildly increased the effect of QKI depletion (Figures 5B and S5C–S5E). To further dissect the contribution of these RBPs to the basal-like signature, we focused on *SEC16A* exon 33, which associates with poor prognosis in patients with PDAC (Figures 1H and 2F). Inspection of the sequences flanking the EC highlighted two putative binding sites for QKI (ACTAAC) located 22 and 95 bp downstream of the 5′ splice site, respectively (Figure 5C). We also identified a putative binding site for RBFOX2 (TGCATG) 44 bp upstream of the 3′ splice site and a less conserved (AGCACG) motif 60 bp downstream of the 5′ splice site (Figure 5C). Data from crosslink immunoprecipitation (CLIP)-seq experiments confirmed binding of QKI to both putative binding sites and of RBFOX2 to the less conserved site 2 (Figure S5F). Accordingly, CLIP assays in PANC-1 cells confirmed binding of QKI and RBFOX2 to the proximal part of intron 33 (Figure S5G).

To investigate the relative contribution of these binding sites to splicing regulation, we constructed a minigene encompassing the *SEC16A* genomic region from exons 32 to 34 (Figure 5D). Transfection of the *SEC16A* minigene in PANC-1 cells recapitulated the splicing pattern of the endogenous gene, with exon 33 being preferentially included (Figure 5D). QKI depletion strongly repressed exon 33 inclusion, while knockdown of RBFOX2 exerted a much milder effect, and combined depletion of the two RBPs caused almost complete skipping of the exon (Figure 5D). Moreover, mutation of the proximal QKI binding site in intron 33 (site 1) completely abolished exon 33 inclusion, whereas mutation of distal site 2 or of the two RBFOX2 binding sites exerted, respectively, milder or no effects (Figures 5E and 5F). These results support the prominent role played by QKI in the establishment of the basal-like splicing signature.

QKI represses the classical splicing signature and associates with the basal-like phenotype

Most of the subtype-specific exons regulated by QKI were classified as classical in our analysis (Figure 6A; Table S4). As exemplified by the *ADD3* and *CTNND1* ECs, QKI repressed these classical exons in basal-like cells, and its depletion restored the splicing pattern observed in classical cells (Figure 6B). Although the *ADD3* EC was predicted to be also a target of RBFOX2 (Table S4), its repressive effect was weaker than that of QKI (Figure 6B).

To address whether QKI globally impacted on the subtype identity of PDAC cells, we compared the transcriptome of HPAF-II and PANC-1 cells. We found a highly significant ($n = 112$; $p = 2.61e{-34}$) overlap between the ECs differentially regulated in these two cells lines (Table S5) and those regulated by QKI (Figures 6C; Table S4). QKI depletion promoted the classical pattern for most of these common EC events (>90%; Figure 6D). RT-PCR analysis confirmed that the *ESYT2*, *RAI14*, and *EXOC1* ECs were more included in classical cells with respect to basal-like cells. Furthermore, QKI depletion in basal-like cells was sufficient to induce the classical splicing pattern (Figure 6E), supporting its key role in establishing the subtype-specific splicing signature. As shown for the *RAI14* and *EXOC1* ECs, higher inclusion of some QKI-repressed classical exons predicted better prognosis (Figures S5H and S5I), further highlighting the clinical relevance of the QKI-dependent splicing program.

Next, to test whether QKI represents a valuable marker of the basal-like identity, we analyzed single-cell transcriptomic data from 27 PDAC samples.[8] Clustering analyses of gene expression

Figure 4. Transcriptome analysis of PANC-1 cells depleted of QKI or RBFOX2

(A) Western blot analysis of QKI and RBOFOX2 in PANC-1 cells transfected with the corresponding small interfering RNAs (siRNAs) (48 h).

(B) QKI or RBFOX2 splicing-regulated genes represented as percentage (orange) of total genes expressed in PANC-1 cells.

(C) Percentage of the indicated splicing patterns regulated by depletion of either QKI or RBFOX2. EC, exon cassette; AFE, alternative first exon; ALE, alternative last exon; Comp, complex event; IR, intron retention; A5′, alternative 5′ splice site; A3′, alternative 3′ splice site; MEE, mutually exclusive exon; IED, internal exon deletion.

(D) Overlap between QKI- and RBFOX2-regulated events identified in PANC1 cells.

(E) Venn diagram showing the direction of the regulated events in QKI- or RBFOX2-depleted cells.

(D and E) Statistical analysis was performed by the hypergeometric test.

(F) Logo representation of the motif most significantly enriched in proximity of the regulated exons identified in (C). RBPs predicted to bind to the indicated sequences (Tomtom motif comparison tool) are shown below the logos.

(G and H) Bar graphs showing the percentage of QKI- or RBFOX2-regulated exons (up or down) containing the identified motifs in comparison with their abundance in reference non-regulated ECs (ref) or constitutive exons (const).

(I and J) Distribution of the indicated QKI (I) and RBFOX2 (J) motifs within exons and flanking introns (±250 nt) in the up-regulated (red) and down-regulated (green) exons.

(K) RT-PCR analyses for the indicated ECs: QKI-specific (left), RBFOX2-specific (middle), and common (right) targets were selected. Percentage of splicing inclusion ± SD ($n = 3$) of ECs was evaluated by densitometry.

See also Figures S4 and S5 and Table S4.

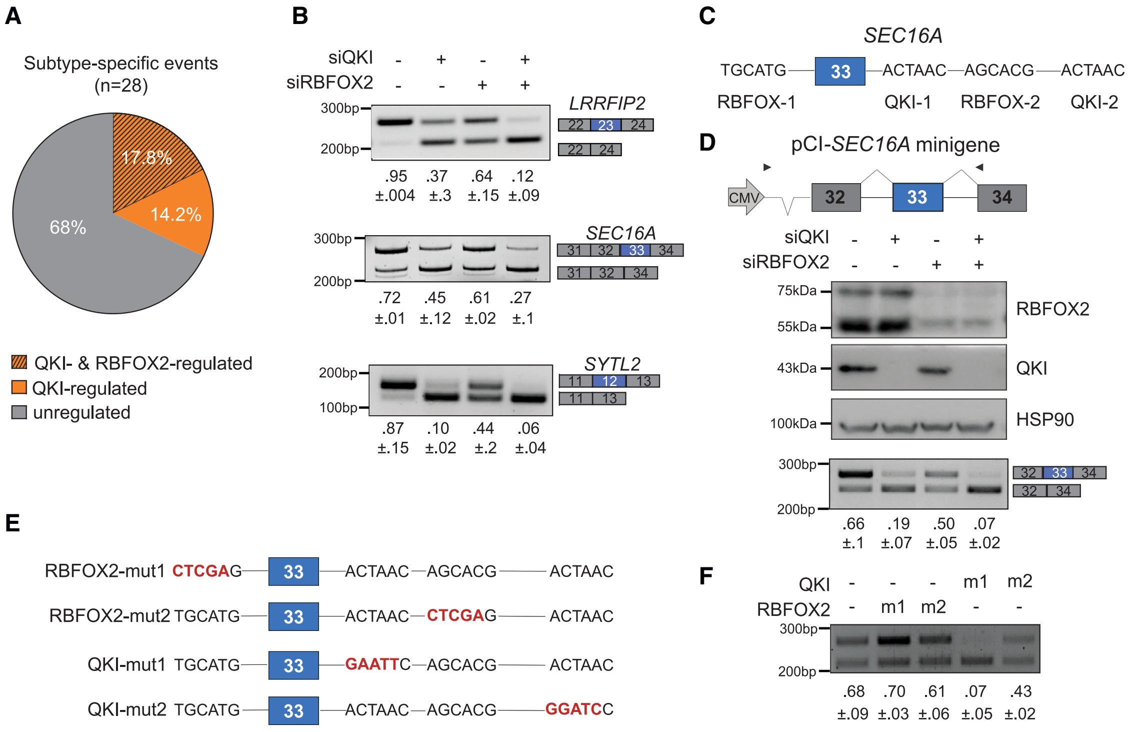

Figure 5. QKI regulates the basal-like splicing signature in PDAC cells

(A) Subtype-specific events expressed in PANC-1 cells and regulated by QKI and RBFOX2.

(B) Representative splicing assay of *LRRFIP2*, *SEC16A*, and *SYTL2* ECs in PANC-1 cells silenced for QKI, RBFOX2, or both. Mean ± SD (n = 3) is reported below the panels.

(C) Schematic representation of the QKI and RBFOX2 binding sites in the sequence flanking *SEC16A* exon 33.

(D) Schematic representation of the *SEC16A* minigene (top), western blot analysis, and splicing assay of the *SEC16A* minigene performed in PANC1-1 cells depleted of QKI, RBFOX2, or both.

(E) Mutations of the QKI and RBFOX2 binding sites performed in the *SEC16A* minigene.

(F) Splicing assay of QKI/RBFOX2 mutants in PANC-1 cells. Mean ± SD (n = 3) is reported below the panels.

See also Figure S5 and Table S4.

signatures indicated that most samples comprised cells of both classical and basal-like profiles at variable proportions (Figure S6A). In addition, the cumulative analysis of cells from all these tumors indicated a significantly higher level of QKI in basal-like cells (Figure 6F), while GATA6 displayed an inverse distribution (Figure S6B). These data confirm the extensive intratumor heterogeneity of PDAC and indicate that QKI expression is a reliable marker of the basal-like tumor cell population. Accordingly, tumor classification according to the Moffitt's signature[8] revealed that QKI expression increases in intermediate and basal-like PDAC, while GATA6 levels follow an opposite trend (Figures 6G and S6C–S6E). A similar inverse correlation was also observed by single-cell analysis of 24 samples from a different study (Figure S6F).[6] An amalgamation of all PDAC samples from these two independent studies (n = 51) indicated that QKI-positive (QKI^+) tumors were mostly classified as basal-like (37.5%) or intermediate (50%), whereas more than 55% of the QKI-negative (QKI^-) samples were defined as classical (Figure 6H). The potential of QKI expression levels in predicting the subtype was comparable to that of GATA6, as shown by a similar percentage of classical tumors among the $GATA6^+$ and QKI^- samples (Figure 6H). Furthermore, the combination of these two markers resulted in classifying >76% of $GATA6^+/QKI^-$ samples as classical, while $GATA6^-/QKI^+$ samples were either classified as intermediate or basal-like (Figure 6H). These analyses also indicate that tumors expressing QKI often display a heterogeneous signature, with 50% of QKI^+ and 41.7% of $GATA6^-/QKI^+$ samples being classified as intermediate between the classical and the basal-like phenotype (Figure 6H).

The transcriptional state of PDAC cells is highly plastic and influenced by environmental factors.[7] PDAC patient-derived organoids (PDOs) were shown to acquire a classical signature under culture conditions *in vitro*. However, upon switching to a minimal medium, PDOs were pushed to acquire intermediate or basal-like features.[7] To test whether the subtype-specific splicing signature was also influenced by microenvironmental changes, we employed two PDO lines derived from PDAC surgical resections (Figures 6I and S6G; Table S2). While both PDOs displayed a classical splicing signature, basal-like exons (*LRRFIP2*, *SEC16A*, and *SYTL2*) were partially included in PDO-9 (Figure S6H), suggesting an intermediate phenotype. Switching the culture to minimal medium promoted the basal-like splicing signature only in the intermediate PDO-9 line (Figures 6I and 6J). QKI expression was higher in PDO-9 with

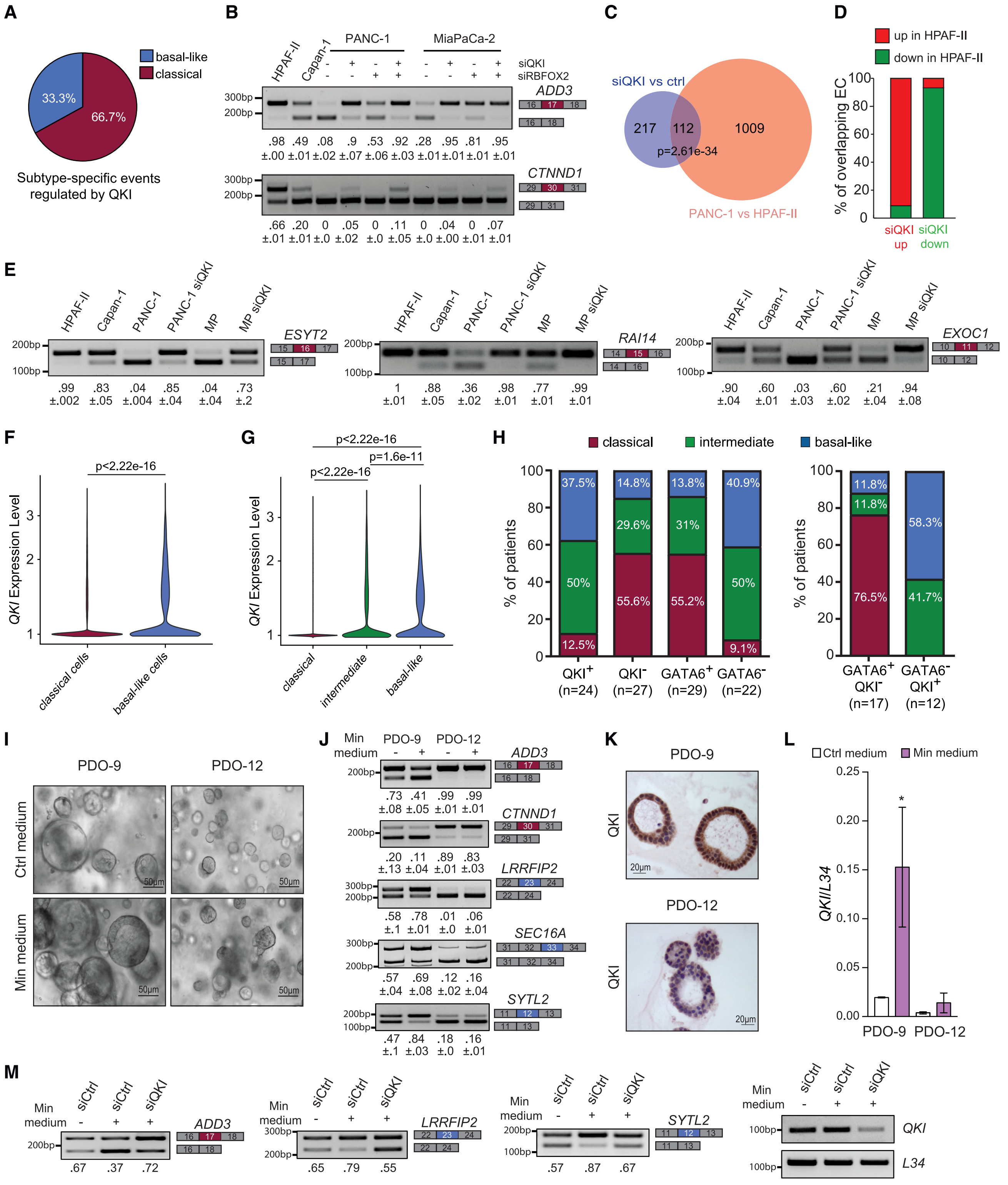

Figure 6. QKI expression is a marker of the basal-like identity of PDAC cells

(A) Pie chart of subtype-specific events regulated by QKI.

(B) Splicing assays of *ADD3* and *CTNND1* ECs in HPAF-II, Capan-1, and PANC-1 and MiaPaCa-2 (MP) cells silenced for QKI, RBFOX2, or both. Mean ± SD of the PSI value (n = 3) is reported below the panels.

(legend continued on next page)

respect to PDO-12 (Figure 6K), and culture in minimal medium further increased it (Figure 6L). At the same time, silencing of QKI suppressed this environmental-induced splicing switch (Figures 6M and S6I), further indicating that QKI contributes to the plastic, basal-like phenotype of PDAC cells.

QKI promotes a pro-mesenchymal phenotype in PDAC cells and correlates with poor survival

Cellular plasticity is a hallmark of cancer that confers increased oncogenic potential and is associated with metastatic dissemination and resistance to chemotherapy. Plasticity is characterized by the ability of cells to undergo EMT and to acquire stem-like features.[29,41] Basal-like PDAC cells display a quasi-mesenchymal phenotype, which translates to an aggressive and metastatic behavior.[7,42] Thus, we first tested whether the basal-like splicing signature regulated by QKI was associated with EMT. Despite the different tissues of origin, we observed a significant overlap between QKI-regulated ECs and exons that are modulated during EMT in breast tumors[43] (Figure 7A). QKI depletion in PANC-1 cells switched the splicing pattern toward that of epithelial/classical cells for most of these ECs (86.3%; Figures 7B and 7C; Table S6). Accordingly, QKI expression in TCGA PDAC samples was highly correlated with well-known EMT inducers, like the transcription factors ZEB1 and SNAI1, and inversely correlated with that of epithelial genes (*CDH1* and *ESRP2*; Figures S7A and S7B). Moreover, transient (Figure 7D) or stable (Figure S7C) knockdown of QKI negatively impacted PANC-1 cell migration, without affecting their proliferation (Figure S7E). On the other hand, forced expression of QKI in HPAF-II cells significantly enhanced their migratory activity (Figure S7D). QKI-regulated genes are enriched in functional terms related to adhesion (Figure S7F), and QKI knockdown promoted cell adhesion and expression of epithelial markers (Figures S7G and S7H) while repressing EMT-associated factors (Figure S7I).

Mesenchymal features in PDAC cells are associated with increased malignancy.[42] Given the association of QKI-dependent splicing events with poor prognosis in patients with PDAC, we asked whether QKI expression was also a prognostic factor. Elevated QKI expression was associated with shorter overall survival of TCGA patients (Figure S7J) and in our independent cohort that was not classified according to disease stage (Figures 7E; Table S2). Immunohistochemical (IHC) analyses of an additional 44 samples from patients subjected to surgical resection without prior chemotherapy, yet who were similar in demographics and other pathological features (Table S2), further indicated that QKI is associated with malignancy, being more expressed in poorly differentiated (G3) PDAC with respect to moderately/well differentiated (G1 or G2) tumors (Figures 7F and 7G). Nuclear QKI expression was detected in the majority of neoplastic cells in G3 PDAC and in the G3 components of tumors with heterogeneous grade composition (Figures 7F; Table S2). By contrast, most neoplastic cells of G1 and G2 PDAC were negative for QKI (Figures 7F; Table S2). QKI immunoreactivity was also observed in macrophages, regardless of the grade of the lesion (Figure 7F, left; Table S2), and in centro-acinar cells of non-tumoral pancreatic tissue (Table S2). Epithelial cells lining the small ducts, acinar cells, and glandular cells of large ducts, as well as pancreatic intraepithelial neoplasm (PanIN) and intraductal papillary mucinous neoplasm (IPMN) featuring different degrees of dysplasia, were all negative for QKI (Table S2).

Coherently with a role in tumor progression, analysis of single-cell RNA-seq data[8] indicated that QKI expression increased in cells of borderline resectable, locally advanced, and metastatic PDAC (Figure 7H). To assess whether QKI expression was also associated with progression to metastatic stage in individual patients, we analyzed samples from matched primary lesions and liver metastases of patients who underwent EUS-TA for diagnostic purposes. In all cases examined (n = 9), QKI was more expressed in the metastatic lesion with respect to the primary tumor (Figure 7I). Moreover, QKI knockdown reduced the clonogenicity in PANC-1 cells (Figure 7J), a feature associated with metastatic colonization by cancer cells,[44] whereas its up-regulation in HPAF-II increased clonogenicity (Figure S7K). These findings further suggest a direct correlation between QKI expression and advanced disease stages.

Negative prognosis in PDAC is also related to resistance to chemotherapy. Classical tumors were reported to respond better than basal-like tumors to the mFOL treatment.[10] By querying the Drug Signature Database, which reports data from drug-induced expression changes,[45] we observed that QKI-regulated genes are enriched for sensitivity to irinotecan (Figure S7L), a

(C) Overlap between events regulated by QKI and those regulated in PANC-1 vs. HPAF-II. Statistical analysis was performed by the hypergeometric test.
(D) Histograms representing the splicing events of the overlap in (C) and showing the type of regulation (up or down) in HPAF-II cells with respect to PANC-1 cells. Events were separated between those up- or down-regulated in QKI-depleted PANC-1 cells.
(E) Splicing assays of *ESYT2*, *RAI14*, and *EXOC1* ECs in HPAF-II, Capan-1, PANC-1, and MP cells. The latter two cell types were also depleted or not for QKI. Mean ± SD of the PSI value (n = 3) is reported below the panels.
(F) Violin plot of QKI expression level in classical and basal-like cells identified by single-cell transcriptomic analysis.
(G) Violin plot showing of QKI expression in tumors classified as classical, intermediate, and basal-like by single-cell transcriptomic analysis.
(F and G) Statistical analyses were performed by Wilcoxon's test.
(H) Histogram showing percentage of classical/intermediate/basal-like patients that were evaluated as positive or negative for QKI ($QKI^{+/-}$) or GATA6 ($GATA6^{+/-}$) expression (left) or classified as $GATA6^{+}/QKI^{-}$ or $GATA6^{-}/QKI^{+}$ (right).
(I and J) Representative images of PDAC PDO-9 and PDO-12 lines cultured for 9 days in regular or minimal medium (I) and corresponding splicing assay of the *ADD3*, *CTNND1*, *LRRFIP2*, *SYTL2*, and *SEC16A* ECs (J). The PSI value is reported below the panels. Scale bar: 50 μm.
(K) Immunohistochemistry (IHC) analysis of QKI in PDO-9 and PDO-12. Scale bar: 20 μm.
(L) RT-qPCR analysis of QKI expression in PDOs after 9 days in minimal media.
(M) Splicing assay of indicated ECs in PDO-9 silenced or not for QKI and cultured in minimal media. PDOs were electroporated with the indicated siRNAs (40 nM) and collected after 4 days. Analysis of QKI expression level is shown.
See also Figures S5 and S6 and Tables S2, S4, and S5.

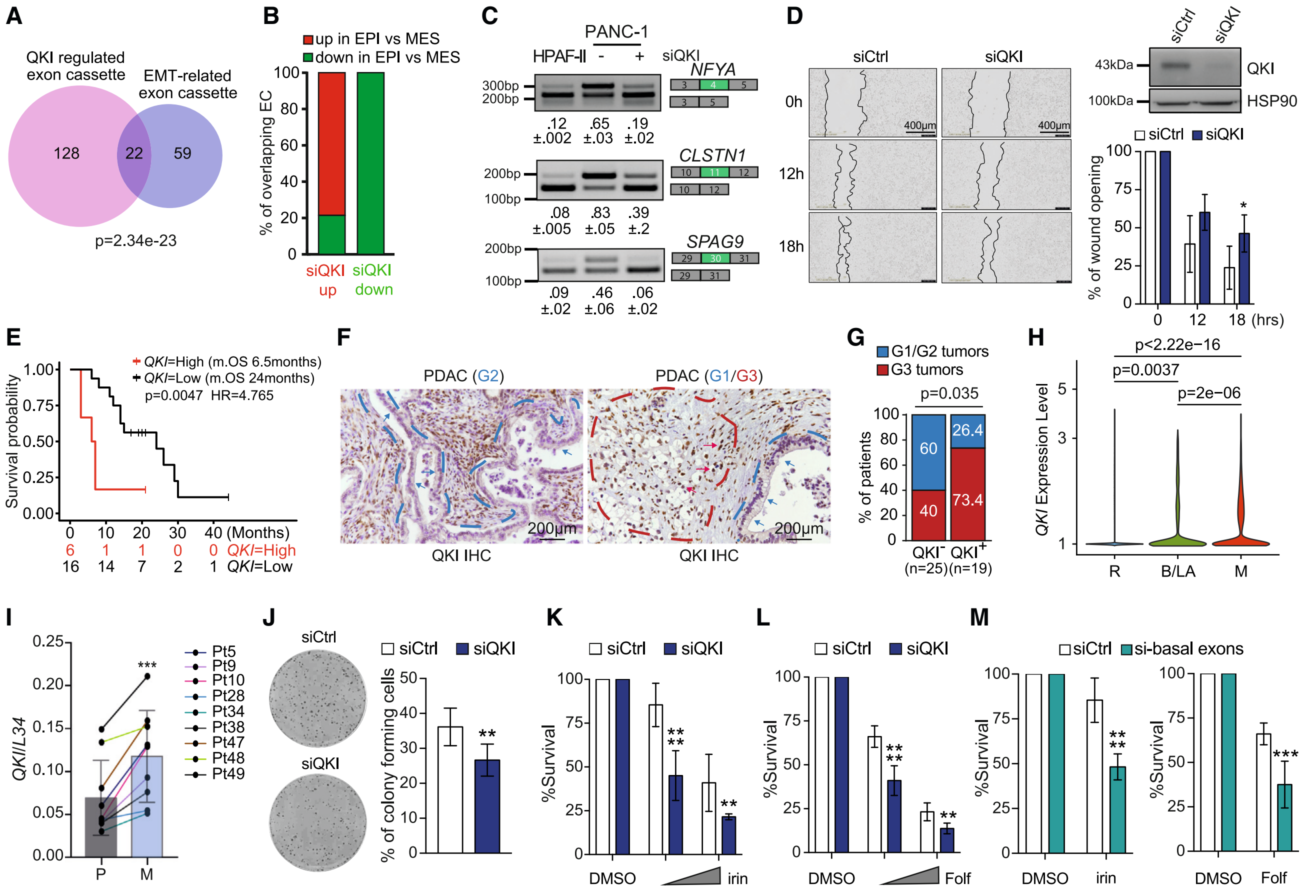

Figure 7. QKI promotes a pro-mesenchymal (MES) phenotype in PDAC

(A) Overlap between ECs regulated by QKI and ECs regulated during EMT in breast cancer.[43] Statistical analysis was performed by the hypergeometric test.

(B) EC events present in the overlap in (A) showing the type of regulation (up or down) in breast epithelial (EPI) cells with respect to MES cells. Events were separated between those up- or down-regulated in QKI-depleted PANC-1 cells.

(C) Splicing assays of the *NFYA*, *CLSTN1*, and *SPAG9* ECs in HPAF-II and in PANC-1 depleted or not for QKI. Mean ± SD of the PSI value (n = 3) is reported below the panels.

(D) Wound-healing assays in PANC-1 cells silenced for QKI (left). Scale bar: 400 μm. Silencing of QKI expression was assessed by western blot analysis (right). The histograms report the quantification of wound area. Data are reported as the mean ± SD (n = 3; two-way ANOVA test; *p < 0.05).

(E) Kaplan-Meier curve displaying the OS of patients with PDAC exhibiting high (third and fourth upper quartiles, red line; n = 6) or low (first and second quartiles, black line; n = 16) QKI expression, as determined by real-time qPCR in EUS-TA samples.

(F) IHC analysis of QKI in patients with PDAC. Dashed lines highlight the area of tissue containing G1/G2 tumors (blue) or G3 tumor (red). Arrows point to representative individual G1/G2 tumor cells (blue) or G3 tumor cells (red). Scale bar: 200 μm.

(G) Percentage of G1/G2 (blue) and G3 (red) tumors that were positive (QKI$^+$) or negative (QKI$^-$) for QKI expression by the IHC analysis. Statistical analysis was performed by the Fisher's test.

(H) Violin plot of QKI expression in resectable (R), borderline/locally advance (B/LA), and metastatic (M) tumors as determined by single-cell transcriptomic analysis. Statistical analysis was performed by Wilcoxon's test.

(I) QKI expression levels in primary lesion and liver metastasis from nine patients with PDAC (Student's t test; *p < 0.05 and **p < 0.01).

(J) Clonogenic assays of PANC-1 cells silenced or not for QKI after 10 days. The histogram reports the percentage of seeded cells that formed colonies (Student's t test; *p < 0.05 and **p < 0.01).

(K–M) Analysis of cell sensitivity performed by crystal violet in PANC-1 cells silenced for QKI (K and L) or for three basal-like ECs (*SEC16A*, *SYTL2*, and *LRRFIP2*) (M). Treatments were carried out for 6 days with increasing concentrations of irinotecan or mFOL (n = 3; mean ± SD; two-way ANOVA test; *p < 0.05, **p < 0.01, ***p = 0.001, and ****p < 0.0001).

See also Figure S7 and Tables S2 and S6.

topoisomerase I inhibitor included in the mFOL regimen. QKI-depleted PANC-1 cells were more sensitive to irinotecan or to the mFOL drug combination than control cells (Figures 7K and 7L). Notably, expression of QKI in classical HPAF-II cells reduced their sensitivity to irinotecan (Figure S7M), and this effect was accompanied by the switch in splicing of the subtype-specific exons (Figures S7N and S7O). Moreover, selective silencing of three QKI-induced basal-like splice variants (*LRRFIP2* exon 23$^+$, *SEC16A* exon 33$^+$, and *SYTL2* exon 12$^+$) was sufficient to recapitulate the effects of QKI depletion

(Figures 7M and S7P). By contrast, QKI depletion did not affect sensitivity to gemcitabine and only mildly increased sensitivity to low doses of nab-paclitaxel (Figure S7Q). These results indicate that expression of the QKI-induced splice variants contribute to the resistance of basal-like PDAC cells to mFOL chemotherapy.

DISCUSSION

PDAC heterogeneity substantially contributes to tumor malignancy and hampers the development of efficacious targeted therapies. Transcriptomic analyses revealed the existence of classical and basal-like phenotypes, which differ in their responses to current therapies and clinical course. Herein, we show that alternative splicing further refines the definition of these subtypes. Furthermore, the subtype-specific splicing events identified in our study are strongly correlated with survival of patients. Our work also uncovers the role of QKI as a key regulator of the splicing signature associated with the basal-like subtype. Together with recent studies highlighting the contribution of other splicing factors to PDAC onset, chemotherapy resistance, and metastasis,[20–22] these findings point to splicing dysregulation as a crucial process during pancreatic tumorigenesis. They also suggest that QKI is a valuable target to counteract PDAC malignancy.

Splicing alterations in cancer are generated by mutations that affect splice site recognition in the respective genes or by altered expression of splicing factors, responsible for reprogramming splicing of multiple genes.[11,12] Cancer-associated splicing changes alter all the processes involved in tumorigenesis, including resistance to chemotherapy.[13,46] In PDAC, the splicing factor SRSF1 was recently shown to induce pancreatitis,[20] an event tightly associated with tumorigenesis.[47] Furthermore, SRSF1 accelerated neoplastic transformation in a PDAC mouse model, at least in part, by activation of the mitogen-activated protein kinase (MAPK) signaling pathway.[20] Interestingly, treatment of PDAC cells with gemcitabine, a first-line chemotherapeutic agent for PDAC, was shown to induce SRSF1 expression and splicing of MNK2b, a splice variant that overrides upstream regulatory control by the MAPK pathway.[48] Thus, up-regulation of SRSF1 promotes the early events in pancreatic tumorigenesis and allows established PDAC cells to withstand chemotherapeutic treatments. Moreover, RBFOX2 was identified as a determinant of the splicing events that are differentially regulated between primary and metastatic PDAC.[22] Expression of RBFOX2 in primary PDAC lesions was proposed to limit metastasis by altering the ratio of protein isoforms involved in cytoskeletal organization and focal adhesion formation.[22] We now show that the splicing signature of tumor cells can also be employed to accurately distinguish the PDAC molecular subtypes and that QKI is a key determinant of the basal-like splicing pattern. Several QKI-dependent splicing events, as well as QKI expression itself, were significantly associated with the prognosis of patients, suggesting that analysis of the splicing signature of PDAC samples holds important clinical relevance. Noteworthily, similar results were obtained by *in silico* analyses of transcriptomic data from resected PDAC samples and by direct evaluation of the splicing pattern in EUS-TA samples from an independent cohort. Such reproducibility is of particular importance because most patients with PDAC present at diagnosis with non-resectable tumors,[1] thus making EUS samples the only accessible resource for biomarker analysis.

While the two-subtype classification is useful to distinguish cases featuring different prognoses and responses to treatment,[25,26] it does not consider the elevated intratumor heterogeneity of PDAC. Single-cell transcriptomics and multiplex immunofluorescence analyses have demonstrated that most tumors contain cells of both classical and basal-like phenotypes.[6–9] In addition, many of these intermediate tumors also feature cells co-expressing markers of both subtypes at variable levels, thus creating a continuum between classical and basal-like phenotypes even within the same gland.[9] Such co-expressor cells may represent a plastic population that converts from the classical into the more aggressive basal-like phenotype. Mixed tumors showed an intermediate prognosis, with the percentage of basal-like cells within the tumor being correlated with negative outcome.[9] Our findings suggest that QKI contributes to this plasticity by promoting the basal-like splicing pattern in PDAC cells. Analysis of two independent single-cell transcriptomics datasets revealed that QKI expression is significantly increased in basal-like cells with respect to classical cells, showing an opposite distribution with respect to the classical marker GATA6. Interestingly, positivity for GATA6 displays the same potential to identify classical tumors as negativity for QKI (55% of cases). However, by combining these two markers, the percentage of tumors classified as classical increased to 76%. Although studies with larger cohorts are necessary to validate this observation, these findings suggest that concomitant assessment of GATA6 and QKI may provide a stronger diagnostic tool to determine the PDAC subtype. Moreover, based on the ratio between basal-like and classical cells in the lesion, a large percentage of QKI^+ tumors (50%) were classified as intermediate. These results suggest that QKI expression is associated with acquisition of a plastic phenotype by PDAC cells, which may allow them to switch toward a less differentiated and more malignant state. In support of this hypothesis, increased QKI expression was associated with the ability of a PDAC PDO to switch the splicing signature toward the basal-like pattern when cultured under conditions that promote the basal-like phenotype *in vitro*.[7] By contrast, a QKI-low PDO line was unable to undergo this splicing switch, further indicating that QKI contributes to the plasticity of PDAC cells. QKI expression was also significantly associated with a poorly differentiated morphology of PDAC, with many QKI^+ tumors also comprising more differentiated areas with cells that were negative for this splicing factor. Thus, up-regulation of QKI in PDAC cells is likely associated with transition to a plastic stage in which cells acquire an undifferentiated phenotype and are more responsive to external cues.

QKI expression was previously correlated with EMT in breast cancer cells.[49] QKI regulated the splicing of actin cytoskeleton-associated genes and promoted a pro-mesenchymal phenotype, cell migration, and invasion while restraining tumor growth *in vivo*.[49] Accordingly, the genes regulated at splicing level by QKI in PDAC cells are also enriched in EMT-related terms, and depletion of QKI impaired PDAC cell migration

and clonogenicity. Furthermore, increased QKI levels were detected in metastases with respect to matched primary lesions of patients with PDAC. Interestingly, partial activation of EMT by the transcription factor ZEB1 was associated with tumor cell plasticity and metastasis formation in a PDAC mouse model.[50] Herein, we show a highly significant correlation of QKI expression with that of ZEB1 and other EMT regulators in PDAC. Moreover, depletion of QKI inhibits ZEB1 expression and increases that of the epithelial marker CDH1, stabilizing the epithelial phenotype of PDAC cells. ZEB1 represses microRNAs (miR-200 family) that inhibit QKI expression in epithelial cells.[49] Thus, up-regulation of ZEB1 may induce QKI expression in PDAC cells and alter their splicing signature to favor a pro-mesenchymal, plastic phenotype, which allows them to escape from the organ and to disseminate to adjacent structures and/or distant sites.

Another important feature associated with cell plasticity and EMT is acquisition of chemoresistance.[51,52] EMT inhibition in PDAC mouse models enhanced sensitivity to chemotherapy and increased survival.[52] Our results indicate that QKI contributes to chemoresistance of quasi-mesenchymal PDAC cells. The QKI-regulated splicing program is enriched in genes regulated by irinotecan, one of the components of mFOL. Moreover, while basal-like tumors are resistant to mFOL treatment,[10] depletion of QKI in PANC-1 cells restored partial sensitivity to this combined regimen, as well as to irinotecan alone. Conversely, QKI expression in classical PDAC cells enhanced resistance to irinotecan. Direct silencing of the splice variants promoted by QKI also enhanced sensitivity to mFOL, indicating the role of the QKI-regulated splicing program in this process. Thus, our work suggests that QKI is an important factor associated with aggressiveness in PDAC by contributing to cell dedifferentiation and to the acquisition of a migratory, chemoresistant phenotype. Given the fast-growing development of RNA-based therapies,[53] it is conceivable that targeting the splicing program orchestrated by QKI in PDAC cells might be exploited to limit their plasticity and to freeze them into a state that is more susceptible to chemotherapy.

Limitations of the study

Although our study clearly points to a role for QKI in promoting a plastic, metastatic phenotype in PDAC, additional *in vivo* experiments, such as orthotopic xenograft models of PDAC cells depleted for QKI, are necessary to fully validate this role. Moreover, given the small sample size of our validation cohort, the diagnostic and prognostic potential of the splicing signature needs to be further assessed in larger cohorts of unselected patients with PDAC.

STAR★METHODS

Detailed methods are provided in the online version of this paper and include the following:

- KEY RESOURCES TABLE
- RESOURCE AVAILABILITY
  - Lead contact
  - Materials availability
  - Data and code availability
- EXPERIMENTAL MODEL AND STUDY PARTICIPANT DETAILS
- METHOD DETAILS
  - Cell culture, transfection, western blot and immunofluorescence analyses
  - RNA extraction and RT-PCR analyses
  - RNA-seq and bioinformatics analyses
  - Single cell transcriptomic analyses
  - Immunofluorescence analyses
  - Establishment of Cell lines Stably silenced for QKI and over-expressing QKI-GFP
  - UV-crosslinked and RNA immunoprecipitation (CLIP) experiments
  - SEC16A minigene assay
  - Immunohistochemical analysis
  - Patient-derived organoids culture
  - Electroporation of patients-derived organoids
  - Wound healing and adhesion assays
  - Cell proliferation, clonogenic and drug sensitivity assays
- QUANTIFICATION AND STATISTICAL ANALYSIS

SUPPLEMENTAL INFORMATION

Supplemental information can be found online at https://doi.org/10.1016/j.xcrm.2024.101411.

ACKNOWLEDGMENTS

We wish to thank Dr. C. Caggiano for help with the stable cell lines, Dr. D. Bonvissuto for help with IHC analysis, Prof. S. Richard for the generous gift of QKI reagents, and Prof. Sette's laboratory members for discussions throughout the study. We acknowledge the contribution of the GSTeP Organoids Research Core Facility of the IRCCS Fondazione Policlinico Gemelli for the development of PDAC organoids. The graphical abstract was created with BioRender. This work was supported by the Associazione Italiana Ricerca sul Cancro (AIRC IG23416 to C. Sette, IG 26330 to G.T., IG 26201 to G.C., MFAG 23681 to C.C., Fellowship 26812 to V.R., and Fellowship 23938 to V.P.), the Italian Ministry of University (MUR; PRIN 2022 Prot. 20223KJZCN), the Italian Ministry of Health (Ricerca Corrente 2022 and Ricerca Corrente 2023 to IRCCS Fondazione Policlinico A. Gemelli), and the Università Cattolica del Sacro Cuore (Linea D1 and D3.2). Università Cattolica del Sacro Cuore contributed to the funding of this research project and its publication. Funding for the open access charge was provided by Associazione Italiana Ricerca sul Cancro, Università Cattolica del Sacro Cuore, and Ministero della Salute Ricerca Corrente 2022 and Ricerca Corrente 2023.

AUTHOR CONTRIBUTIONS

Conceptualization, C. Sette, V.R., and C.N.; writing – original draft, review & editing, C. Sette and V.R.; investigation, V.R., M.P., A.L., E.C., C.D., and V.P.; data curation, M.P., C.N., C.A., F.B., F.A., C. Spada, G.Q., S.A., G.T., L.A., P.G.A., M.F., G.C., C.C., and A.K.; supervision, C. Sette; funding acquisition, C. Sette. The authors read and approved the final manuscript.

DECLARATION OF INTERESTS

The authors declare no competing interests.

Received: August 3, 2023
Revised: November 19, 2023
Accepted: January 12, 2024
Published: February 6, 2024

REFERENCES

1. Neoptolemos, J.P., Kleeff, J., Michl, P., Costello, E., Greenhalf, W., and Palmer, D.H. (2018). Therapeutic developments in pancreatic cancer: Current and future perspectives. Nat. Rev. Gastroenterol. Hepatol. *15*, 333–348.
2. Mizrahi, J.D., Surana, R., Valle, J.W., and Shroff, R.T. (2020). Pancreatic cancer. Lancet *395*, 2008–2020.
3. Javed, M.A., Beyer, G., Le, N., Vinci, A., Wong, H., Palmer, D., Morgan, R.D., Lamarca, A., Hubner, R.A., Valle, J.W., et al. (2019). Impact of intensified chemotherapy in metastatic pancreatic ductal adenocarcinoma (PDAC) in clinical routine in Europe. Pancreatology *19*, 97–104.
4. Collisson, E.A., Bailey, P., Chang, D.K., and Biankin, A.V. (2019). Molecular subtypes of pancreatic cancer. Nat. Rev. Gastroenterol. Hepatol. *16*, 207–220.
5. Schreyer, D., Neoptolemos, J.P., Barry, S.T., and Bailey, P. (2021). Deconstructing Pancreatic Cancer Using Next Generation-Omic Technologies–From Discovery to Knowledge-Guided Platforms for Better Patient Management. Front. Cell Dev. Biol. *9*, 795735.
6. Peng, J., Sun, B.-F., Chen, C.-Y., Zhou, J.-Y., Chen, Y.-S., Chen, H., Liu, L., Huang, D., Jiang, J., Cui, G.-S., et al. (2019). Single-cell RNA-seq highlights intra-tumoral heterogeneity and malignant progression in pancreatic ductal adenocarcinoma. Cell Res. *29*, 725–738.
7. Raghavan, S., Winter, P.S., Navia, A.W., Williams, H.L., DenAdel, A., Lowder, K.E., Galvez-Reyes, J., Kalekar, R.L., Mulugeta, N., Kapner, K.S., et al. (2021). Microenvironment drives cell state, plasticity, and drug response in pancreatic cancer. Cell *184*, 6119–6137.e26.
8. Werba, G., Weissinger, D., Kawaler, E.A., Zhao, E., Kalfakakou, D., Dhara, S., Wang, L., Lim, H.B., Oh, G., Jing, X., et al. (2023). Single-cell RNA sequencing reveals the effects of chemotherapy on human pancreatic adenocarcinoma and its tumor microenvironment. Nat. Commun. *14*, 797.
9. Williams, H.L., Dias Costa, A., Zhang, J., Raghavan, S., Winter, P.S., Kapner, K.S., Ginebaugh, S.P., Väyrynen, S.A., Väyrynen, J.P., Yuan, C., et al. (2023). Spatially Resolved Single-Cell Assessment of Pancreatic Cancer Expression Subtypes Reveals Co-expressor Phenotypes and Extensive Intratumoral Heterogeneity. Cancer Res. *83*, 441–455.
10. O'Kane, G.M., Grünwald, B.T., Jang, G.H., Masoomian, M., Picardo, S., Grant, R.C., Denroche, R.E., Zhang, A., Wang, Y., Lam, B., et al. (2020). GATA6 Expression Distinguishes Classical and Basal-like Subtypes in Advanced Pancreatic Cancer. Clin. Cancer Res. *26*, 4901–4910.
11. Desterro, J., Bak-Gordon, P., and Carmo-Fonseca, M. (2020). Targeting mRNA processing as an anticancer strategy. Nat. Rev. Drug Discov. *19*, 112–129.
12. Sette, C., and Paronetto, M.P. (2022). Somatic Mutations in Core Spliceosome Components Promote Tumorigenesis and Generate an Exploitable Vulnerability in Human Cancer. Cancers *14*, 1827.
13. Paronetto, M.P., Passacantilli, I., and Sette, C. (2016). Alternative splicing and cell survival: From tissue homeostasis to disease. Cell Death Differ. *23*, 1919–1929.
14. Wright, C.J., Smith, C.W.J., and Jiggins, C.D. (2022). Alternative splicing as a source of phenotypic diversity. Nat. Rev. Genet. *23*, 697–710.
15. Kahles, A., Lehmann, K.-V., Toussaint, N.C., Hüser, M., Stark, S.G., Sachsenberg, T., Stegle, O., Kohlbacher, O., Sander, C., Rätsch, G., and Rätsch, G. (2018). Comprehensive Analysis of Alternative Splicing Across Tumors from 8,705 Patients. Cancer Cell *34*, 211–224.e6.
16. Smart, A.C., Margolis, C.A., Pimentel, H., He, M.X., Miao, D., Adeegbe, D., Fugmann, T., Wong, K.-K., and Van Allen, E.M. (2018). Intron retention is a source of neoepitopes in cancer. Nat. Biotechnol. *36*, 1056–1058.
17. Lu, S.X., De Neef, E., Thomas, J.D., Sabio, E., Rousseau, B., Gigoux, M., Knorr, D.A., Greenbaum, B., Elhanati, Y., Hogg, S.J., et al. (2021). Pharmacologic modulation of RNA splicing enhances anti-tumor immunity. Cell *184*, 4032–4047.e31.
18. Jun, Y., Suh, Y.-S., Park, S., Lee, J., Kim, J.-I., Lee, S., Lee, W.-P., Anczuków, O., Yang, H.-K., and Lee, C. (2022). Comprehensive Analysis of Alternative Splicing in Gastric Cancer Identifies Epithelial-Mesenchymal Transition Subtypes Associated with Survival. Cancer Res. *82*, 543–555.
19. Escobar-Hoyos, L.F., Penson, A., Kannan, R., Cho, H., Pan, C.H., Singh, R.K., Apken, L.H., Hobbs, G.A., Luo, R., Lecomte, N., et al. (2020). Altered RNA Splicing by Mutant p53 Activates Oncogenic RAS Signaling in Pancreatic Cancer. Cancer Cell *38*, 198–211.e8.
20. Wan, L., Lin, K.-T., Rahman, M.A., Ishigami, Y., Wang, Z., Jensen, M.A., Wilkinson, J.E., Park, Y., Tuveson, D.A., and Krainer, A.R. (2023). Splicing Factor SRSF1 Promotes Pancreatitis and KRASG12D-Mediated Pancreatic Cancer (Cancer Discov). https://doi.org/10.1158/2159-8290.CD-22-1013.
21. Calabretta, S., Bielli, P., Passacantilli, I., Pilozzi, E., Fendrich, V., Capurso, G., Fave, G.D., and Sette, C. (2016). Modulation of PKM alternative splicing by PTBP1 promotes gemcitabine resistance in pancreatic cancer cells. Oncogene *35*, 2031–2039.
22. Jbara, A., Lin, K.-T., Stossel, C., Siegfried, Z., Shqerat, H., Amar-Schwartz, A., Elyada, E., Mogilevsky, M., Raitses-Gurevich, M., Johnson, J.L., et al. (2023). RBFOX2 modulates a metastatic signature of alternative splicing in pancreatic cancer. Nature *617*, 147–153.
23. Alors-Perez, E., Blázquez-Encinas, R., Alcalá, S., Viyuela-García, C., Pedraza-Arevalo, S., Herrero-Aguayo, V., Jiménez-Vacas, J.M., Mafficini, A., Sánchez-Frías, M.E., Cano, M.T., et al. (2021). Dysregulated splicing factor SF3B1 unveils a dual therapeutic vulnerability to target pancreatic cancer cells and cancer stem cells with an anti-splicing drug. J. Exp. Clin. Cancer Res. *40*, 382.
24. Cancer Genome Atlas Research Network Electronic address andrew_aguirre@dfciharvardedu; Cancer Genome Atlas Research Network (2017). Integrated Genomic Characterization of Pancreatic Ductal Adenocarcinoma The Cancer Genome Atlas Research Network. Cancer Cell *32*, 185–203.e13.
25. Moffitt, R.A., Marayati, R., Flate, E.L., Volmar, K.E., Loeza, S.G.H., Hoadley, K.A., Rashid, N.U., Williams, L.A., Eaton, S.C., Chung, A.H., et al. (2015). Virtual microdissection identifies distinct tumor- and stroma-specific subtypes of pancreatic ductal adenocarcinoma. Nat. Genet. *47*, 1168–1178.
26. Rashid, N.U., Peng, X.L., Jin, C., Moffitt, R.A., Volmar, K.E., Belt, B.A., Panni, R.Z., Nywening, T.M., Herrera, S.G., Moore, K.J., et al. (2020). Purity Independent Subtyping of Tumors (PurIST), A Clinically Robust, Single-sample Classifier for Tumor Subtyping in Pancreatic Cancer. Clin. Cancer Res. *26*, 82–92.
27. Saraiva-Agostinho, N., and Barbosa-Morais, N.L. (2019). Psichomics: Graphical application for alternative splicing quantification and analysis. Nucleic Acids Res. *47*, e7.
28. Seino, T., Kawasaki, S., Shimokawa, M., Tamagawa, H., Toshimitsu, K., Fujii, M., Ohta, Y., Matano, M., Nanki, K., Kawasaki, K., et al. (2018). Human Pancreatic Tumor Organoids Reveal Loss of Stem Cell Niche Factor Dependence during Disease Progression. Cell Stem Cell *22*, 454–467.e6.
29. Hanahan, D. (2022). Hallmarks of Cancer: New Dimensions. Cancer Discov. *12*, 31–46.
30. Archibugi, L., Ruta, V., Panzeri, V., Redegalli, M., Testoni, S.G.G., Petrone, M.C., Rossi, G., Falconi, M., Reni, M., Doglioni, C., et al. (2020). RNA Extraction from Endoscopic Ultrasound-Acquired Tissue of Pancreatic Cancer Is Feasible and Allows Investigation of Molecular Features. Cells *9*, 2561.
31. Fu, X.D., and Ares, M. (2014). Context-dependent control of alternative splicing by RNA-binding proteins. Nat. Rev. Genet. *15*, 689–701.
32. Naro, C., Cesari, E., and Sette, C. (2021). Splicing regulation in brain and testis: common themes for highly specialized organs. Cell Cycle *20*, 480–489.
33. Diaferia, G.R., Balestrieri, C., Prosperini, E., Nicoli, P., Spaggiari, P., Zerbi, A., and Natoli, G. (2016). Dissection of transcriptional and cis -regulatory

CellPress
OPEN ACCESS

control of differentiation in human pancreatic cancer. EMBO J. *35*, 595–617.

34. Panzeri, V., Manni, I., Capone, A., Naro, C., Sacconi, A., Di Agostino, S., de Latouliere, L., Montori, A., Pilozzi, E., Piaggio, G., et al. (2021). The RNA-binding protein MEX3A is a prognostic factor and regulator of resistance to gemcitabine in pancreatic ductal adenocarcinoma. Mol. Oncol. *15*, 579–595.
35. Darbelli, L., and Richard, S. (2016). Emerging functions of the Quaking RNA-binding proteins and link to human diseases. Wiley Interdiscip. Rev. RNA *7*, 399–412.
36. Conboy, J.G. (2017). Developmental regulation of RNA processing by Rbfox proteins. Wiley Interdiscip. Rev. RNA *8*.
37. Hafner, M., Landthaler, M., Burger, L., Khorshid, M., Hausser, J., Berninger, P., Rothballer, A., Ascano, M., Jr., Jungkamp, A.-C., Munschauer, M., et al. (2010). Transcriptome-wide identification of RNA-binding protein and microRNA target sites by PAR-CLIP. Cell *141*, 129–141.
38. Lovci, M.T., Ghanem, D., Marr, H., Arnold, J., Gee, S., Parra, M., Liang, T.Y., Stark, T.J., Gehman, L.T., Hoon, S., et al. (2013). Rbfox proteins regulate alternative mRNA splicing through evolutionarily conserved RNA bridges. Nat. Struct. Mol. Biol. *20*, 1434–1442.
39. Zong, F.-Y., Fu, X., Wei, W.-J., Luo, Y.-G., Heiner, M., Cao, L.-J., Fang, Z., Fang, R., Lu, D., Ji, H., and Hui, J. (2014). The RNA-binding protein QKI suppresses cancer-associated aberrant splicing. PLoS Genet. *10*, e1004289.
40. Yeo, G.W., Coufal, N.G., Liang, T.Y., Peng, G.E., Fu, X.-D., and Gage, F.H. (2009). An RNA code for the FOX2 splicing regulator revealed by mapping RNA-protein interactions in stem cells. Nat. Struct. Mol. Biol. *16*, 130–137.
41. Pradella, D., Naro, C., Sette, C., and Ghigna, C. (2017). EMT and stemness: flexible processes tuned by alternative splicing in development and cancer progression. Mol. Cancer *16*, 8.
42. Espinet, E., Klein, L., Puré, E., and Singh, S.K. (2022). Mechanisms of PDAC subtype heterogeneity and therapy response. Trends Cancer *8*, 1060–1071.
43. Tranchevent, L.C., Aubé, F., Dulaurier, L., Benoit-Pilven, C., Rey, A., Poret, A., Chautard, E., Mortada, H., Desmet, F.O., Chakrama, F.Z., et al. (2017). Identification of protein features encoded by alternative exons using Exon Ontology. Genome Res. *27*, 1087–1097.
44. Beck, B., and Blanpain, C. (2013). Unravelling cancer stem cell potential. Nat. Rev. Cancer *13*, 727–738.
45. Kuleshov, M.V., Jones, M.R., Rouillard, A.D., Fernandez, N.F., Duan, Q., Wang, Z., Koplev, S., Jenkins, S.L., Jagodnik, K.M., Lachmann, A., et al. (2016). Enrichr: a comprehensive gene set enrichment analysis web server 2016 update. Nucleic Acids Res. *44*, W90–W97.
46. Bradley, R.K., and Anczuków, O. (2023). RNA splicing dysregulation and the hallmarks of cancer. Nat. Rev. Cancer *23*, 135–155.
47. Del Poggetto, E., Ho, I.-L., Balestrieri, C., Yen, E.-Y., Zhang, S., Citron, F., Shah, R., Corti, D., Diaferia, G.R., Li, C.-Y., et al. (2021). Epithelial memory of inflammation limits tissue damage while promoting pancreatic tumorigenesis. Science *373*, eabj0486.
48. Adesso, L., Calabretta, S., Barbagallo, F., Capurso, G., Pilozzi, E., Geremia, R., Delle Fave, G., and Sette, C. (2013). Gemcitabine triggers a pro-survival response in pancreatic cancer cells through activation of the MNK2/eIF4E pathway. Oncogene *32*, 2848–2857.
49. Pillman, K.A., Phillips, C.A., Roslan, S., Toubia, J., Dredge, B.K., Bert, A.G., Lumb, R., Neumann, D.P., Li, X., Conn, S.J., et al. (2018). miR-200/375 control epithelial plasticity-associated alternative splicing by repressing the RNA-binding protein Quaking. EMBO J. *37*, e99016.
50. Krebs, A.M., Mitschke, J., Lasierra Losada, M., Schmalhofer, O., Boerries, M., Busch, H., Boettcher, M., Mougiakakos, D., Reichardt, W., Bronsert, P., et al. (2017). The EMT-activator Zeb1 is a key factor for cell plasticity and promotes metastasis in pancreatic cancer. Nat. Cell Biol. *19*, 518–529.
51. Genovese, G., Carugo, A., Tepper, J., Robinson, F.S., Li, L., Svelto, M., Nezi, L., Corti, D., Minelli, R., Pettazzoni, P., et al. (2017). Synthetic vulnerabilities of mesenchymal subpopulations in pancreatic cancer. Nature *542*, 362–366.
52. Zheng, X., Carstens, J.L., Kim, J., Scheible, M., Kaye, J., Sugimoto, H., Wu, C.C., LeBleu, V.S., and Kalluri, R. (2015). Epithelial-to-mesenchymal transition is dispensable for metastasis but induces chemoresistance in pancreatic cancer. Nature *527*, 525–530.
53. Paunovska, K., Loughrey, D., and Dahlman, J.E. (2022). Drug delivery systems for RNA therapeutics. Nat. Rev. Genet. *23*, 265–280.
54. Paronetto, M.P., Achsel, T., Massiello, A., Chalfant, C.E., and Sette, C. (2007). The RNA-binding protein Sam68 modulates the alternative splicing of Bcl-x. J. Cell Biol. *176*, 929–939.
55. Naro, C., De Musso, M., Delle Monache, F., Panzeri, V., de la Grange, P., and Sette, C. (2021). The oncogenic kinase NEK2 regulates an RBFOX2-dependent pro-mesenchymal splicing program in triple-negative breast cancer cells. J. Exp. Clin. Cancer Res. *40*, 397.
56. Panzeri, V., Pieraccioli, M., Cesari, E., de la Grange, P., and Sette, C. (2023). CDK12/13 promote splicing of proximal introns by enhancing the interaction between RNA polymerase II and the splicing factor SF3B1. Nucleic Acids Res. *gkad258. d*. *51*, 5512–5526.
57. Hao, Y., Hao, S., Andersen-Nissen, E., Mauck, W.M., 3rd, Zheng, S., Butler, A., Lee, M.J., Wilk, A.J., Darby, C., Zager, M., et al. (2021). Integrated analysis of multimodal single-cell data. Cell *184*, 3573–3587.e29.
58. Bielli, P., and Sette, C. (2017). Analysis of in vivo Interaction between RNA Binding Proteins and Their RNA Targets by UV Cross-linking and Immunoprecipitation (CLIP) Method. Bio. Protoc. *7*, e2274.
59. Pieraccioli, M., Caggiano, C., Mignini, L., Zhong, C., Babini, G., Lattanzio, R., Di Stasi, S., Tian, B., Sette, C., and Bielli, P. (2022). The transcriptional terminator XRN2 and the RNA-binding protein Sam68 link alternative polyadenylation to cell cycle progression in prostate cancer. Nat. Struct. Mol. Biol. *29*, 1101–1112.

STAR★METHODS

KEY RESOURCES TABLE

REAGENT or RESOURCE	SOURCE	IDENTIFIER
Antibodies		
Anti-QKI	Santa Cruz Biotechnology	Cat # sc-517305, RRID AB_2941818
Anti-QKI	Sigma-Aldrich	Cat # HPA019123, RRID AB_1855980
Anti-RBFOX2	Bethyl	Cat # A300864A, RRID AB_609476
Anti-RBM47	Sigma-Aldrich	Cat # SAB2104562, RRID AB_10669135
Anti-HSP90	Santa Cruz Biotechnology	Cat # sc-13119, RRID AB_675659
Anti-Ki-67	Abcam	Cat # Ab16667, RRID AB_302459
Anti- KRT18	Sigma-Aldrich	Cat # HPA001605, RRID AB_2666381
Anti-MUC13	Thermo Fisher Scientific	Cat # PA5-104505 RRID AB_2853806
Anti-mouse Alexa Fluor 594	Thermo Fisher Scientific	Cat # A11005, RRID AB_2534073
Anti-rabbit Alexa Fluor 488	Thermo Fisher Scientific	Cat # A11008, RRID AB_143165
Biological samples		
Tumor biopsies	This study	N/A
PDAC organoids	This study	N/A
Chemicals, peptides, and recombinant proteins		
Fetal bovine serum	Thermo Fisher Scientific	Cat # 10270-106
Non-Essential Amino acids 100x	Aurogene	Cat # AU-X0557-100
Gentamycin	Aurogene	Cat # AU-L0011-100
Penicillin and streptomycin	Aurogen	Cat # AU-L0022-100
Lipofectamine RNAiMAX	Thermo Fisher Scientific	Cat # 13778-150
Protease-Inhibitor Cocktail	Sigma-Aldrich	Cat #P8340
M-MLV reverse transcriptase	Promega	Cat #M1705
Random hexamers	Roche	Cat # 11034731001
GoTaq	Promega	Cat #M7845
SYBR Green I Master	Roche	Cat # 04887352001
Hematoxylin	Diapath	Cat #C0303
AdDF+++	Thermo Fisher Scientific	Cat # 12634-010
Primocin	Invivogen	Cat # ANT-PM-1
Triple express	Thermo Fisher Scientific	Cat # 12605-028
Opti-MEM	Thermo Fisher Scientific	Cat # 31985-047
Crystal violet	Sigma-Aldrich	Cat #C3886-100G
Irinotecan	MCE	Cat # HY-16562/CS-1138
5′ Fluoruracil	Sigma-Aldrich	Cat #F6627
Oxaliplatin	MCE	Cat # HY-17371/CS-0992
Triton X-100	Sigma-Aldrich	Cat #X100-12
Hoechst 33342	Thermo Fisher Scientific	Cat #H3570
Incucyte Nuclight Rapid NIR Dye	Sartorius	Cat # 4804
Lipofectamine 2000	Thermo Fisher Scientific	Cat # 11668-019
Polybrene	Sigma-Aldrich	Cat #H9269
Puromycin	Thermo Fisher Scientific	Cat # ANT-PR-1
RNasin	Promega	Cat #N251B
Protein G dynabeads	Thermo Fisher Scientific	Cat # 1004D
RNaseI	Thermo Fisher Scientific	Cat # AM2295

(*Continued on next page*)

Continued

REAGENT or RESOURCE	SOURCE	IDENTIFIER
Proteinase K	Applichem	Cat # A3830,0100
Phusion Hot Start High-Fidelity DNA polymerase	Thermo Fisher Scientific	Cat # F-530L
Glutamax	Thermo Fisher Scientific	Cat # 35050038
HEPES	Thermo Fisher Scientific	Cat # 15630-056
Y-27632	Tocris	Cat # 1254
Collagenase II	Thermo Fisher Scientific	Cat # 1701-015
Dispase	Sigma-Aldrich	Cat #D4818
DNAse I	Sigma-Aldrich	Cat # 10104159001
Cultrex growth factor reduced BME type 2	Bio-Techne	Cat # 3533-010-02
EGF	Peprotech	Cat # AF-100-15
B27	Thermo Fisher Scientific	Cat # 17504-44
Nicotinamide	Sigma-Aldrich	Cat #N0636
Gastrin	Biogems	Cat # 1003377
FGF10	Peprotech	Cat # 100-26
N-acetyl-L-cysteine	Sigma-Aldrich	Cat # A9165
Noggin	Peprotech	Cat #120-10C
MluI	NEB	Cat #R0198S
NotI	NEB	Cat #R0189S
BamHI	NEB	Cat #R0136S
**Critical commercial assays**		
Geneaid Minikit	Geneaid	Cat # RBD300
RNEasy mini kit	Qiagen	Cat # 74104
DNase I	Geneaid	Cat # XH12505
Bio-Rad protein assay	Bio-Rad	Cat # 5000006
miRNeasy micro kit	Qiagen	Cat # 1071023
Red Blood cell lysis	Roche	Cat # 11814389001
**Deposited data**		
RNA-seq data from human PDAC tumors	The Cancer Genome Atlas,[24] http://firebrowse.org/	TCGA, PAAD
RNA-seq data from human PDAC cell lines	This study	GSE234737
Single-cell RNA-seq data from human PDAC tumors	Gene Expression Omnibus,[6]	GSE205013
Single-cell RNA-seq data from human PDAC tumors	Genome Sequence Archive,[8]	CRA001160
**Experimental models: Cell lines**		
HPAF-II	Laboratory stock	N/A
Capan-1	Laboratory stock	N/A
PANC-1	Laboratory stock	N/A
MiaPaCa-2	Laboratory stock	N/A
HEK293T	Laboratory stock	N/A
**Oligonucleotides**		
See Table S7 for oligonucleotide sequences used in this study	This study	N/A
**Recombinant DNA**		
pCMV-dR8.2-dvpr	Laboratory Stock	N/A
pCMV-VSV-G	Laboratory Stock	N/A
pLKO.1 QKI	Laboratory Stock	N/A
pCMV6-QKI5-MYC-FLAG	Origene	Cat #RC205779
pLenti-C-mGFP-p2a-Puro	Origene	Cat #PS100093
pLenti-C-QKI5-mGFP-p2a-Puro	This study	N/A

(*Continued on next page*)

Continued		
REAGENT or RESOURCE	SOURCE	IDENTIFIER
pCI-*SEC16* exon32-exon 34 WT minigene	This study	N/A
pCI-*SEC16* exon32-exon 34 RBFOX2-mut1 minigene	This study	N/A
pCI-*SEC16* exon32-exon 34 RBFOX2-mut2 minigene	This study	N/A
pCI-*SEC16* exon32-exon 34 QKI-mut1 minigene	This study	N/A
pCI-*SEC16* exon32-exon 34 QKI-mut2 minigene	This study	N/A
Software and algorithms		
cBioPortal		www.cbioportal.org
R V4.3.0	CRAN	www.cran.r-project.org/
Python V2.7.17	Python Software Foundation	https://www.python.org/
ImageJ software V1.51 – MRI Wound Healing tool	Montpellier Resources Imagerie	https://github.com/Montpellier RessourcesImagerie/imagej_macros_and_scripts/wiki/Wound-Healing-Tool
Psichomics V1.12.1	Saraiva-Agostinho N. et al., 2019[27]	https://www.bioconductor.org/packages/release/bioc/html/psichomics.html
GraphPad Software		https://www.graphpad.com/
FastQC V.0.11.9	Babraham Institute Bioinformatics Group	https://www.bioinformatics.babraham.ac.uk/projects/fastqc
Picard V3.0.0	Broad Institute	https://broadinstitute.github.io/picard/
RSeQC V4.0.0		https://rseqc.sourceforge.net/
STAR V2.7.9.a		https://github.com/alexdobin/STAR
featureCounts V2.0.3		https://subread.sourceforge.net/
Seurat V4.3.0	CRAN	www.cran.r-project.org/
survival V3.2-11	CRAN	www.cran.r-project.org/
DREME V5.4.1	MEME SUITE	https://meme-suite.org/doc/dreme.html
Enrichr	Kuleshov, M. V et al., 2016[45]	https://maayanlab.cloud/Enrichr/
Image Lab	Biorad	https://www.bio-rad.com/it-it/product/image-lab-software?ID=KRE6P5E8Z
clipplotr V1.0.0		https://github.com/ulelab/clipplotr
Other		
NEPA21 electroporator	Nepagene	N/A

RESOURCE AVAILABILITY

Lead contact

Further information and requests for resources and reagents should be directed to and will be fulfilled by the lead contact, Claudio Sette (claudio.sette@unicatt.it).

Materials availability

Plasmids generated in this study will be made available upon reasonable request to the lead contact, Claudio Sette (claudio.sette@unicatt.it).

Data and code availability

- RNA-seq data are available on GEO database (accession number GSE234737).
- This paper does not report original code.
- Any additional information required to reanalyze the data reported in this paper is available from the lead contact upon request.

CellPress
OPEN ACCESS

EXPERIMENTAL MODEL AND STUDY PARTICIPANT DETAILS

Upon approval by the Internal Review Board (IRB BIO-PANCREAS version 3 2021), Pancreatic ductal adenocarcinoma (PDAC) patients specimens were collected at San Raffaele Research Hospital. Naive patients with clinical history and radiologic imaging suggestive of PDAC were considered for enrollment. Patients were not involved in the design, or conduct, or reporting, or dissemination plans of our research. Upon informed consent, diagnostic Endoscopic Ultrasound (EUS) procedures were performed under deep sedation with intravenous infusion of Propofol (Diprivan, Zeneca, Germany), using a Pentax therapeutic linear echoendoscope (EG3870UTK, EG38J10UT) and Hitachi ultrasound platforms (Arietta 850, Arietta V70). EUS-tissue acquisition (TA) was performed using a 25G FNA needle (Expect Slimline, Boston Scientific) by slow-pull technique and RNA isolated, quantified and evaluated as previously described.[30]

For immunohistochemical analysis, specimens were obtained from a distinct cohort of 44 patients who underwent upfront surgery from March 2014 to December 2018 in the context of prospective clinical trials approved by the San Raffaele Scientific Institute Ethics Committee.

For PDAC organoid development tumor biopsies were collected from patients treated at Fondazione Policlinico Universitario A. Gemelli IRCCS (FPG), Rome, Italy, from May 2022 to October 2022, upon informed consent. The protocol was approved by the Institutional Review Board (Protocol ID: 4121) and conducted in accordance with the Helsinki Declaration.

Demographic and clinical information of patients enrolled for this study are listed in Table S2.

METHOD DETAILS

Cell culture, transfection, western blot and immunofluorescence analyses

HPAF-II, Capan-1 and PANC-1 cells were grown in RPMI 1640 (Euroclone), MiaPaCa-2 cells were grown in DMEM (Sigma-Aldrich). All media were supplemented with 10% fetal bovine serum (FBS, Gibco), Non-Essential Amino Acids 100x (Thermo Fisher Scientific), gentamycin (Aurogene), penicillin and streptomycin (Aurogene). For RNA interference, cells were transfected with the indicated siRNAs (Table S1) using Lipofectamine RNAiMAX (Thermo Fisher Scientific) and harvested after 48 h for protein and RNA analyses. For western blot analyses, cell pellets were resuspended in RIPA buffer (Tris-HCl 50mM, NP40 1%, NaCl 150mM, Na-Deoxycholate 0.5%, EDTA 2mM, SDS 0.1%) supplemented with 2mM Na-orthovanadate, 0.5mM sodium fluoride, 1mM dithiothreitol and Protease-Inhibitor Cocktail (Sigma-Aldrich). Extracts were incubated 10 min on ice, sonicated for 5 s and centrifuged for 10 min at 13,000 rpm, 4°C. Supernatants were diluted in SDS-PAGE sample buffer, boiled for 10 min and analyzed as described.[54] Primary antibodies: anti-QKI (Santa Cruz Biotechnology sc-517305), anti-RBFOX2 (Bethyl A300-864A), anti-RBM47 (Sigma-Aldrich SAB2104562) and anti-HSP90 (Santa Cruz Biotechnology sc-13119).

RNA extraction and RT-PCR analyses

Total RNA was extracted using Geneaid Minikit (Geneaid). After digestion with DNase, 1 μg of RNA was reverse-transcribed using M-MLV reverse transcriptase (Promega) and random hexamers (Promega) and used as template for conventional PCR analyses using GoTaq enzyme (Promega), before analysis on agarose or acrylamide gels and quantification by densitometry using Image Lab Software (Biorad).[55] Real-time quantitative PCRs (qPCR) were performed using the SYBR Green I Master and the LightCycler 480 System (Roche). All primers used are listed in the Table S7.

RNA-seq and bioinformatics analyses

Gene expression and splicing analyses of data from TCGA (Firehose legacy) PDAC patients[24] were performed using the visual interface of the Psichomics R package.[27] Analysis were restricted to PDAC patients classified into basal-like and classical subtype and scored as high-purity tumor samples[24] (i.e., samples whose ABSOLUTE purity score ranged above the median value of the entire cohort PDAC). Psichomics R package[27] was also used for patient survival analysis according to the PSI of select AS events. The threshold PSI values of subtype-specific ECs used for separating the two groups were identified by employing the visual interface of the Psichomics R package, which is implemented to indicate the optimal cut-off that minimizes the p-value of the log rank test used to compare survival distribution.[27] For RNA-seq analyses, total RNA was extracted and DNase treated using the RNAeasy mini kit (QIAGEN) from PANC-1 cells transfected for 48 h with control, QKI- or RBFOX2-targeting siRNA. PolyA plus RNA-seq libraries were constructed and sequenced using a 150 bp paired-end format on an Illumina NovaSeq 6000 and analysis was performed as previously described.[55,56] Splicing analyses were performed considering only exon reads and flanking exon-exon junction reads ("EXON" analysis) in order to detect new potential alternative events and known patterns ("PATTERN" analysis) using the Human FAST DB v2022_1 splicing patterns annotation.[55,56] Results were considered statistically significant for p values $\leq$0.05 and fold-changes $\geq$1.5 for "PATTERN" analysis and p values $\leq$0.01 and fold-changes $\geq$2.0 for "EXON" analysis. Motif analysis was performed as previously described.[55] Correlation of RBPs expression with target genes in TCGA Firehose Legacy dataset was performed with the cBioPortal database (https://www.cbioportal.org/).

Single cell transcriptomic analyses

Single cell sequencing data from previously published datasets[6,8] were downloaded from the Gene Expression Omnibus (GSE205013, n = 27) and Genome Sequence Archive (CRA001160, n = 24). Data were pre-processed as described by the

authors including quality metrics and cell annotation using the R package Seurat version 4.3.0.[57] Briefly, data were filtered to include only high-quality cells, as defined by > 500 detectable genes, >1500 unique molecular identifiers, <15% of transcripts coming from mitochondrial genes, <1% of transcripts representing erythroid genes. Data were normalized to the total expression, multiplied by a scaling factor of 10,000, and log-transformed. Doublets/multiplets were removed using scDblFinder 1.6.0. To account for biological and technical batch differences between individual patients and scRNA-seq libraries, the Seurat anchor-based integration method for merging datasets was utilized. The 2000 most variable genes based on standardized variance were selected for canonical correlation analysis as an initial dimensional reduction. The integration anchors were then identified based on the first 30 dimensions and used to generate a new dimensional reduction for further analysis. To visualize the data, the dimensionality of the scaled integrated data matrix was further reduced to project the cells in two-dimensional space using principal component analysis followed by UMAP. The 30 nearest neighbors were used to define the local neighborhood size with a minimum distance of 0.3. The resulting PCAs were used for partitioning the dataset into clusters using a smart local moving community detection algorithm. A range of resolutions (0.1–1) was used to establish a sufficient number of clusters to separate known populations based on the expression of established markers. For analysis, only cells annotated as "Epithelial cluster" were used and cells of each patients were classified to the classical or basal-like subtypes based on their expression signature.[25] Samples with no clear enrichment for either subtype were defined as intermediate. UMAP and violin plots were generated using the R package Seurat version 4.3.0.[57]

Immunofluorescence analyses

For immunofluorescence analysis, PANC-1 cells were fixed with 4% paraformaldehyde and permeabilized with 0.5% Triton X-100. After 1 h (hr) at room temperature (RT) in blocking buffer (PBS, 5% BSA, 3% horse serum) and incubated over-night with anti-QKI antibody (1:500) or anti-RBFOX2 (1:200) in blocking buffer. After rinsing, secondary anti-mouse Alexa Fluor 594/anti-rabbit Alexa Fluor 488 (1:400; Thermo Fisher Scientific) antibodies diluted in PBS supplemented with 1% BSA were incubated for 1 h at 37°C. Nuclei were counterstained with Hoechst 33342. Images were minimally processed with Photoshop (Adobe) for composing panels.

Establishment of Cell lines Stably silenced for QKI and over-expressing QKI-GFP

The pLKO.1 plasmids containing short hairpin RNA (shRNA) sequences targeting QKI (5′ CCGAAGCTGGTTTAATCTATA 3′) and non-target control sequence were co-transfected with pCMV-dR8.2-dvpr and pCMV-VSV-G helper packaging plasmids into HEK293T cells using Lipofectamine 2000 (Thermo Fisher Scientific). After 48 h, the supernatant containing lentiviral particles was collected and centrifuged at 3000 rpm for 5 min. A 1:3 dilution of virus in the presence of polybrene (8 μg/mL) was added to PANC-1 cells plated at low density for 24 h before selection with puromycin (1 μg/mL) for 7 days. Selected cells were used for subsequent experiments.

Human QKI-5 wild type cDNA was subcloned from pCMV6-QKI5-MYC-FLAG (Origene, cat #RC205779) into the pLenti-C-mGFP-p2a-Puro vector (Origene cat #PS100093) using BamHI and MluI restriction enzymes. For lentiviral particles production, HEK293T cells were transfected with pCMV-dR8.2 dvpr, pCMV-VSV-G and pLenti-C-QKI5-mGFP-p2a-Puro using Lipofectamine 2000 (Thermo Fisher Scientific, Invitrogen). After 48 h lentiviral particles were collected and centrifuged at 3000 rpm for 5 min. HPAF-II cells were transduced with a 1:3 dilution of the lentiviral vector pLenti-C-mGFP-p2a-Puro or pLenti-C-QKI5-mGFP-p2a-Puro and maintained in RPMI supplemented with 1 mg/ml of Puromycin (Sigma). Selected cells were used for subsequent experiments.

UV-crosslinked and RNA immunoprecipitation (CLIP) experiments

CLIP assays were performed as previously described.[58,59] Briefly, PANC-1 cells were UV irradiated (400 mJ cm−2) in PBS on ice and collected by scraping in lysis buffer [50 mM Tris pH 8, 100 mM NaCl, 1 mM $MgCl_2$, 0.1 mM $CaCl_2$, 1% NP-40, 0.1% SDS, 0.5 mM Na_3VO_4, 1 mM TCEP, protease inhibitor cocktail and 30 U/ml RNasin (Promega)]. After sonication, the samples were incubated with RNase-free DNaseI (New England Biolabs) for 10 min at 37°C, centrifuged at 15,000g for 3 min at 4°C, and the supernatant was quantified with the Bio-Rad protein assay dye (Bio-Rad). Samples (1 mg) were immunoprecipitated for 2 h using 3 μg of anti-QKI, anti-RBFOX2 or control IgGs in the presence of protein G Dynabeads (Thermo Fisher Scientific and Invitrogen) and 10 μL of RNaseI (Ambion) diluted 1:1,000. After washes, samples were treated for 1 h with Proteinase K (50 μg) at 55°C and RNA isolated using the miRNeasy Micro Kit (Qiagen). The primers used for qPCR analysis are listed in Table S1. Results were represented as a percentage (%) of input (0.1 mg of extract).

SEC16A minigene assay

The *SEC16A* genomic region from exon 32 to exon 34 was amplified using Phusion Hot Start High-Fidelity DNA polymerase (Thermo Fisher Scientific) and cloned into the MluI/NotI restriction sites of pCI vector (Promega). The mutant minigenes for QKI- and RBFOX2-binding sites were generated by the megaprimer strategy. All primers are listed in Table S1. PANC-1 cells were transiently silenced with control, QKI- or RBFOX2-targeting siRNA as described above. After 48 h cells were transfected using Lipofectamine 2000 (Thermo Fisher Scientific) with 125 ng for each well of a 6-well plate when the confluency of cells was 70%.

Immunohistochemical analysis

Patients were aged between 18 and 75 years and naive to therapies, with a Karnofsky Performance Status of 70 or higher, and diagnosed with PDAC (Table S2). Sections were immunostained with anti-QKI polyclonal rabbit antibody (1:500; HPA019123,

Sigma-Aldrich) using a sensitive Bond Polymer Refine detection system in an automated Bond immuno-histochemistry instrument (Leica Biosystems, Germany) and counterstained with Haematoxylin. Nuclear QKI staining in neoplastic cells was evaluated to score tumors as QKI-positive or QKI negative. Scores were performed by an expert pathologist. Sections of paraffin-embedded PDOs were stained with the following antibodies: Ki-67 (Ab16667, Abcam), KRT18 (HPA001605, Sigma-Aldrich), MUC13 (PA5-104505, Invitrogen) and QKI (HPA019123, Sigma-Aldrich). The selected antibodies are well-established in the diagnostic routine laboratory, signals were clearly visible and captured by ordinary light microscope (Zeiss AxioPhot Microscope).

Patient-derived organoids culture

Tumor tissues were placed in 60 mm Petri dishes containing AdDF+++ culture medium (Advanced DMEM/F12 containing 1× Glutamax, 10 mM HEPES and antibiotics). Part of the tissue was fixed in formalin for histopathological and immunohistochemistry analysis, part was stored at−80°C for DNA/RNA isolation. The remaining part was minced by surgical blades into small fragments for organoids generation and digested in 10 mL AdDF+++ supplemented with 5 μM RHO/ROCK pathway inhibitor (Y-27632, Tocris) containing 2 mg/mL Collagenase II (Thermo Fisher Scientific), Dispase and DNAse I (Sigma-Aldrich) on an orbital shaker at 37°C for 1 h. The cell suspension was then applied to a MACS SmartStrainer (70 μm), placed on a 50 mL tube and washed with 10 mL of AdDF+ + +culture medium and centrifugated at 290 g. The pellet was incubated with 1 mL red blood cell lysis buffer for 5 min at room temperature to eliminate erythrocytes, followed by washing with culture medium and pelleting at 290 g. Cells were embedded in undiluted (100%) Cultrex growth factor reduced BME type 2 (Bio-Techne) on ice and 40 μL drops of BME cell suspension were allowed to solidify to a pre-warmed 24 well suspension culture plates (Greiner). The plate was placed at 37°C for 30 min to allow the Matrigel to polymerize before being overlaid with 500 μL of a growth factor cocktail medium [AdDF+ + +culture medium supplemented with EGF (50 ng/mL Peprotech), A83-01 (0.5 μM), B27 (1X GIBCO) and Primocin (1 mg/mL, InvivoGen), Y-27632 (9 μM Tocris), Wnt3a-conditioned medium (50% v/v), RSPO1-conditioned medium (10% v/v), Nicotinamide (10 mM, Sigma-Aldrich), Gastrin (10 nM, Biogems), fibroblast growth factor 10 (FGF10, 100 ng/mL, Peprotech), N-acetyl-L-cysteine (1 mM, Sigma-Aldrich) and Noggin (0.1 mg/mL, Peprotech)] and incubated at 37°C in humidified air containing 5% CO2. Medium was changed every 3–4 days and organoids were passaged every 1–2 weeks at suitable ratio (1:1 to 1:3).

Electroporation of patients-derived organoids

Organoids were cultured with standard or minimum medium (AdDF+++ culture medium supplemented with Primocin) for 9 days. For electroporation, organoids were dissociated to single cells by enzymatic digestion with Triple Express (Thermo Fisher Scientific) for 5–10 min at 37°C. 100-200 x 10^3 single cells were incubated with 40nM of the indicated siRNAs in a final volume of 100 μL of Opti-MEM for 1 min on ice, mixed with pipette and electroporated with a NEPA21 electroporator using a 2 mm cuvette and the following settings: Voltage (200 V Poring pulse- 20V Transfer pulse), Pulse length (5ms Poring pulse- 50ms Transfer pulse), Pulse interval (50ms Poring pulse- 50ms Transfer pulse), Number of pulses (2 Poring pulse- 5 Transfer pulse), Decay rate (10% Poring pulse- 40% Transfer pulse), Polarity (+ Poring pulse +/− Transfer pulse). Cells were diluted in 400 μL of ice-cold expansion medium, centrifuged for 8 min at 290g and plated in a drop of BME for additional 6 days.

Wound healing and adhesion assays

Control and QKI-depleted PANC-1 cells were plated at 100% of confluence and starved for 16 h. A wound was inflicted to the cell monolayer by scratching with a sterile pipette tip. Following two washes with PBS and addition of medium supplemented with 1% FBS, the plate was photographed at time 0 and at 12 and 18 h (hrs) after scratching. Wound area quantification was performed with ImageJ software using the MRI Wound Healing tool. For cell adhesion assays, 30,000 cells were plated at 37°C. After 20 min, unattached cells were rinsed away and attached cells were collected by trypsinization and counted in a Thoma's chamber. Results are represented as the mean ± standard deviation (SD) of three experiments, each performed in triplicate.

Cell proliferation, clonogenic and drug sensitivity assays

Indicated cells were seeded in a 96-well plate and imaged at 10× magnification in a IncuCyte SX5 Live-content imaging system (Essen Bioscience) at 37°C with 5% CO_2. Cells were labeled with Nuclight Rapid NIR (Sartorius) and images were acquired every 12 h for 6 days (four images/well) and analyzed using the IncuCyte Cell-by-Cell software to detect and quantify live cells. Colony-forming assays were performed by seeding in 700 PANC-1 cells silenced for QKI, or HPAF-II cells expressing QKI-GFP, in 6-multiwell. After 10 days, cells were fixed for 30 min with glutaraldehyde 6.0% (v/v)/crystal violet 0.5% (w/v) solution and colonies with n > 50 cells were counted. For drug sensitivity, the PANC-1 and HPAF-II cells were transfected with siRNAs for QKI or a combination of the three basal-like specific splice variants of the *LRRFIP2*, *SEC16A* and *SYTL2* genes, or QKI-GFP plasmid. After 24 h, cells were plated in 96 multiwell plates for drug assays. Cell survival was measured by crystal violet assay after 6 days of the indicated treatments. Irinotecan was added to the culture medium at the following concentrations: 2 μM and 6 μM. For mFOLFIRINOX treatment, a mixed solution of fluorouracil (0.4μM, 1.3μM), oxaliplatin (0.4μM, 1.3μM) and irinotecan (0.2μM, 0.7 μM) was dispensed.

QUANTIFICATION AND STATISTICAL ANALYSIS

Statistical analyses for differential gene expression, splicing changes, comparison of different datasets, motif analysis, survival analysis were performed in R, according to the statistical tests described in the STAR Methods Section and/or in the figure legends. Number of replicates and the appropriate statistical test used for the analysis of every experiment is indicated in the figure legend. Analyses were performed in GraphPad Prism and p value ≤ 0.05 was considered significant. For all tests, *: $p < 0.05$, **: $p < 0.01$, ***: $p < 0.001$, ****: $p < 0.0001$.

Cell Reports Medicine, Volume *5*

Supplemental information

An alternative splicing signature defines the basal-like phenotype and predicts worse clinical outcome in pancreatic cancer

Veronica Ruta, Chiara Naro, Marco Pieraccioli, Adriana Leccese, Livia Archibugi, Eleonora Cesari, Valentina Panzeri, Chantal Allgöwer, Paolo Giorgio Arcidiacono, Massimo Falconi, Carmine Carbone, Giampaolo Tortora, Federica Borrelli, Fabia Attili, Cristiano Spada, Giuseppe Quero, Sergio Alfieri, Claudio Doglioni, Alexander Kleger, Gabriele Capurso, and Claudio Sette

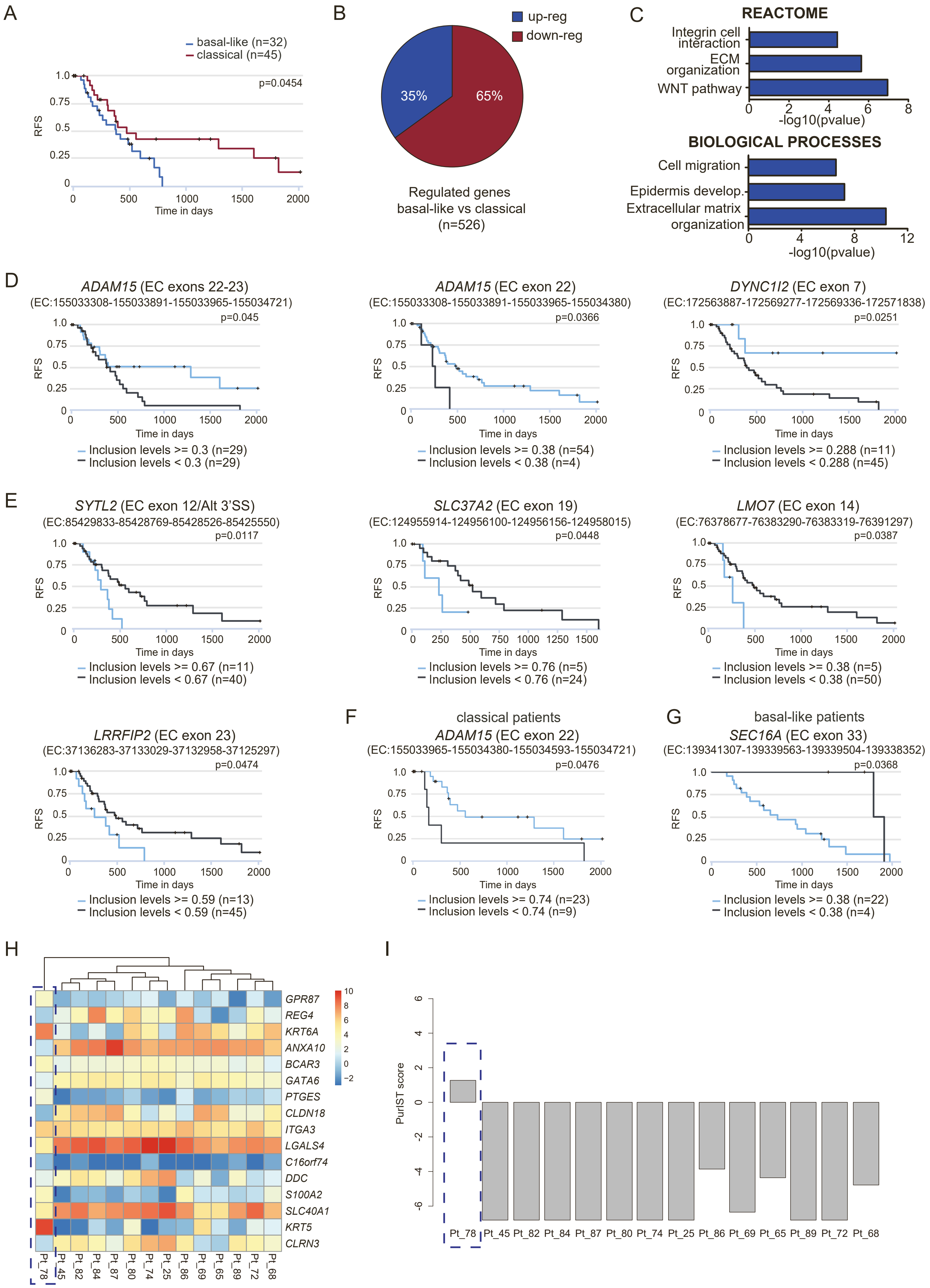

A
basal-like (n=32)
classical (n=45)
p=0.0454
RFS
Time in days
B
up-reg
down-reg
35%
65%
Regulated genes
basal-like vs classical
(n=526)
C
REACTOME
Integrin cell interaction
ECM organization
WNT pathway
-log10(pvalue)
BIOLOGICAL PROCESSES
Cell migration
Epidermis develop.
Extracellular matrix organization
-log10(pvalue)
D
ADAM15 (EC exons 22-23)
(EC:155033308-155033891-155033965-155034721)
p=0.045
Inclusion levels >= 0.3 (n=29)
Inclusion levels < 0.3 (n=29)
ADAM15 (EC exon 22)
(EC:155033308-155033891-155033965-155034380)
p=0.0366
Inclusion levels >= 0.38 (n=54)
Inclusion levels < 0.38 (n=4)
DYNC1I2 (EC exon 7)
(EC:172563887-172569277-172569336-172571838)
p=0.0251
Inclusion levels >= 0.288 (n=11)
Inclusion levels < 0.288 (n=45)
E
SYTL2 (EC exon 12/Alt 3'SS)
(EC:85429833-85428769-85428526-85425550)
p=0.0117
Inclusion levels >= 0.67 (n=11)
Inclusion levels < 0.67 (n=40)
SLC37A2 (EC exon 19)
(EC:124955914-124956100-124956156-124958015)
p=0.0448
Inclusion levels >= 0.76 (n=5)
Inclusion levels < 0.76 (n=24)
LMO7 (EC exon 14)
(EC:76378677-76383290-76383319-76391297)
p=0.0387
Inclusion levels >= 0.38 (n=5)
Inclusion levels < 0.38 (n=50)
LRRFIP2 (EC exon 23)
(EC:37136283-37133029-37132958-37125297)
p=0.0474
Inclusion levels >= 0.59 (n=13)
Inclusion levels < 0.59 (n=45)
F
classical patients
ADAM15 (EC exon 22)
(EC:155033965-155034380-155034593-155034721)
p=0.0476
Inclusion levels >= 0.74 (n=23)
Inclusion levels < 0.74 (n=9)
G
basal-like patients
SEC16A (EC exon 33)
(EC:139341307-139339563-139339504-139338352)
p=0.0368
Inclusion levels >= 0.38 (n=22)
Inclusion levels < 0.38 (n=4)
H
GPR87
REG4
KRT6A
ANXA10
BCAR3
GATA6
PTGES
CLDN18
ITGA3
LGALS4
C16orf74
DDC
S100A2
SLC40A1
KRT5
CLRN3
Pt_78 Pt_45 Pt_82 Pt_84 Pt_87 Pt_80 Pt_74 Pt_25 Pt_86 Pt_69 Pt_65 Pt_89 Pt_72 Pt_68
I
PurIST score
Pt_78 Pt_45 Pt_82 Pt_84 Pt_87 Pt_80 Pt_74 Pt_25 Pt_86 Pt_69 Pt_65 Pt_89 Pt_72 Pt_68

Figure S1: Subtype-specific genes and splicing events in PDAC patients. Related to Figures 1 and 2.
A) Kaplan-Meier curve displaying the RFS of PDAC patients from the whole TCGA cohort (n=77) segregated for classical and basal-like patients. **B)** Pie chart of the genes that are up-regulated (blue) and down-regulated (red) in basal-like vs classical PDAC. **C)** Pathways analysis by REACTOME and Gene Ontology analysis of Biological Processes of the genes that are up-regulated in basal-like PDAC. **D-E)** Kaplan-Meier curve displaying the RFS of PDAC patients from the whole TCGA cohort segregated for inclusion (blue line) or skipping (black line) of the indicated classical (**D**) and basal-like (**E**) exons. **F)** Kaplan-Meier curve displaying the RFS of classical PDAC patients from TCGA segregated for inclusion (blue line) or skipping (black line) of the *ADAM15* exon 22. **G)** Kaplan-Meier curve displaying the RFS of basal-like PDAC patients from TCGA segregated for inclusion (blue line) or skipping (black line) of the *SEC16A* exon 33. **H)** Heatmap showing subtype analysis in our cohort of EUS-TA PDAC patients (n=15) based on Moffitt's classification. **I)** The histogram represents the Purist score in each samples. **H,I)** The blue dashed boxes highlight the basal-like patient #78.

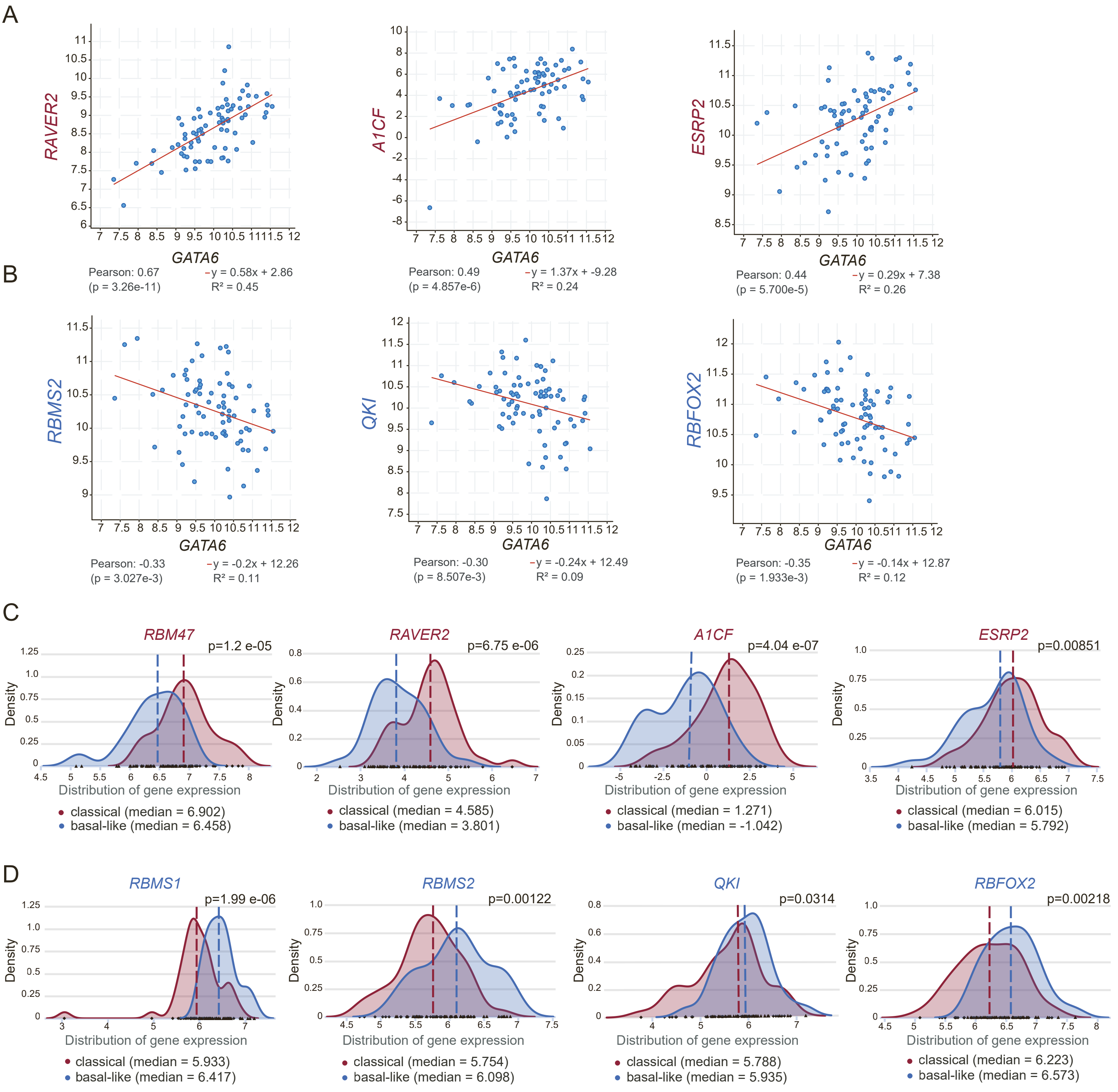

Figure S2: RBPs expression in PDAC patients and PDAC cell lines. Related to Figure 3.
A-B) Correlation analysis between the expression of classical (**A**) and basal-like (**B**) RBPs and that of GATA6. Statistical analysis was performed by 2-sided t-test. **C-D)** Density plot of the expression of classical (**C**) and basal-like (**D**) RBPs in classical (red) and basal-like (blue) PDAC patients from TCGA. Statistical analysis was performed by the Kruskal-Wallis rank sum test.

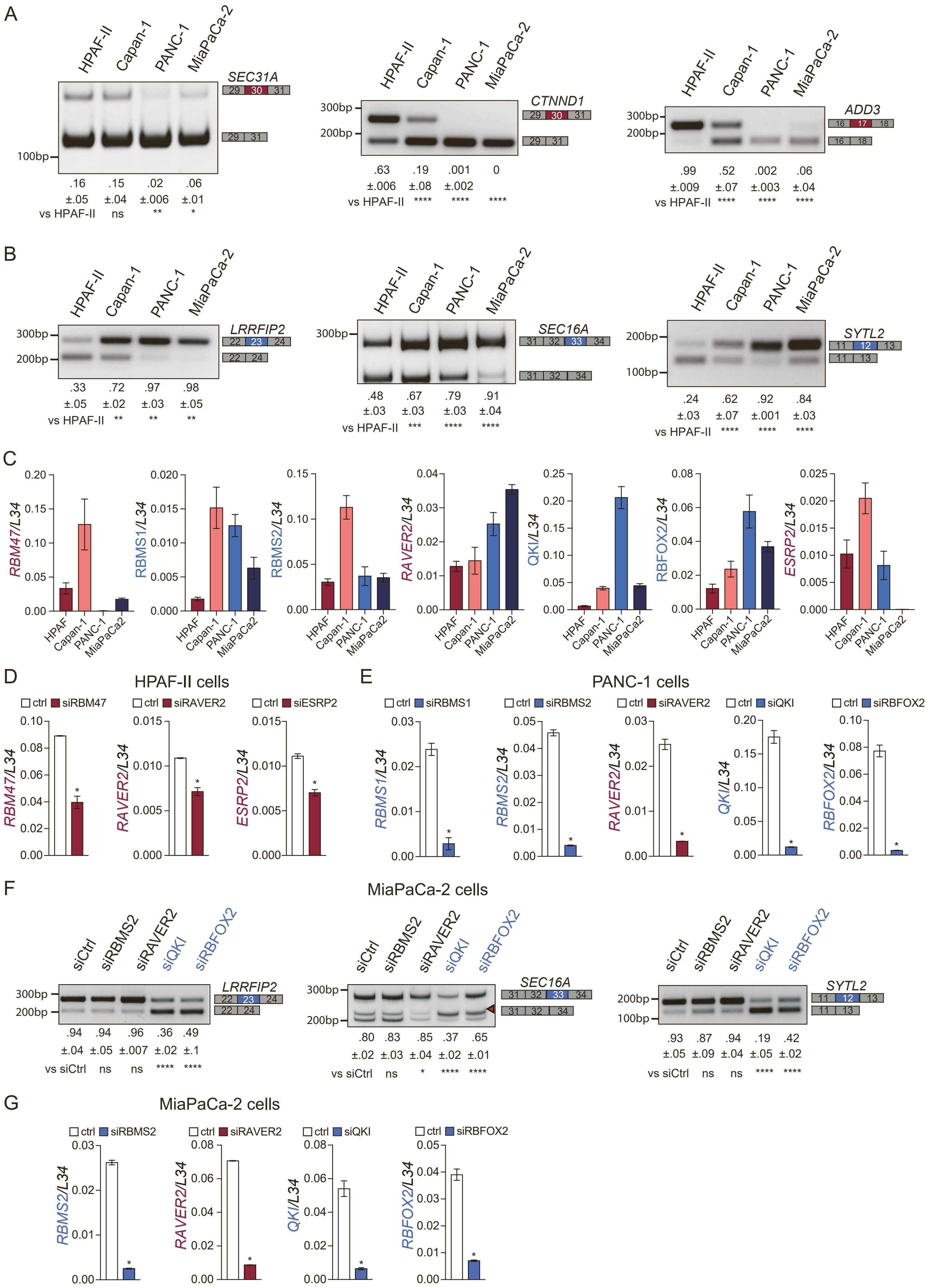

A
HPAF-II
Capan-1
PANC-1
MiaPaCa-2
SEC31A
29 30 31
29 31
100bp
.16 .15 .02 .06
±.05 ±.04 ±.006 ±.01
vs HPAF-II ns ** *
CTNND1
300bp
200bp
29 30 31
29 31
.63 .19 .001 0
±.006 ±.08 ±.002
vs HPAF-II **** **** ****
ADD3
16 17 18
16 18
.99 .52 .002 .06
±.009 ±.07 ±.003 ±.04
vs HPAF-II **** **** ****
B
LRRFIP2
22 23 24
22 24
.33 .72 .97 .98
±.05 ±.02 ±.03 ±.05
vs HPAF-II ** ** **
SEC16A
31 32 33 34
31 32 34
.48 .67 .79 .91
±.03 ±.03 ±.03 ±.04
vs HPAF-II *** **** ****
SYTL2
11 12 13
11 13
.24 .62 .92 .84
±.03 ±.07 ±.001 ±.03
vs HPAF-II **** **** ****
C
RBM47/L34
RBMS1/L34
RBMS2/L34
RAVER2/L34
QKI/L34
RBFOX2/L34
ESRP2/L34
HPAF
Capan-1
PANC-1
MiaPaCa2
D
HPAF-II cells
ctrl siRBM47
ctrl siRAVER2
ctrl siESRP2
E
PANC-1 cells
ctrl siRBMS1
ctrl siRBMS2
ctrl siRAVER2
ctrl siQKI
ctrl siRBFOX2
F
MiaPaCa-2 cells
siCtrl
siRBMS2
siRAVER2
siQKI
siRBFOX2
LRRFIP2
22 23 24
22 24
.94 .94 .96 .36 .49
±.04 ±.05 ±.007 ±.02 ±.1
vs siCtrl ns ns **** ****
SEC16A
31 32 33 34
31 32 34
.80 .83 .85 .37 .65
±.02 ±.03 ±.04 ±.02 ±.01
vs siCtrl ns * **** ****
SYTL2
11 12 13
11 13
.93 .87 .94 .19 .42
±.05 ±.09 ±.04 ±.05 ±.02
vs siCtrl ns ns **** ****
G
MiaPaCa-2 cells
ctrl siRBMS2
ctrl siRAVER2
ctrl siQKI
ctrl siRBFOX2
RBMS2/L34
RAVER2/L34
QKI/L34
RBFOX2/L34

Figure S3: Subtype-specific splicing events are differentially regulated in PDAC cell lines. Related to Figure 3.
A-B) Splicing assay of the indicated representative classical (A) and basal-like (B) ECs in classical (HPAF-II and Capan-1) and basal-like (PANC-1 and MiaPaCa-2) PDAC cell lines. Data represent the mean ± standard deviation (SD) of three independent experiments. Statistical analysis was performed by Student's t-test; *p<0.05, **p<0.01, ***p<0.001, ****p<0.0001. **C)** Expression analysis of the indicated RBPs in the HPAF-II, Capan-1, PANC-1 and MiaPaCa-2 cell lines. Data represent the mean ± S.D. of three independent experiments. **D,E)** qRT-PCR analysis of the expression level of *RBM47*, *RAVER2* and *ESPR2* in HPAF-II cells silenced or not for the indicated RBP (**D**) and of *RBMS1*, *RBMS2*, *RAVER2*, *QKI* and *RBFOX2* in PANC-1 cells silenced or not for the indicated RBP (**E**). **F)** Splicing assay of the indicated basal-like ECs in MiaPaCa-2 cells silenced for the indicated RBPs. **G)** qRT-PCR analysis of the expression level of *RBMS2*, *RAVER2*, *QKI* and *RBFOX2* in MiaPaCa-2 cells silenced or not for the indicated RBP, normalized with *L34* expression.

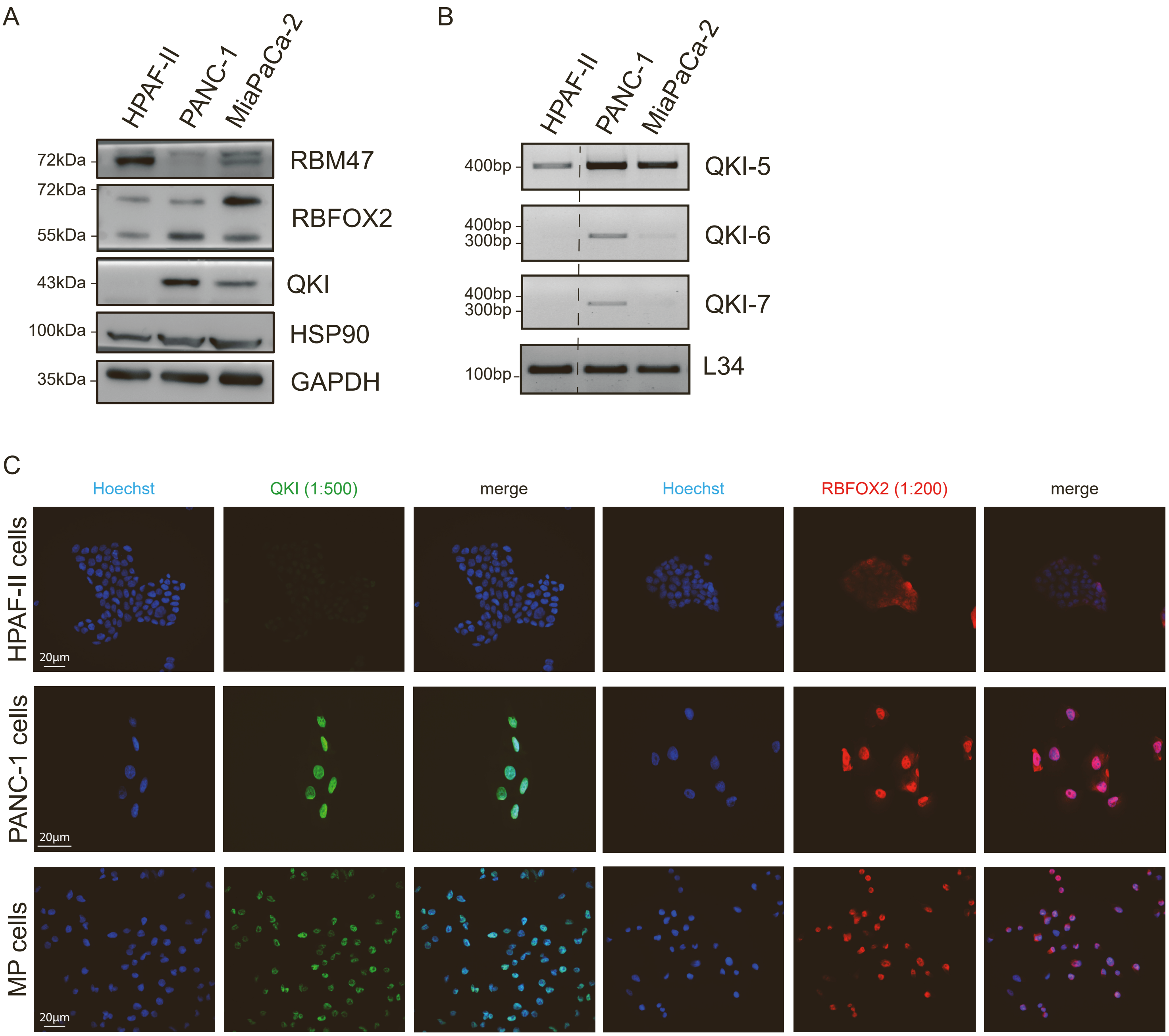

Figure S4: Expression of subtype specific RNA binding proteins in PDAC cell lines. Related to Figure 3 and 4.
A) Representative western blot analysis of the expression of RBM47, QKI and RBFOX2 in HPAF-II, PANC-1 and MiaPaCa-2 cells. HSP90 and GAPDH was used as loading controls. **B)** Representative RT-PCR analysis of the three QKI variants (QKI-5, QKI-6 and QKI-7) in HPAF-II, PANC-1 and MiaPaCa-2 cells. Dashed line indicates the border between images that have been computationally put in contact. **C)** Immunofluorescence analysis of the subcellular localization of QKI and RBFOX2 in HPAF-II, PANC-1 cells and MiaPaCa-2 cells. Scale bar=20µm.

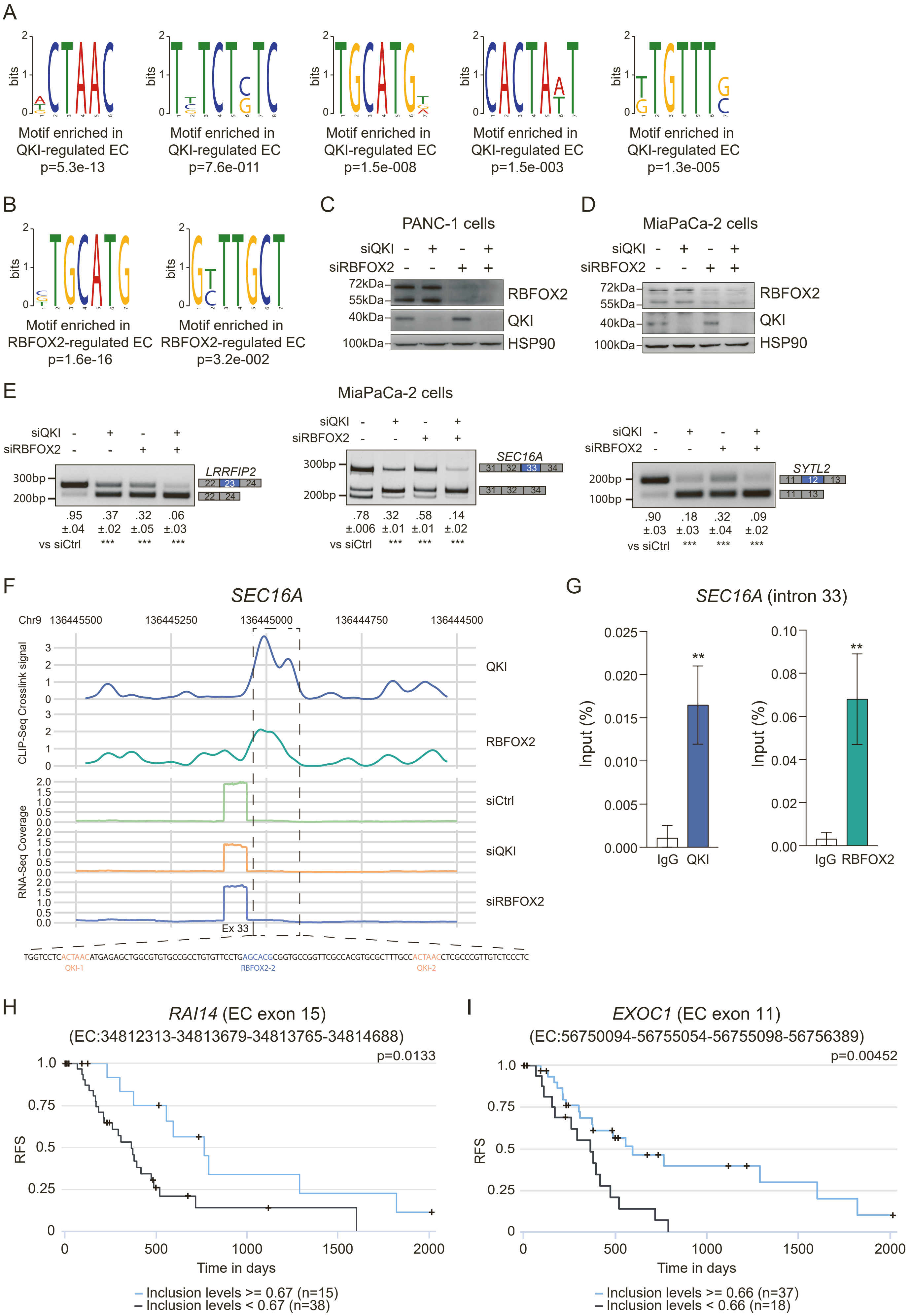

A
Motif enriched in QKI-regulated EC p=5.3e-13
Motif enriched in QKI-regulated EC p=7.6e-011
Motif enriched in QKI-regulated EC p=1.5e-008
Motif enriched in QKI-regulated EC p=1.5e-003
Motif enriched in QKI-regulated EC p=1.3e-005
B
Motif enriched in RBFOX2-regulated EC p=1.6e-16
Motif enriched in RBFOX2-regulated EC p=3.2e-002
C
PANC-1 cells
siQKI - + - +
siRBFOX2 - - + +
72kDa 55kDa RBFOX2
40kDa QKI
100kDa HSP90
D
MiaPaCa-2 cells
siQKI - + - +
siRBFOX2 - - + +
72kDa 55kDa RBFOX2
40kDa QKI
100kDa HSP90
E
MiaPaCa-2 cells
LRRFIP2
300bp 200bp
.95 ±.04 .37 ±.02 .32 ±.05 .06 ±.03
vs siCtrl *** *** ***
SEC16A
300bp 200bp
.78 ±.006 .32 ±.01 .58 ±.01 .14 ±.02
vs siCtrl *** *** ***
SYTL2
200bp 100bp
.90 ±.03 .18 ±.03 .32 ±.04 .09 ±.02
vs siCtrl *** *** ***
F
SEC16A
Chr9 136445500 136445250 136445000 136444750 136444500
CLIP-Seq Crosslink signal
QKI
RBFOX2
RNA-Seq Coverage
siCtrl
siQKI
siRBFOX2
Ex 33
TGGTCCTCACTAACATGAGAGCTGGCGTGTGCCGCCTGTGTTCCTGAGCACGCGGTGCCGGTTCGCCACGTGCGCTTTGCCACTAACCTCGCCCGTTGTCTCCCTC
QKI-1 RBFOX2-2 QKI-2
G
SEC16A (intron 33)
Input (%)
IgG QKI
IgG RBFOX2
**
H
RAI14 (EC exon 15)
(EC:34812313-34813679-34813765-34814688)
p=0.0133
RFS
Time in days
Inclusion levels >= 0.67 (n=15)
Inclusion levels < 0.67 (n=38)
I
EXOC1 (EC exon 11)
(EC:56750094-56755054-56755098-56756389)
p=0.00452
RFS
Time in days
Inclusion levels >= 0.66 (n=37)
Inclusion levels < 0.66 (n=18)

Figure S5: QKI and RBFOX2 bind intron 33 of SEC16A. Related to Figures 4, 5 and 6.
A-B) Logos representing the motifs significantly enriched in the regions flanking the ECs regulated by QKI (**A**) and RBFOX2 (**B**). **C-D)** Representative western blot analysis of the expression of RBFOX2 and QKI in PANC-1 and MiaPaCa-2 cells silenced or not for the indicated RBP. HSP90 was used as loading controls. **E)** Representative splicing assay (n=3) of the indicated basal-like ECs in MiaPaCa-2 cells silenced for QKI, RBFOX2 or both RBPs. Percentage of splicing inclusion ± SD (n=3) of the indicated exons was evaluated by densitometric analysis. Statistical analysis was performed by Student's t-test; ***p<0.001. **F)** Line plot showing mean normalized read coverage in two replicates of QKI and RBFOX2 eCLIP-seq using ENCODE project (upper panel) and mean normalized read coverage in three replicates of siCtrl, siQKI and siRBFOX2 RNA-seq (bottom panel) in a region encompassing the exon 33 region of the *SEC16A* gene. QKI and RBFOX2 binding region is highlighted with the dashed lines, their binding sites are highlighted in orange (QKI) and blue (RBFOX2). **G)** CLIP assay of QKI and RBFOX2 performed in PANC-1 cells. Immunoprecipitation with control IgGs was performed as negative control. RNA associated with *SEC16A* was quantified by qPCR using primers located at the *SEC16A* exon 33-intron 33 boundary. Results are represented as percentage (%) of input (mean ± SD of three biological replicates). Statistical analysis was performed by Student's t-test; **p<0.01. **H-I)** Kaplan-Meier curve displaying the RFS of PDAC patients from the TCGA cohort segregated for inclusion (blue line) or skipping (black line) of the *RAI14* (**H**) and *EXOC1* (**I**) ECs.

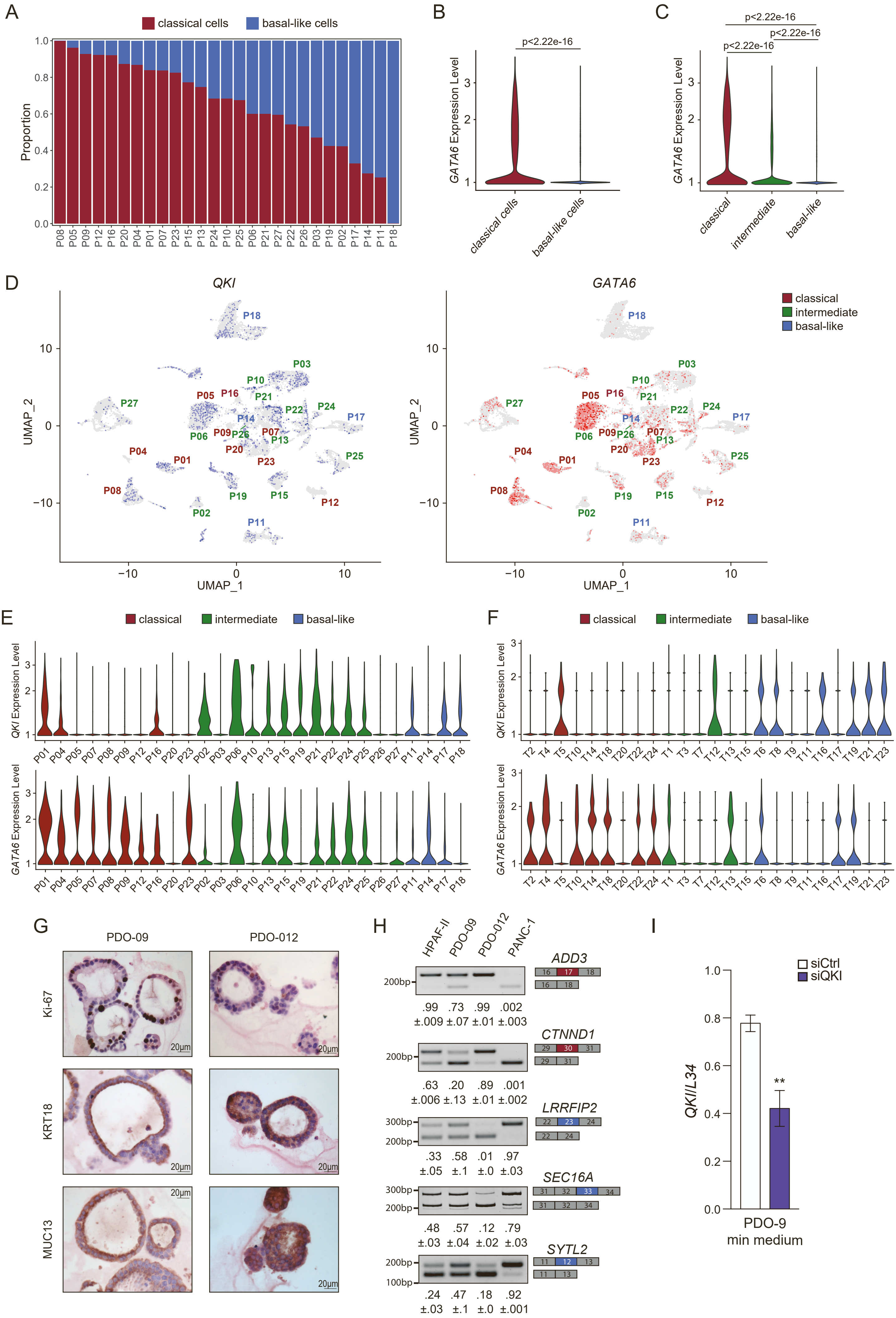

A
classical cells
basal-like cells
Proportion
1.0
0.8
0.6
0.4
0.2
0.0
P08 P05 P09 P12 P16 P20 P04 P01 P07 P23 P15 P13 P24 P10 P25 P06 P21 P27 P22 P26 P03 P19 P02 P17 P14 P11 P18
B
p<2.22e-16
GATA6 Expression Level
classical cells
basal-like cells
C
p<2.22e-16
p<2.22e-16
p<2.22e-16
GATA6 Expression Level
classical
intermediate
basal-like
D
QKI
GATA6
classical
intermediate
basal-like
UMAP_2
UMAP_1
E
classical
intermediate
basal-like
QKI Expression Level
GATA6 Expression Level
P01 P04 P05 P07 P08 P09 P12 P16 P20 P23 P02 P03 P06 P10 P13 P15 P19 P21 P22 P24 P25 P26 P27 P11 P14 P17 P18
F
classical
intermediate
basal-like
QKI Expression Level
GATA6 Expression Level
T2 T4 T5 T10 T14 T18 T20 T22 T24 T1 T3 T7 T12 T13 T15 T6 T8 T9 T11 T16 T17 T19 T21 T23
G
PDO-09
PDO-012
Ki-67
KRT18
MUC13
20µm
H
HPAF-II
PDO-09
PDO-012
PANC-1
ADD3
200bp
16 17 18
16 18
.99 .73 .99 .002
±.009 ±.07 ±.01 ±.003
CTNND1
200bp
29 30 31
29 31
.63 .20 .89 .001
±.006 ±.13 ±.01 ±.002
LRRFIP2
300bp
200bp
22 23 24
22 24
.33 .58 .01 .97
±.05 ±.1 ±.0 ±.03
SEC16A
300bp
200bp
31 32 33 34
31 32 34
.48 .57 .12 .79
±.03 ±.04 ±.02 ±.03
SYTL2
200bp
100bp
11 12 13
11 13
.24 .47 .18 .92
±.03 ±.1 ±.0 ±.001
I
siCtrl
siQKI
QKI/L34
1.0
0.8
0.6
0.4
0.2
0.0
**
PDO-9
min medium

Figure S6: Single cell analysis identifies QKI as a marker of the basal-like identity in PDAC and subtype-specific events are correlated with QKI expression in PDAC patient-derived organoids. Related to Figure 6.
A) Proportion of classical and basal-like cells identified according to the Moffitt signature in PDAC samples from the GSE205013 dataset. **B)** Violin plot of the gene expression levels of GATA6 in classical and basal-like cell population as determined by single cell transcriptomic analysis (GSE205013). **C)** Violin plot showing of the gene expression levels of GATA6 in tumors that were classified as classical, intermediate and basal-like as determined by single cell transcriptomic analysis (GSE205013). **D)** UMAP overview of the QKI (left) and GATA6 expression (right) in PDAC samples that were classified as classical, intermediate and basal-like on the basis of single cell transcriptome analysis using the Moffit signature (GSE205013). **E-F)** Violin plot of the gene expression levels of QKI and GATA6 in classical, intermediate and basal-like PDAC samples patients on single cell transcriptome analysis of the GSE205013 (**E**) and CRA001160 (**F**) datasets. **G)** IHC of Ki-67, KRT18 and MUC13 on PDAC PDO-9 and PDO-12. Scale bar=20µm. **H)** Splicing assays of *ADD3* (exon 17), *CTNND1* (exon 30), *LRRFIP2* (exon 23), *SYTL2* (exon 12) in two patient-derived organoids (PDO). For comparison the classical HPAF-II and the PANC-1 cell lines were also analyzed. **I)** qRT-PCR analysis of the expression level of QKI in PDO silenced or not for QKI.

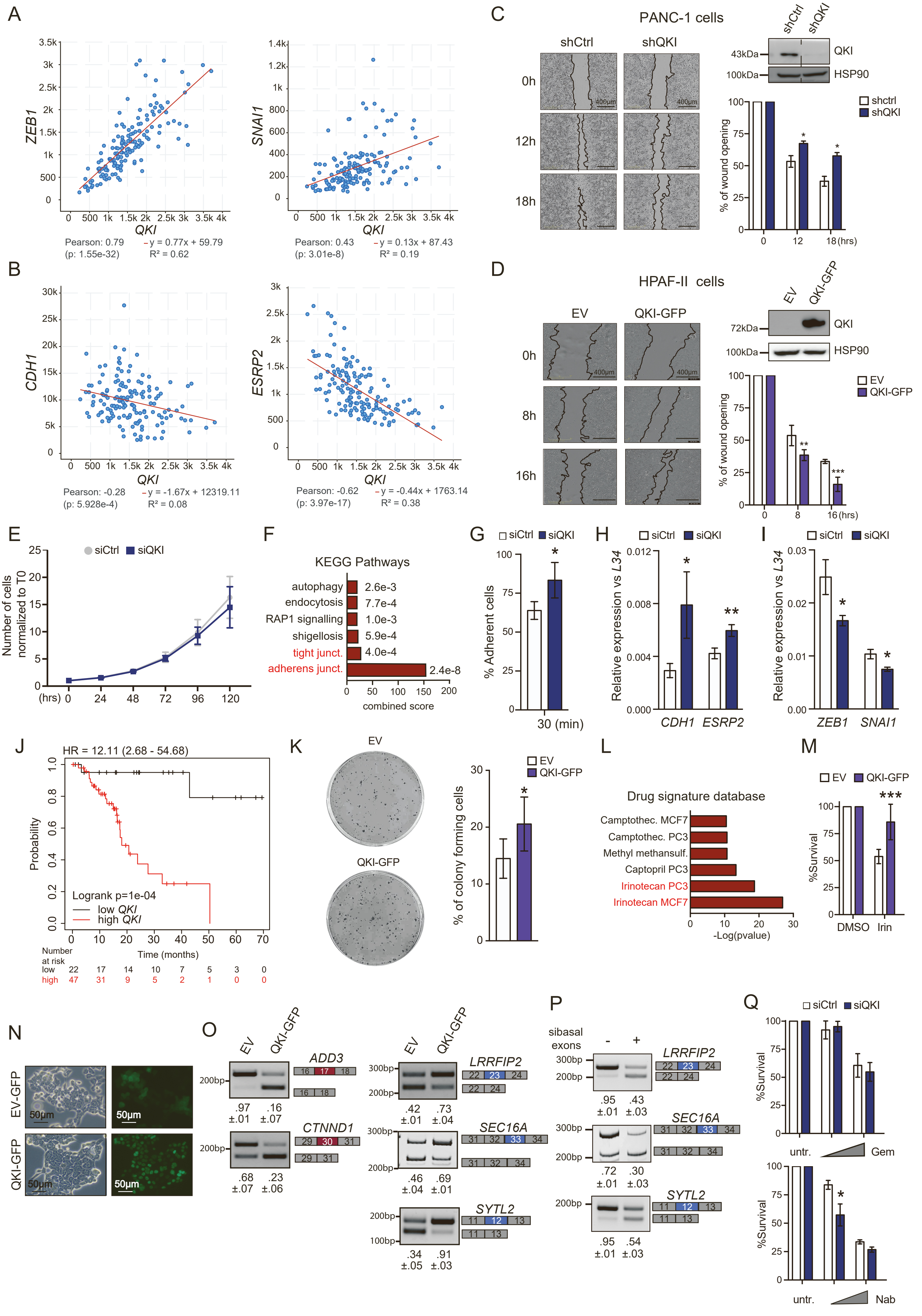

A
ZEB1
QKI
Pearson: 0.79
(p: 1.55e-32)
y = 0.77x + 59.79
R² = 0.62
SNAI1
QKI
Pearson: 0.43
(p: 3.01e-8)
y = 0.13x + 87.43
R² = 0.19
B
CDH1
QKI
Pearson: -0.28
(p: 5.928e-4)
y = -1.67x + 12319.11
R² = 0.08
ESRP2
QKI
Pearson: -0.62
(p: 3.97e-17)
y = -0.44x + 1763.14
R² = 0.38
C
PANC-1 cells
shCtrl
shQKI
0h
12h
18h
400μm
43kDa
QKI
100kDa
HSP90
shctrl
shQKI
% of wound opening
18 (hrs)
D
HPAF-II cells
EV
QKI-GFP
0h
8h
16h
72kDa
100kDa
EV
QKI-GFP
% of wound opening
16 (hrs)
E
siCtrl
siQKI
Number of cells normalized to T0
(hrs)
F
KEGG Pathways
autophagy 2.6e-3
endocytosis 7.7e-4
RAP1 signalling 1.0e-3
shigellosis 5.9e-4
tight junct. 4.0e-4
adherens junct. 2.4e-8
combined score
G
siCtrl
siQKI
% Adherent cells
30 (min)
H
Relative expression vs L34
CDH1
ESRP2
I
Relative expression vs L34
ZEB1
SNAI1
J
HR = 12.11 (2.68 - 54.68)
Probability
Logrank p=1e-04
low QKI
high QKI
Time (months)
Number at risk
low 22 17 14 10 7 5 3 0
high 47 31 9 5 2 1 0 0
K
EV
QKI-GFP
% of colony forming cells
L
Drug signature database
Camptothec. MCF7
Camptothec. PC3
Methyl methansulf.
Captopril PC3
Irinotecan PC3
Irinotecan MCF7
-Log(pvalue)
M
EV
QKI-GFP
%Survival
DMSO
Irin
N
EV-GFP
QKI-GFP
50μm
O
EV
QKI-GFP
ADD3
CTNND1
LRRFIP2
SEC16A
SYTL2
.97 ±.01
.16 ±.07
.68 ±.07
.23 ±.06
.42 ±.01
.73 ±.04
.46 ±.04
.69 ±.01
.34 ±.05
.91 ±.03
P
sibasal exons
LRRFIP2
SEC16A
SYTL2
.95 ±.01
.43 ±.03
.72 ±.01
.30 ±.03
.95 ±.01
.54 ±.03
Q
siCtrl
siQKI
%Survival
untr.
Gem
Nab

Figure S7: QKI promotes the mesenchymal phenotype in PDAC cells. Related to Figure 7.
A-B) Correlation analysis between the expression of *QKI* and that of pro-mesenchymal transcription factors *ZEB1* and *SNAI1* (**A**) and the epithelial markers *CDH1* and *ESRP2* (**B**) in PDAC samples from TCGA patients. **C)** Representative images of wound-healing assays in PANC-1 cells stably silenced for QKI. The histograms (right) report the quantification of the wound area (mean ± SD, n=3). Statistical analysis was performed by Two-way Anova test; *p<0.05. Western blot analysis to assess QKI silencing is shown above the histogram. Scale bar=400µm. **D)** Representative images of wound-healing assays in HPAF-II cells stably overexpressed QKI. The histograms (right) report the quantification of the wound area (mean ± SD, n=3). Statistical analysis was performed by Two-way Anova test; **p<0.01; ***p<0.001. Western blot analysis to assess QKI overexpression is shown above the histogram. Scale bar=400µm. **E)** Line graph showing growth of PANC-1 cells silenced or not for QKI. **F)** KEGG pathways analysis of the genes regulated by QKI at splicing level. **G)** Histogram reporting the percentage of adherent cells in PANC-1 silenced or not for QKI expression. Data are reported as the mean ± S.D. of three independent experiments. Statistical analysis was performed by the Student's t-test; *p<0.05. **H-I)** qRT-PCR showing the expression of the epithelial markers *CDH1*, *ESRP2* (**H**) and EMT-inducers *ZEB1*, *SNAI1* (**I**) in PANC-1 cells silenced or not for QKI (mean ± SD, n=3). Statistical analysis was performed by using the Student's t-test (*p<0.05; **p<0.01). **J)** Kaplan-Meier curve displaying overall survival of TCGA PDAC patients exhibiting high (upper quartiles; red line) or low (lower quartiles; black line) expression of QKI. **K)** Representative image of the colonies formed by HPAF-II cells expressing (QKI-GFP) or not (empty vector, EV) QKI. Images were taken after 10 days from seeding. The histogram reports the percentage of seeded cells that formed colonies. Statistical analysis was performed by using the Student's t-test (*p<0.05; **p<0.01). **L)** Enrichment score in the Drug signature database for the genes regulated by QKI at splicing level. **M)** Histograms displaying the analysis of cell sensitivity performed by crystal violet in HPAF-II cells expressing QKI-GFP treated or not with 2µM Irinotecan for 6 days. ***p<0.001 (Student's t-test) **N)** Images of HPAF-II cells transfected with GFP only (EV) or QKI-GFP. Scale bar=50µm. **O)** RT-PCR analysis of the QKI-regulated subtypespecific *ADD3*, *CTNND1*, *LRRFIP2*, *SYTL2* and *SEC16A* splice variants in HPAF-II cells transfected with QKI-GFP. **P)** RT-PCR analysis of the *LRRFIP2*, *SYTL2* and *SEC16A* splice variants in PANC-1 cells silenced or not for the corresponding basal like events. **Q)** Histograms showing the analysis of cell sensitivity performed by crystal violet in PANC-1 cells silenced for QKI. Treatments were carried out for 6 days with increasing concentration of gemcitabine (10nM, 15nM) or nab-paclitaxel (30nM, 50nM), or untreated (untr.). Results represent the mean ± SD of three experiments. Statistical analysis was performed by the Two-way ANOVA test (*p<0.05).
